# The MICA-129 dimorphism affects NKG2D signaling and outcome of hematopoietic stem cell transplantation

**DOI:** 10.15252/emmm.201505246

**Published:** 2015-10-19

**Authors:** Antje Isernhagen, Dörthe Malzahn, Elena Viktorova, Leslie Elsner, Sebastian Monecke, Frederike von Bonin, Markus Kilisch, Janne Marieke Wermuth, Neele Walther, Yesilda Balavarca, Christiane Stahl-Hennig, Michael Engelke, Lutz Walter, Heike Bickeböller, Dieter Kube, Gerald Wulf, Ralf Dressel

**Affiliations:** 1 Institute of Cellular and Molecular Immunology, University Medical Center GöttingenGöttingen, Germany; 2 Institute of Genetic Epidemiology, University Medical Center GöttingenGöttingen, Germany; 3 Department of Hematology and Medical Oncology, University Medical Center GöttingenGöttingen, Germany; 4 Institute of Molecular Biology, University Medical Center GöttingenGöttingen, Germany; 5 Unit of Infection Models, German Primate CenterGöttingen, Germany; 6 Primate Genetics Laboratory, German Primate CenterGöttingen, Germany

**Keywords:** cytotoxic T cells, graft-versus-host disease, NK-cell receptors, NK cells, single nucleotide polymorphism

## Abstract

The MHC class I chain-related molecule A (MICA) is a highly polymorphic ligand for the activating natural killer (NK)-cell receptor NKG2D. A single nucleotide polymorphism causes a valine to methionine exchange at position 129. Presence of a *MICA-129Met* allele in patients (*n* = 452) undergoing hematopoietic stem cell transplantation (HSCT) increased the chance of overall survival (hazard ratio [HR] = 0.77, *P *=* *0.0445) and reduced the risk to die due to acute graft-versus-host disease (aGVHD) (odds ratio [OR] = 0.57, *P *=* *0.0400) although homozygous carriers had an increased risk to experience this complication (OR = 1.92, *P *=* *0.0371). Overall survival of *MICA-129Val/Val* genotype carriers was improved when treated with anti-thymocyte globulin (HR = 0.54, *P *=* *0.0166). Functionally, the MICA-129Met isoform was characterized by stronger NKG2D signaling, triggering more NK-cell cytotoxicity and interferon-γ release, and faster co-stimulation of CD8^+^ T cells. The MICA-129Met variant also induced a faster and stronger down-regulation of NKG2D on NK and CD8^+^ T cells than the MICA-129Val isoform. The reduced cell surface expression of NKG2D in response to engagement by MICA-129Met variants appeared to reduce the severity of aGVHD.

## Introduction

Allogeneic hematopoietic stem cell transplantation (HSCT) offers an option to cure for several hematological diseases. Depending on the disease entity, 5-year survival rates vary, with limitations imposed by post-transplant complications, such as graft-versus-host disease (GVHD), relapse of malignancy, and infection (Dickinson, 2008). Human leukocyte antigen (HLA) matching is mandatory to reduce the risk of graft rejection and GVHD, but minor histocompatibility antigens also affect transplant outcome (Warren *et al*, 2012). Moreover, single nucleotide polymorphisms (SNPs) can influence the success of transplantations. SNPs in the tumor necrosis factor (TNF)-α or interleukin (IL)-6 genes, for example, have been associated with an increased risk of GVHD (Dickinson, 2008; Harris *et al*, 2013).

A candidate gene that could affect the outcome of HSCT is the major histocompatibility complex (MHC) class I chain-related A (*MICA*). It is the most polymorphic non-classical MHC class I gene in humans (Bahram *et al*, 1994; Leelayuwat *et al*, 1994; Choy & Phipps, 2010), and currently, 100 alleles are known encoding for 79 protein variants (http://www.ebi.ac.uk/imgt/hla/, release 3.17.0). The structure of MICA is similar to classical MHC class I molecules, but it is not associated with β2-microglobulin and does not present peptides. It is constitutively expressed on a few cell types, such as gastrointestinal epithelium (Groh *et al*, 1996) but becomes induced by cellular and genotoxic stress (Groh *et al*, 1996; Gasser *et al*, 2005) in malignant or virus-infected cells (Champsaur & Lanier, 2010; Raulet *et al*, 2013). MICA functions as ligand for the activating NK receptor NKG2D (NK group 2, member D; Bauer *et al*, 1999), which is present on NK cells, CD8^+^ αβT cells, subsets of CD4^+^ αβT cells, γδT cells, and NKT cells (Champsaur & Lanier, 2010; Raulet *et al*, 2013). While NKG2D signaling triggers cytotoxicity (Billadeau *et al*, 2003) and cytokine secretion in NK cells (Andre *et al*, 2004) and Vγ9Vδ2T cells (Rincon-Orozco *et al*, 2005), it is a co-stimulator for activation of CD8^+^ αβT cells (Groh *et al*, 2001). NKG2D is important for elimination of malignant cells (Guerra *et al*, 2008), contributes to rejection of mouse bone marrow grafts (Ogasawara *et al*, 2005), and is critical in defense against some pathogens (Fang *et al*, 2008; Wesselkamper *et al*, 2008; Champsaur & Lanier, 2010; Choy & Phipps, 2010).

*MICA* genotype matching and *MICB* genotype matching were associated with improved survival after HSCT (Kitcharoen *et al*, 2006), whereas mismatching was associated with increased incidence of acute GVHD (aGVHD; Parmar *et al*, 2009; Askar *et al*, 2014) although not in all studies (Anderson *et al*, 2009). The SNP at nucleotide position 454 (G/A) causing a valine (Val) to methionine (Met) exchange at amino acid position 129 in the α2 domain of the MICA protein was found to be associated with the incidence of chronic GVHD (cGVHD) and relapse (Boukouaci *et al*, 2009). This SNP has also been associated with the risks for nasopharyngeal carcinoma (Douik *et al*, 2009), hepatitis B virus-induced hepatocellular carcinoma (Tong *et al*, 2013), the severity of chronic Chagas heart disease (Ayo *et al*, 2015), and autoimmune diseases, including ankylosing spondylitis (Amroun *et al*, 2005), rheumatoid arthritis (Kirsten *et al*, 2009), inflammatory bowel disease (Lopez-Hernandez *et al*, 2010; Zhao *et al*, 2011), lupus erythematosus (Yoshida *et al*, 2011), type I diabetes (Raache *et al*, 2012), and psoriatic disease (Pollock *et al*, 2013). Moreover, the MICA-129 dimorphism is functionally relevant and the *MICA* alleles can be separated into two groups with respect to this polymorphism. MICA isoforms containing a methionine at position 129 have been characterized to bind NKG2D with high avidity, whereas those with a valine bind NKG2D with low avidity (Steinle *et al*, 2001). However, the finding that high-avidity MICA-129Met isoforms were associated with an increased incidence of relapse whereas the low-avidity isoforms were associated with an increased incidence of cGVHD (Boukouaci *et al*, 2009) appeared to be counterintuitive in view of the known functions of NKG2D. Therefore, we analyzed the MICA-129 dimorphism in an independent cohort of HSCT patients and investigated its functional effects beyond NKG2D binding to understand the mechanism how the SNP might impact the outcome of HSCT.

## Results

### Association of the MICA-129Val/Met dimorphism with the outcome of HSCT

The characteristics of 452 consecutive patients (P), who underwent allogeneic HSCT in the Department of Hematology and Medical Oncology of the University Medical Center Göttingen (UMG) between October 2002 and July 2013, and their donors (D) are shown in Table1. Recipients and donors were typed at high resolution for *HLA*-loci *A*, *B*, *C*, *DR*, and *DQ*, and a match of at least 7/8 loci at *HLA-A*, *B*, *DRB1,* and *DQB1* was considered eligible for transplantation. We analyzed these 452 P/D pairs for the SNP rs1051792, responsible for the MICA-129Val/Met dimorphism. Most P/D pairs (90.7%) had the same *MICA-129* genotype. About 54.4% of the patients experienced aGVHD (any grade) and 30.5% cGVHD (any grade), and in 19.0%, a relapse occurred. The overall mortality was 39.4%, and the treatment-related mortality (TRM) amounted to 24.1%. One reason for TRM was aGVHD, and 11.5% of the patients succumbed to aGVHD complications (Table1).

**Table 1:** HSCT pairs, diseases, transplantation characteristics, and outcome

Characteristics	Values
**Recipients (*n* = 452)**	
Median age, years (y)	52
Younger than 20 y, *n* (%)	7 (1.5)
20 to 40 y, *n* (%)	72 (15.9)
Older than 40 y, *n* (%)	373 (82.5)
Male, *n* (%)	275 (60.8)
MICA-129 genotype frequencies	
Val/Val, *n* (%)	232 (51.3)
Met/Val, *n* (%)	161 (35.6)
Met/Met, *n* (%)	59 (13.1)
MICA-129 allele frequencies	
Val, *n* (%)	625 (69.1)
Met, *n* (%)	279 (30.9)
**Underlying diagnosis**	
Acute leukemia, *n* (%)	180 (39.8)
Hodgkin lymphoma, non-Hodgkin lymphoma, *n* (%)	165 (36.5)
Multiple myeloma, *n* (%)	49 (10.8)
Myelodysplastic syndrome, *n* (%)	28 (6.2)
Myeloproliferative diseases, chronic myeloid leukemia, *n* (%)	15 (3.3)
Other diagnoses, *n* (%)	15 (3.3)
**Disease status for malignant disorders**	
Early, *n* (%)	94 (20.8)
Intermediate, *n* (%)	97 (21.5)
Advanced, *n* (%)	120 (26.5)
ND^a^, *n* (%)	141 (31.2)
**Donors (*n* = 452)**	
Median age, y	40
Younger than 20 y, *n* (%)	2 (0.4)
20 to 40 y, *n* (%)	209 (46.2)
Older than 40 y, *n* (%)	189 (41.8)
ND, *n* (%)	52 (11.5)
Male, *n* (%)	283 (62.6)
Female, *n* (%)	134 (29.6)
ND, *n* (%)	35 (7.7)
Female donor to male recipient, *n* (%)	77 (17.0)
HLA-matched unrelated donor, *n* (%)	307 (67.9)
Less than 8/8-matched unrelated donor, *n* (%)	68 (15.0)
Matched-related donor, *n* (%)	143 (31.6)
MICA-129 genotype frequencies	
Val/Val, *n* (%)	224 (49.6)
Met/Val, *n* (%)	156 (34.5)
Met/Met, *n* (%)	63 (13.9)
ND, *n* (%)	9 (2.0)
MICA-129 allele frequencies	
Val, *n* (%)	604 (68.2)
Met, *n* (%)	282 (31.8)
**Transplantation**	
Source of stem cells	
Peripheral blood, *n* (%)	441 (97.6)
Bone marrow, *n* (%)	11 (2.4)
Busulfan-based conditioning, *n* (%)	402 (88.9)
Total body irradiation-based conditioning, *n* (%)	47 (10.4)
Reduced intensity conditioning, *n* (%)	170 (37.6)
T-cell depletion, *n* (%)	252 (55.8)
**Outcome**	
Occurrence of acute GVHD, *n* (%)	246 (54.4)
Grade I to II, *n* (%)	159 (35.2)
Grade III to IV, *n* (%)	87 (19.2)
Occurrence of chronic GVHD, *n* (%)	138 (30.5)
Occurrence of relapse, *n* (%)	86 (19.0)
Mortality, *n* (%)	178 (39.4)
Treatment-related mortality (TRM), *n* (%)	109 (24.1)
Mortality due to acute GVHD, *n* (%)	52 (11.5)
Infection and other TRM, *n* (%)	57 (12.6)
Mortality due to relapse, *n* (%)	57 (12.6)
Unknown reason of mortality, *n* (%)	12 (2.7)

a ND, missing data or not determined parameters.

Patients carrying a *MICA-129Met* allele had an increased probability of overall survival (hazard ratio [HR] = 0.77 per allele, *P *=* *0.0445), and *MICA-129Met* homozygote patients had a trend toward a lower TRM (odds ratio [OR] = 0.51, *P *=* *0.0907; Table2). Specifically, the mortality due to aGVHD was reduced (OR = 0.57 per allele, *P *=* *0.0400) despite an increased risk for *MICA-129Met* homozygous carriers to experience this complication (OR = 1.92, *P *=* *0.0371). These homozygous carriers showed also a trend toward a lower severity of aGVHD (OR = 0.55, *P *=* *0.1570), which might explain this finding. Notably, having a < 8/8 HLA-matched unrelated donor (*n* = 68, 15.0%) did not significantly affect these outcomes, in contrast to the *MICA* genotype.

**Table 2:** Association of the *MICA-129* genotype with the outcome of HSCT

Cohort *n* = 452	Effect of *MICA-129Met*^a^				Adjusted covariates^c^
	HR/OR	95%-CI	*P*-value	Risk model^b^	
Overall survival	**0.77**	[0.60, 0.99]	**0.0445**	additive	MUD, TCD, diagnosis
Treatment-related mortality	0.51	[0.22, 1.07]	0.0907	recessive	MUD, TCD, diagnosis
Mortality due to aGVHD	**0.57**	[0.32, 0.95]	**0.0400**	additive	MUD, TCD, diagnosis
Mortality due to relapse	1.64	[0.71, 4.01]	0.2607	additive	MUD, TCD, FtoM, diagnosis
Occurrence of aGVHD	**1.92**	[1.05, 3.63]	**0.0371**	recessive	MUD, TCD, diagnosis
Severity of aGVHD^d^	0.55	[0.23, 1.21]	0.1570	recessive	MUD, TCD, diagnosis
Occurrence of cGVHD	1.30	[0.96, 1.77]	0.0884	additive	MUD, TCD, TBI, diagnosis
Occurrence of relapse	0.87	[0.61, 1.23]	0.4313	additive	MUD, TBI, diagnosis

w/o T-cell depletion *n* = 197^e^	Effect of *MICA-129Met*^a^				Effect of no T-cell depletion^f^	
	HR/OR	95%-CI	*P*-value	Risk model^b^	HR/OR	*P*-value
Overall survival	**0.67**	[0.46, 0.98]	**0.0382**	additive	**1.59**	**0.0165**
Treatment-related mortality	0.35	[0.08,1.17]	0.1220	recessive	1.60	0.0991
Mortality due to aGVHD	**0.44**	[0.18, 0.92]	**0.0420**	additive	2.16	0.0559
Mortality due to relapse	3.43	[0.82, 26.1]	0.1450	additive	**4.92**	**0.0285**
Occurrence of aGVHD	1.54	[0.59, 4.53]	0.4037	recessive	**2.16**	**0.0011**
Severity of aGVHD^d^	0.30	[0.06, 1.02]	0.0768	recessive	1.57	0.1720
Occurrence of cGVHD	1.18	[0.76, 1.81]	0.4600	additive	**2.59**	**0.0001**
Occurrence of relapse	0.65	[0.37, 1.11]	0.1257	additive	1.01	0.9670

T-cell depletion *n* = 252^e^	Effect of *MICA-129Met*^a^				Effect of T-cell depletion^f^	
	HR/OR	95%-CI	*P*-value	Risk model^b^	HR/OR	*P*-value
Overall survival	0.88	[0.62, 1.25]	0.4821	additive	**0.63**	**0.0165**
Treatment-related mortality	0.65	[0.22, 1.61]	0.3799	recessive	0.62	0.0991
Mortality due to aGVHD	0.71	[0.31, 1.50]	0.3941	additive	0.46	0.0559
Mortality due to relapse	1.68	[0.45, 7.60]	0.4565	additive	**0.20**	**0.0285**
Occurrence of aGVHD	**2.46**	[1.13, 5.66]	**0.0271**	recessive	**0.46**	**0.0011**
Severity of aGVHD^d^	0.89	[0.29, 2.52]	0.8365	recessive	0.64	0.1720
Occurrence of cGVHD	1.44	[0.92, 2.25]	0.1039	additive	**0.39**	**0.0001**
Occurrence of relapse	1.14	[0.71, 1.81]	0.5720	additive	0.99	0.9670

a Significant effects (HR/OR) with *P*-values ≤ 0.05 are highlighted in bold.

b For each outcome, statistics are given for the most powerful genetic model. A recessive *MICA-129* effect was quantified for the *Met/Met* genotype compared to the pooled *Val/Met* and *Val/Val* genotypes. An additive *MICA-129* effect was quantified per *Met* allele.

c In addition, all analyses were adjusted for a binary indicator distinguishing whether patient and donor had the same *MICA-129* genotype or not. The following abbreviations for covariates are used: FtoM, female donor for male recipient; MUD, HLA-matched unrelated donor; TBI, total body irradiation; and TCD, T-cell-depleting treatment. Analyses for the strata not receiving (w/o T-cell depletion) and receiving ATG (T-cell depletion) were adjusted for the same covariates except for TCD.

d To analyze severity of aGVHD, grades I and II were compared versus grades III and IV.

e Three patients were omitted in the stratified analyses because of missing information on T-cell depletion.

f Effects of applying or not applying a T-cell-depleting treatment with ATG were analyzed irrespective of the MICA genotype with adjustment for the relevant covariates to confirm the expected effects of applying or omitting ATG treatment on outcome in the cohort. The HR/OR in these strata are reciprocal values having the same *P*-value.

**Figure 1:**
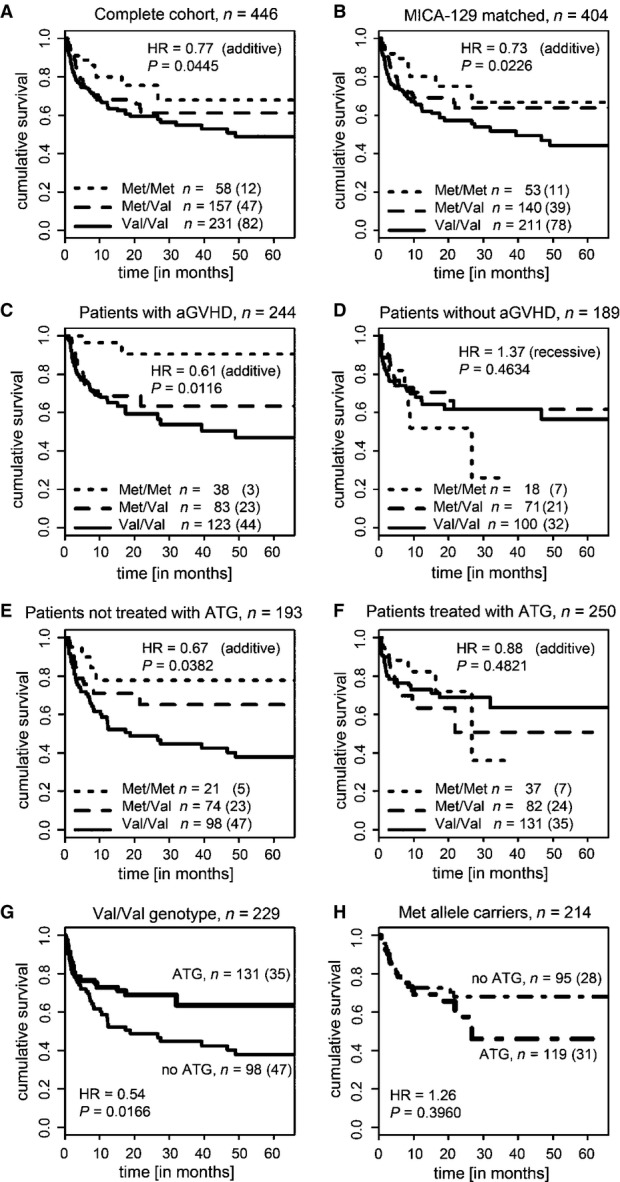
Cumulative survival according to *MICA-129* genotype Kaplan–Meier survival curves stratified by the patient *MICA-129* genotype for all patients (*n *=* *446). The survival curve is displayed for the first 66 months; 7.6% of the patients were followed longer. Effects on overall survival were determined by Cox regression with covariate adjustment as indicated in Table2. The HR indicates the risk per *MICA-129Met* allele carried by the patients (additive risk model). The numbers of patients carrying the three genotypes and the number of events (in brackets) are indicated.

Kaplan–Meyer curves stratified for the *MICA-129* genotypes are shown in Fig1A for the complete cohort. An improved overall survival was observed similarly in the subgroup transplanted with *MICA-129*-matched grafts (HR = 0.73 per allele, *P *=* *0.0226), showing that the effects were associated with the *MICA-129* variants itself and not caused by a *MICA-129* mismatch (Fig1B). The beneficial effect of the *MICA-129Met* allele on overall survival was present in patients experiencing aGVHD (HR = 0.61 per allele, *P *=* *0.0116; Fig1C) but not in patients without aGVHD (HR 1.37, *P *=* *0.4634; Fig1D), suggesting that the SNP modulates the risk of fatal aGVHD. The beneficial effect was also present in patients who did not receive a T-cell-depleting treatment (HR = 0.67 per allele, *P *=* *0.0382; Fig1E), in contrast to those receiving anti-thymocyte globulin (ATG; HR = 0.88, *P *=* *0.4821; Fig1F), suggesting an effect on T-cell function. Particularly patients carrying a *MICA-129Val/Val* genotype appeared to profit from T-cell depletion when overall survival was compared with *MICA-129Val/Val* carriers not treated with ATG (HR = 0.54, *P *=* *0.0166; Fig1G). No advantage of treatment with ATG was seen in patients carrying one or two *MICA-129Met* alleles (HR = 1.26, *P* = 0.3960; Fig1H).

Kaplan–Meier survival curves for patients receiving a graft matched for the *MICA-129* genotype (*n *=* *404).

Kaplan–Meier survival curves for patients who experienced aGVDH (any grade, *n *=* *244).

Kaplan–Meier survival curves for patients not experiencing aGVHD (*n *=* *189). The HR indicates the risk of patients carrying two *MICA-129Met* alleles (recessive risk model).

Kaplan–Meier survival curves for patients who did not receive a T-cell-depleting treatment with ATG (*n *=* *193).

Kaplan–Meier survival curves for patients treated with ATG (*n *=* *250).

Kaplan–Meier survival curves for patients with the *MICA-129Val/Val* genotype (*n *=* *229) stratified by treatment with ATG.

Kaplan–Meier survival curves for patients carrying one or two *MICA-129Met* alleles (*n *=* *214) stratified by treatment with ATG.

In patients not treated with ATG, the beneficial effect of carrying a *MICA-129Met* allele on overall survival (HR = 0.67 per allele, *P *=* *0.0382) and mortality due to aGVHD (OR = 0.44 per allele, *P *=* *0.0420) even canceled out the unfavorable effects of not giving ATG on overall survival (HR = 1.59, *P *=* *0.0165; Table2). In this patient subgroup, an even clearer trend toward a lower severity of aGVHD (OR = 0.30, *P *=* *0.0768) was observed than in all patients. On the other side, *MICA-129Met* alleles might increase the risk of death due to relapse although not at a significant level in this patient subgroup (OR = 3.43 per allele, *P *=* *0.1450). In patients receiving ATG, the association of the *MICA-129Met/Met* genotype with an increased risk of aGVHD (OR = 2.46, *P *=* *0.0271) was more prominent than in all patients despite an overall lower risk of occurrence of aGVHD due to T-cell depletion (OR = 0.46, *P *=* *0.0011; Table2).

In summary, *MICA-129Met* alleles appeared to confer a higher risk of aGVHD albeit with beneficial effects on survival after HSCT. Specifically, the risk to die due to aGVHD was reduced in patients carrying a *MICA-129Met* allele, whereas patients carrying a *MICA-129Val/Val* genotype profited particularly from ATG treatment. To address the immunological mechanisms involved in these partially counterintuitive outcomes, we investigated whether the MICA-129 variants differ in their ability to trigger NKG2D signals after binding.

### Experimental tools used to study functional effects of the MICA-129Val/Met dimorphism

**Figure 2:**
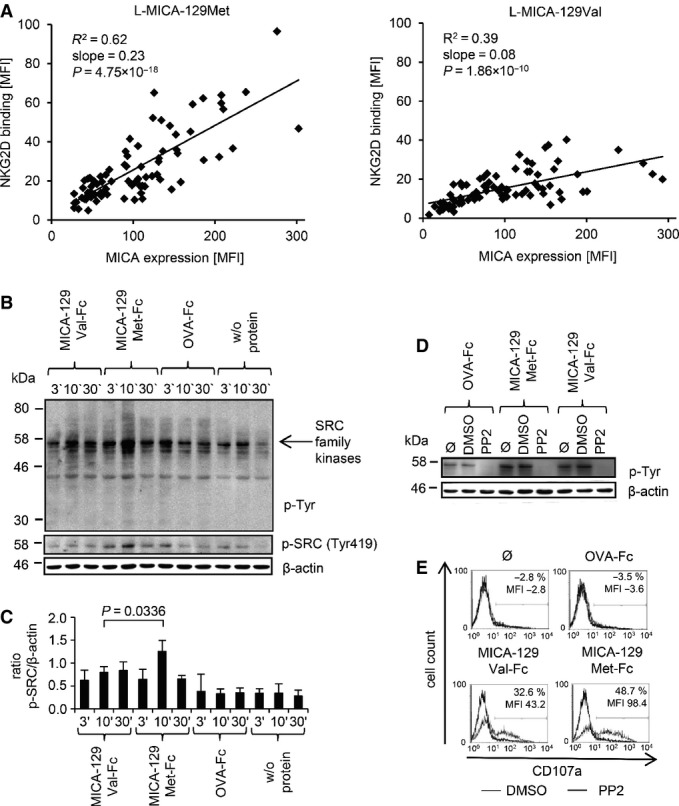
NKG2D binding to the MICA-129Met and MICA-129Val isoform and triggering of phosphorylation of SRC family kinases The linear regression of MICA expression intensity and binding of a recombinant NKG2D-Fc fusion protein both determined as MFI by flow cytometry is displayed for L-MICA-129Met (*n *=* *79, left panel) and L-MICA-129Val clones (*n *=* *81, right panel). The coefficients of determination (*R*^2^), the regression coefficients (reg. coeff.), and the *P*-values for Pearson correlation are indicated.

We generated two sets of tools to analyze the functional effects of the MICA-129Val/Met dimorphism. First, we stably transfected L cells, which like all mouse cells do not possess a *MICA* gene, with expression constructs encoding a MICA-129Met or MICA-129Val variant. The MICA-129Met variant was the MICA*00701 allele, which has a methionine at amino acid position 129. In the MICA-129Val construct, the amino acid position 129 was changed to valine. The resulting L-MICA-129Met and L-MICA-129Val cells, in contrast to vector-only transfected L-con cells, expressed MICA and bound a human NKG2D-Fc protein (Appendix Fig S1A). A broad range of MICA expression intensities was observed on different clones, but on average, these intensities were similar for both variants (Appendix Fig S1B). Analysis of the ratio of MICA expression and NKG2D binding revealed clearly a higher avidity of the MICA-129Met than MICA-129Val variant for NKG2D (Appendix Fig S1C and D) in accord with previous results (Steinle *et al*, 2001). Notably, binding of NKG2D to the MICA-129Met isoform was more dependent on the intensity of MICA expression on individual clones (coefficient of determination *R*^2^ = 0.62) than to the MICA-129Val isoform (*R*^2^ = 0.39). The slope (regression coefficient) of NKG2D binding with increasing MICA expression intensity was steeper for the MICA-129Met (0.23; Fig2A, left panel) than for the MICA-129Val variant (0.08; Fig2A, right panel).

Purified IL-2-stimulated (100 U/ml for 4 days) NK cells (10^6^) were stimulated with immobilized MICA-129Met-mIgG_2a_-Fc or MICA-129Val-mIgG_2a_-Fc or OVA-mIgG_2a_-Fc fusion proteins (10 μg/ml) for 3, 10, or 30 min. The protein lysates of these cells were separated by SDS–PAGE, and the blot was probed subsequently with an anti-phospho-Tyr mAb, an anti-phospho-SRC family (Tyr419) kinases Ab, and an anti-β-actin mAb as a loading control. The arrow points toward phosphorylated SRC family kinases.

Blots obtained from three independent experiments were analyzed by densitometry, and the means plus SD of the ratio between phospho-SRC family kinase and β-actin signals is displayed. The difference between NK cells stimulated for 10 min by MICA-129Met-Fc or MICA-129Val-Fc proteins was assessed by *t*-test.

Purified IL-2-stimulated NK cells (100 U/ml for 4 days, 10^6^) were incubated with the SRC kinase inhibitor PP2 (25 μM), the vehicle DMSO, or medium only (Ø) for 30 min before being added to immobilized MICA-129Met-Fc, MICA-129Val-Fc, or OVA-Fc fusion proteins (10 μg/ml) for 10 min. The protein lysates of these cells were separated by SDS–PAGE, and the blot was probed subsequently with an anti-phospho-Tyr mAb and an anti-β-actin mAb as a loading control. The blot is representative for two independent experiments.

In parallel, degranulation of the NK cells was measured by anti-CD107a staining in flow cytometry. The difference between DMSO- and PP2-treated cells with respect to CD107a^+^ cells and the MFI of CD107a is indicated in the histograms. The results are representative for two independent experiments.

Second, we produced for further examination of the MICA-NKG2D interaction both MICA variants and, as control, ovalbumin (OVA) as mouse-IgG_2a_-Fc fusion proteins (Appendix Fig S2A and B). Both MICA-Fc proteins, in contrast to the OVA-Fc protein, bound specifically and concentration-dependent (*P *=* *4.28 × 10^−9^, analysis of variance (ANOVA)) to NK cells (Appendix Fig S2C and D). However, there was no significant difference between the MICA-129Met-Fc and MICA-129Val-Fc proteins when the mean fluorescence intensity (MFI; *P *=* *0.2833) or the percentage of MICA-binding NK cells (*P *=* *0.2050) was evaluated (two-way analysis of covariance (ANCOVA) adjusted for protein concentration). No significant difference was seen in binding of the two MICA-Fc fusion proteins to NKG2D in surface plasmon resonance (SPR) analysis (Appendix Fig S3). Nonetheless, these proteins differed in their ability to elicit NKG2D signals (see below).

Engagement of NKG2D alone is not sufficient to induce the release of cytotoxic granules from resting NK cells (Bryceson *et al*, 2009), and freshly isolated NK cells indeed failed to kill the L-MICA-129Met or L-MICA-129Val cell lines in contrast to K562 target cells (Appendix Fig S4A and B). Moreover, no degranulation (CD107a expression) or interferon (IFN)-γ release was elicited (Appendix Fig S4C and D). Exposure to MICA-expressing targets also failed to induce the release of TNF-α, IL-10, and IL-13 from NK cells at least within the first 4 h of co-culture (Appendix Fig S4E). However, we have shown previously that IL-2-stimulated NK cells readily kill MICA-expressing L cells (Elsner *et al*, 2010). Therefore, we used IL-2-stimulated NK cells in subsequent experiments. A comparison of freshly isolated and IL-2-stimulated (100 U/ml for 4 days) NK cells is shown in FigEV1. The NKG2D expression intensity was not significantly altered by IL-2 on the major NK-cell subpopulations (CD56^dim^CD16^+^ and CD56^bright^CD16^−^) and the intermediate population (CD56^bright^CD16^+^), but the frequency of CD56^dim^CD16^+^ cells was reduced in contrast to the other two populations.

**Figure EV1:**
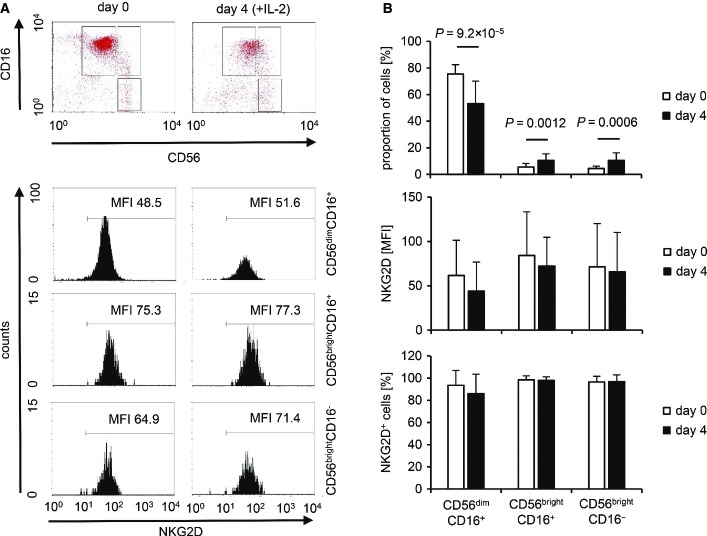
NKG2D expression on freshly isolated and IL-2-stimulated NK-cell populations Three populations (CD56^dim^CD16^+^, CD56^bright^CD16^+^, CD56^bright^CD16^−^) were gated on freshly isolated NK cells (day 0) and NK cells stimulated for 4 days with IL-2 (100 U/ml) as exemplified in the upper panel. The NKG2 D expression on these NK-cell populations is displayed in the lower panel, and the MFI for NKG2D is indicated.

Summaries (means + SD) of the proportion of the three NK-cell populations and their NKG2D expression intensity (MFI and percentage NKG2D^+^ cells) at day 0 (*n *=* *21) and 4 days after IL-2 stimulation (*n *=* *16) are shown. The data were analyzed by *t*-test, and the *P*-values of significant differences are indicated.

### The MICA-129Met variant triggers stronger phosphorylation of SRC kinases in NK cells than the MICA-129Val variant

NKG2D signaling has been characterized best in NK cells (Billadeau *et al*, 2003; Andre *et al*, 2004). Therefore, we stimulated human NK cells for 3, 10, and 30 min with immobilized MICA-129Met-Fc and MICA-129Val-Fc proteins to determine differences in NKG2D-mediated signaling. Western blot analysis using anti-phospho-tyrosine and anti-phospho-SRC family kinases (Tyr419) antibodies (Ab), respectively, showed a stronger phosphorylation of SRC kinases triggered by the MICA-129Met compared to the MICA-129Val variant (Fig2B). The ratio of phosphorylated SRC kinase and β-actin signals was determined in three independent experiments and revealed a significant difference 10 min after stimulation (*P* = 0.0336, *t*-test; Fig2C). Inhibition of SRC family kinases by PP2 completely abolished MICA-129Met-Fc- and MICA-129Val-Fc-triggered degranulation (Fig2D and E), and lysis of L-MICA-129Met and L-MICA-129Val target cells (Appendix Fig S5A), as well as MICA-129Met-Fc- and MICA-129Val-Fc-triggered IFNγ and TNF-α release (Appendix Fig S5B). In these assays, the inhibitor PP2 did not induce apoptosis of NK cells (Appendix Fig S5C and D). The augmented SRC kinase phosphorylation induced by the MICA-129Met variant could therefore be relevant for the extent of NK-cell activation.

### The MICA-129Met variant is overall a stronger trigger of NK-cell cytotoxicity than the MICA-129Val variant, but the MICA-129Val variant is outperforming at high MICA expression intensities on target cells

**Figure 3:**
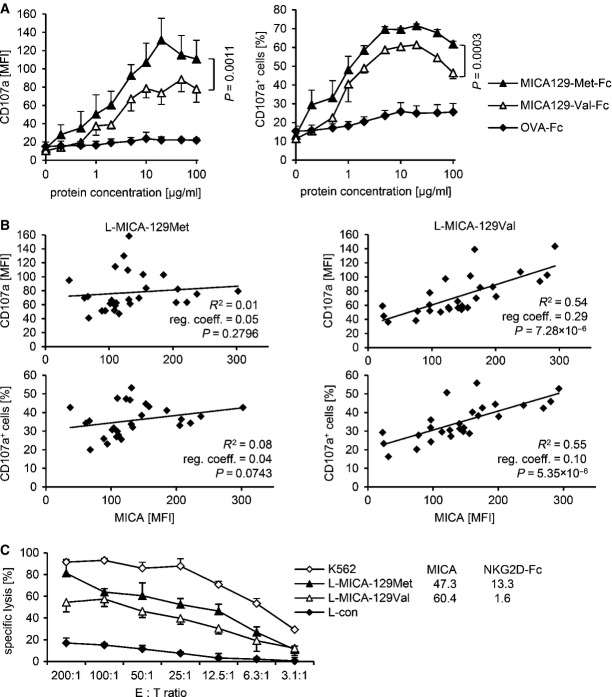
Cytotoxic activity of NK cells in response to the MICA-129Met and MICA-129Val isoforms The degranulation (CD107a expression) of purified IL-2-stimulated (100 U/ml for 4 days) NK cells in response to immobilized MICA-129Met-Fc and MICA-129Val-Fc, and as negative control, OVA-Fc fusion proteins was determined by flow cytometry after 1 h (*n *=* *3). Displayed are means and SD of the MFI (left panel) and the percentage of CD107a^+^ cells (right panel). Differences between MICA-129Met-Fc- and MICA-129Val-Fc-induced NK-cell degranulation were analyzed by two-way ANCOVA adjusted for MICA protein concentration, and the respective *P*-values are indicated.

**Figure EV2:**
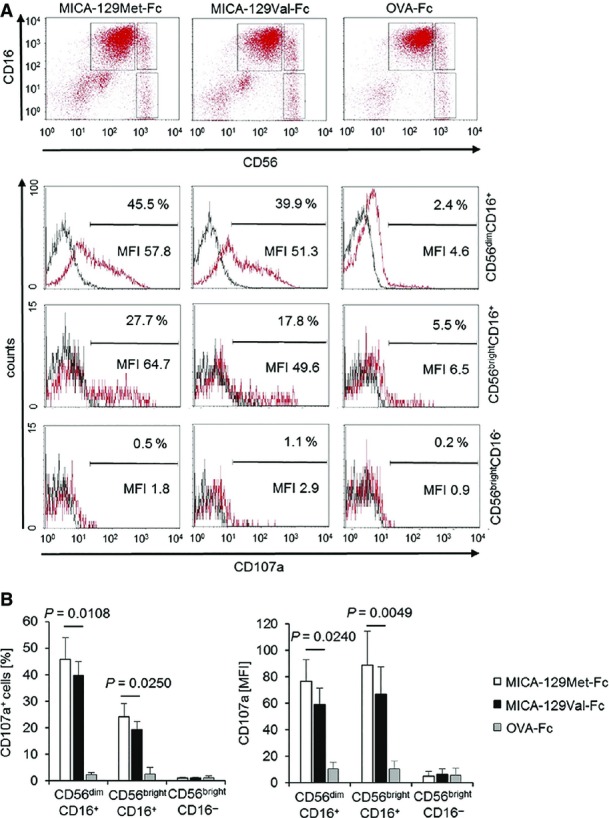
Degranulation of CD56^dim^CD16^+^ and CD56^bright^CD16^+^ NK cells in response to the MICA-129Met and the MICA-129Val isoforms Purified IL-2-stimulated NK cells (100 U/ml for 4 days) were cultured for 2 h on plates coated with MICA-129Met-Fc, MICA-129Val-Fc, or OVA-Fc proteins (10 μg/ml) before flow cytometry. The NK-cell populations were gated as illustrated in the upper panel (CD56^dim^CD16^+^, CD56^bright^CD16^+^, CD56^bright^CD16^−^), and CD107a expression was determined as displayed in the lower panel (red: CD107a, black: isotype control). The specific MFI (MFI CD107a minus MFI isotype control) and the percentage of CD107a^+^ cells are indicated.

The MICA-129Met-Fc protein indeed elicited significantly more NK-cell degranulation than the MICA-129Val-Fc protein (Fig3A) based on the MFI of the degranulation marker CD107a (*P *=* *0.0011) and the percentage of CD107a^+^ NK cells (*P *=* *0.0003; two-way ANCOVA adjusted for MICA protein concentration). CD56^dim^CD16^+^ and, to a lesser extent, CD16^bright^CD16^+^ NK cells responded to NKG2D engagement by degranulation in contrast to CD56^bright^CD16^−^ NK cells (Fig EV2A). The MICA-129Met-Fc variant elicited in both CD16^+^ NK-cell populations significantly more degranulation than the MICA-129Val-Fc protein as indicated by the proportion of CD107a^+^ cells (*P *=* *0.0108 and *P *=* *0.0240, *t*-test) and the MFI of CD107a (*P *=* *0.0250 and *P *=* *0.0049, *t*-test; FigEV2B).

The degranulation of IL-2-stimulated LAK cells (100 U/ml for 4 days) exposed to L-MICA-129Met (*n *=* *27) or L-MICA-129Val clones (*n *=* *27) for 1 h was determined by flow cytometry. CD107a cell surface expression was analyzed after gating on CD56^+^ NK cells. In parallel, the MICA expression on target cells was determined. Displayed are the linear regressions of the MFI of CD107a on NK cells (upper panels) or the proportion of CD107a^+^ NK cells (lower panels) and the MICA expression intensity on target cells (MFI) for the L-MICA-129Met (left panels) and L-MICA-129Val clones (right panels). The coefficients of determination (*R*^2^), the regression coefficients (reg. coeff.), and the *P*-values for Pearson correlation are indicated.

A representative of 21 experiments is shown demonstrating the specific cytotoxic activity of LAK cells against an L-MICA-129Met and an L-MICA-129Val clone. L-con cells served as a negative and K562 cells as a positive control. The means of specific lysis of triplicates plus SD at different E:T ratios (200:1 to 3:1) were measured in an ^51^chromium-release assay. The MICA expression intensity and the binding of a recombinant NKG2D-Fc fusion protein to the target cells were determined in parallel by flow cytometry, and the MFIs are indicated.

A summary (means + SD) of 5 experiments is shown. The data were analyzed by *t*-test, and the *P*-values of significant differences are indicated.

Next, we measured the CD107a expression on NK cells exposed to L-con, L-MICA-129Met, or L-MICA-129Val clones (Appendix Fig S6). After adjustment for MICA expression intensity on different clones, the MFI of CD107a was 38.5 units lower on NK cells exposed to L-MICA-129Val targets compared to NK cells attacking MICA-129Met clones (*P *=* *0.0174, ANCOVA) and 10.1%-points less NK cells were CD107a^+^ (*P *=* *0.0456, ANCOVA). Notably, for NK cells encountering the MICA-129Val variant, degranulation significantly increased with MICA expression intensity (MFI of CD107a regression coefficient 0.29), in contrast to NK cells exposed to the MICA-129Met variant (MFI of CD107a regression coefficient 0.05; Fig3B).

To establish the relevance of the MICA-129 dimorphism for killing of target cells, we analyzed the susceptibility of L cells expressing the MICA-129 variants to killing by lymphokine-activated killer (LAK) cells as exemplified in Fig3C. For statistical analysis, the killing of K562 cells at an effector to target (E:T) ratio of 200:1 was set to 100% in all individual experiments and used to calculate the relative lysis of the other targets. With adjustment to the MICA expression intensity on different clones, the relative lysis of the MICA-129Val variant expressing L cells was by 13.0%-points reduced compared to L cells expressing the Met variant (at an E:T ratio of 200:1, *n* = 84 clones, *P *=* *0.0044, two-way ANCOVA). Notably, the MICA expression intensity had even a negative influence on killing for targets expressing the MICA-129Met variant (regression coefficient −0.0834, *P *=* *0.0083, two-way ANCOVA). In contrast, killing increased with expression intensity of the MICA-129Val variant (regression coefficient 0.1257, *P *<* *0.0001 for interaction between MICA-129 variant and MICA expression intensity, two-way ANCOVA).

Taken together, the MICA-129Met variant triggered stronger NK-cell cytotoxicity at lower MICA expression intensities compared to the MICA-129Val variant. Degranulation increased with expression intensity of the MICA-129Val variant, whereas high expression of the MICA-129Met variant even decreased target cell killing.

### The MICA-129Met variant is overall a stronger trigger of IFNγ release by NK cells than the MICA-129Val variant, but IFNγ expression decreases at high MICA-129Met expression intensities

IFNγ release is a further effector function of NK cells triggered by NKG2D. CD56^bright^CD16^−^ and, to a lesser extent, CD56^bright^CD16^+^ NK cells responded to the MICA-129Met-Fc and MICA-129Val-Fc proteins with IFNγ expression in contrast to CD56^dim^CD16^+^ NK cells (FigEV3A). The MICA-129Met-Fc protein induced more IFNγ^+^ NK cells than the MICA-129Val-Fc variant (*P *=* *0.0026 and *P *=* *0.0434, ANCOVA adjusted for MICA protein concentration), and the MFI of IFNγ was higher (*P *=* *0.0306 and *P *=* *0.0074; FigEV3B). The MICA-129Met-Fc protein elicited more IFNγ (*P *=* *0.0216, ANCOVA adjusted to protein concentration) and also more TNF-α release from NK cells than the MICA-129Val-Fc variant (*P *=* *0.0363) as determined by enzyme-linked immuno-sorbent assays (ELISA). Release of the Th_2_ cytokines IL-10 and IL-13 was not induced at least not within the first 4 h of stimulation. Engagement of NKG2D appeared even to inhibit IL-13 production (Appendix Fig S8).

**Figure EV3:**
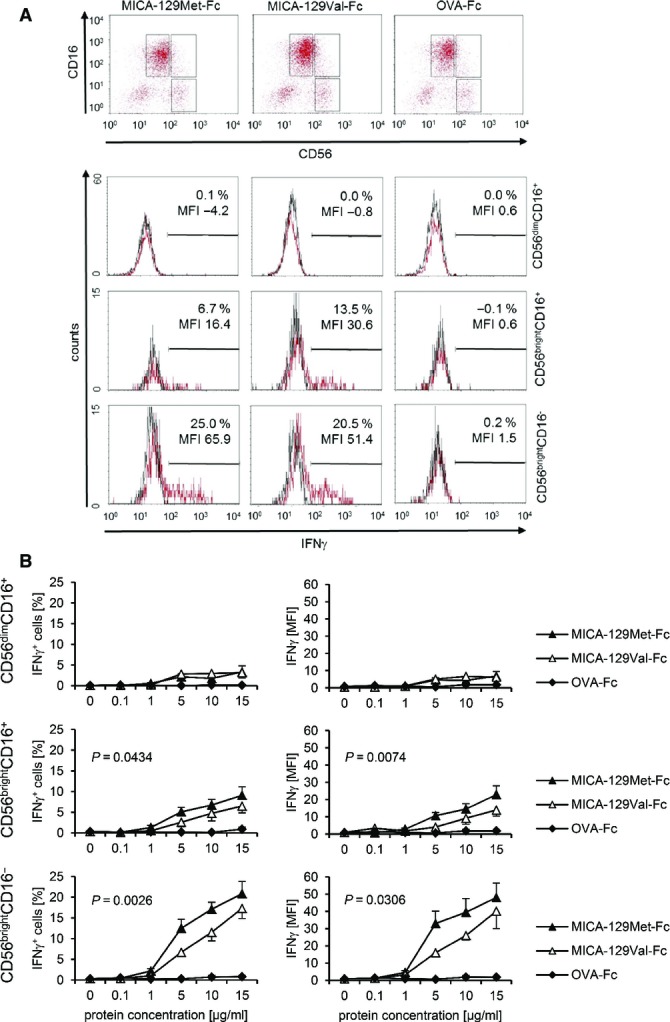
Expression of IFNγ in CD56^bright^CD16^−^ and CD56^bright^CD16^+^ NK cells in response to the MICA-129Met and the MICA-129Val isoforms Purified IL-2-stimulated NK cells (100 U/ml for 4 days) were cultured for 4 h on plates coated with MICA-129Met-Fc, MICA-129Val-Fc, or OVA-Fc proteins (0, 0.1, 1, 5, 10, 15 μg/ml) before flow cytometry. The NK-cell populations were gated as exemplified in the upper panel (CD56^dim^CD16^+^, CD56^bright^CD16^+^, CD56^bright^CD16^−^). The intracellular IFNγ expression in these populations is displayed in the lower panel (red: IFNγ, black: isotype control). The specific MFI (MFI IFNγ minus MFI isotype control) and the percentage of IFNγ^+^ cells are indicated.

A summary (means + SEM) of 9 experiments is shown. The data were analyzed by two-way ANCOVA adjusted for the protein concentration (5, 10, 15 μg/ml), and the *P*-values of significant differences between the MICA-129Met-Fc and MICA-129Val-Fc proteins are indicated.

**Figure 4:**
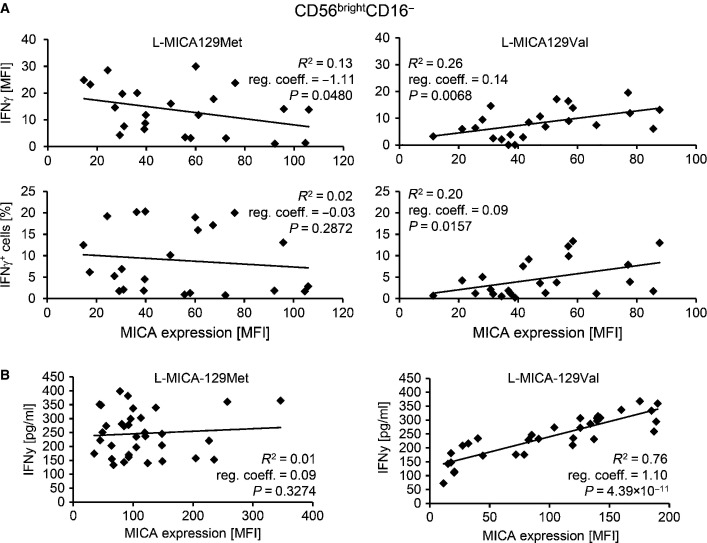
IFNγ production of NK cells in response to the MICA-129Met and MICA-129Val isoforms The IFNγ expression of purified IL-2-stimulated (100 U/ml for 4 days) NK cells exposed for 4 h to L-MICA-129Met (*n *=* *23) or L-MICA-129Val clones (*n *=* *23) was determined by flow cytometry. The IFNγ expression was analyzed after gating on CD56^bright^CD16^−^ NK cells. In parallel, the MICA expression on target cells was determined. Displayed are the linear regressions of the MFI of IFNγ in CD56^bright^CD16^−^ NK cells (upper panels) or the proportion of IFNγ^+^ CD56^bright^CD16^−^ NK cells (lower panels) and the MICA expression intensity on target cells (MFI) for the L-MICA-129Met (left panels) and L-MICA-129Val clones (right panels). The coefficients of determination (*R*^2^), the regression coefficients (reg. coeff.), and the *P*-values for Pearson correlation are indicated.

Next, we exposed NK cells to MICA-expressing L cells. After adjustment to MICA expression intensity on different clones, CD56^bright^CD16^−^ NK cells co-cultured with L-MICA-129Val targets expressed less IFNγ compared to cells exposed to MICA-129Met clones. The MFI of IFNγ was on average 12.5 units lower (*P *=* *0.0032, ANCOVA), and 7.5%-points less CD56^bright^CD16^−^ NK cells were IFNγ positive (*P *=* *0.0061, ANCOVA). For CD56^bright^CD16^−^ NK cells encountering the MICA-129Val variant, the proportion of IFNγ^+^ cells and the MFI of IFNγ increased with MICA expression intensity (regression coefficients 0.14 and 0.09, respectively), whereas cells exposed to the MICA-129Met variant expressed less IFNγ when co-cultured with targets with higher MICA expression intensity (MFI, regression coefficient −1.11; Fig4A). The results obtained for CD56^bright^CD16^+^ and CD56^dim^CD16^+^ NK cells in these experiments are shown in the Appendix Fig S7.

The IFNγ release of purified IL-2-stimulated NK cells (100 U/ml for 4 days) co-cultured with L-MICA-129Met (*n *=* *34) or L-MICA-129Val clones (*n *=* *32) for 24 h was measured in the supernatant by ELISA. In parallel, the MICA expression intensity on target cells was determined by flow cytometry. The linear regressions of IFNγ release (pg/ml) by NK cells and MICA expression on targets (MFI) are displayed for the L-MICA-129Met clones (left panel) and the L-MICA-129Val clones (right panel). The coefficients of determination (*R*^2^), the regression coefficients (reg. coeff.), and the *P*-values for Pearson correlation are indicated.

The effect of the MICA-129 dimorphism was further confirmed when IFNγ release was measured by ELISA in an independent set of experiments. Adjusted to MICA expression intensity on different clones, IFNγ production was by 176.5 pg/ml higher for NK cells exposed for 24 h to L-MICA-129Met compared to L-MICA-129Val clones (*P *<* *0.0001, ANCOVA; Fig4B). For NK cells exposed to the MICA-129Val variant, the MICA expression intensity had a strong effect on IFNγ release (1.103 pg/ml per MFI unit of MICA, *P *<* *0.0001, ANCOVA), in contrast to NK cells exposed to L-MICA-129Met targets (0.142 pg/ml per MFI unit, *P *=* *0.0001 for interaction between MICA-129 variant and MICA expression intensity, ANCOVA). IFNγ production of NK cells cultured in the absence of target cells or secretion of NK cells exposed to the L-con cells was either not detectable or < 25 pg/ml.

### The MICA-129Met variant leads to stronger down-regulation of NKG2D on the plasma membrane of NK cells than the MICA-129Val variant

Next, we asked why increasing expression of the high-avidity MICA-129Met isoform did not trigger continuously stronger functional responses as the low-avidity MICA-129Val isoform did. Since MICA can be cleaved from the cell surface (Groh *et al*, 2002; Salih *et al*, 2002; Waldhauer *et al*, 2008), one possibility was that higher expression of MICA leads to higher concentrations of soluble MICA (sMICA), which can inhibit NKG2D signaling (Groh *et al*, 2002). However, L-MICA-129Met and L-MICA-129Val clones did not release MICA (Appendix Table S1).

**Figure 5:**
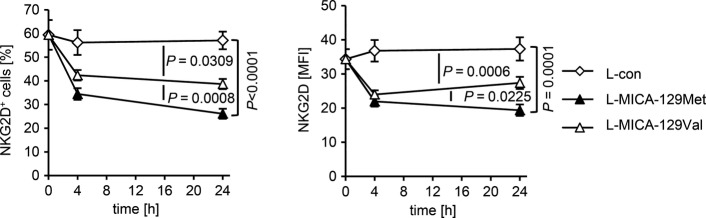
Down-regulation of NKG2D on NK cells in response to the MICA-129Met and MICA-129Val isoforms NKG2D expression on purified IL-2-stimulated NK cells (100 U/ml for 4 days) exposed to L-MICA-129Met (*n *=* *25) or L-MICA-129Val clones (*n *=* *25) for 0, 4, and 24 h was analyzed by flow cytometry. NK cells (2.5 × 10^5^) were co-cultured with 5 × 10^4^ target cells and analyzed for NKG2D expression after gating of CD3^−^CD56^+^ cells. The means and SD of the percentage of NKG2D^+^ NK cells (left panel) and of the MFI of NKG2D (right panel) are shown. Differences between the groups were analyzed by repeated measures ANOVA, and *P*-values for pairwise comparisons are indicated.

Sustained exposure of NK cells to NKG2D ligand-expressing cells can also down-regulate NKG2D (Coudert *et al*, 2005; Ogasawara *et al*, 2005; Oppenheim *et al*, 2005; Wiemann *et al*, 2005). Thus, we investigated NKG2D expression on NK cells exposed for 4 and 24 h to L-con, L-MICA-129Met, or L-MICA-129Val clones (Fig5, Appendix Fig S9A). The percentage of NKG2D^+^ NK cells decreased by 9.5%-points for the MICA-129Val (*P *=* *0.0309) and by 19.4%-points for the MICA-129Met variant (*P *<* *0.0001) compared to the control (co-culture with L-con cells; two-way ANCOVA adjusted for MICA expression intensity on different clones). The MFI of NKG2D decreased by 11.8 units for the MICA-129Val (*P *=* *0.0006) and by 13.7 units for the MICA-129Met variant (*P *=* *0.0001) compared to the control (two-way ANCOVA). Notably, the percentage of NKG2D^+^ cells decreased more among NK cells encountering L-MICA-129Met than L-MICA-129Val targets (−9.3%-points, *P *=* *0.0008, two-way ANCOVA). In addition, the MFI of NKG2D was significantly different between NK cells exposed to L-MICA-129Met and L-MICA-129Val targets (*P *=* *0.0225, two-way ANCOVA). In these analyses as in the previous analyses, we adjusted for the MICA expression intensity on different clones although it had little effect on the NKG2D down-regulation on NK cells (Appendix Fig S10). NK cells co-cultured with L-MICA cells did not show differences in CD94 expression indicating the specificity of the effect (Appendix Fig S9B and C).

Notably, the NKG2D expression was significantly reduced on all three NK-cell subpopulations 4 and 24 h after exposure to MICA-expressing targets (FigEV4A). After adjustment to MICA expression intensities, the NKG2D expression was on average 19.5 (*P *=* *8.04 × 10^−6^, CD56^dim^CD16^+^), 16.2 (*P* = 2.26 × 10^−8^, CD56^bright^CD16^+^), or 15.8%-points (*P *=* *1.02 × 10^−4^, CD56^bright^CD16^−^) lower on NK cells exposed to L-MICA-129Met than L-MICA-129Val clones (FigEV4B). Hence, the MICA-129Met variant induced a stronger down-regulation of NKG2D than the MICA-129Val variant. This counter-regulation appears to limit the initially stronger functional effects of the MICA-129Met variant on NKG2D signaling.

**Figure EV4:**
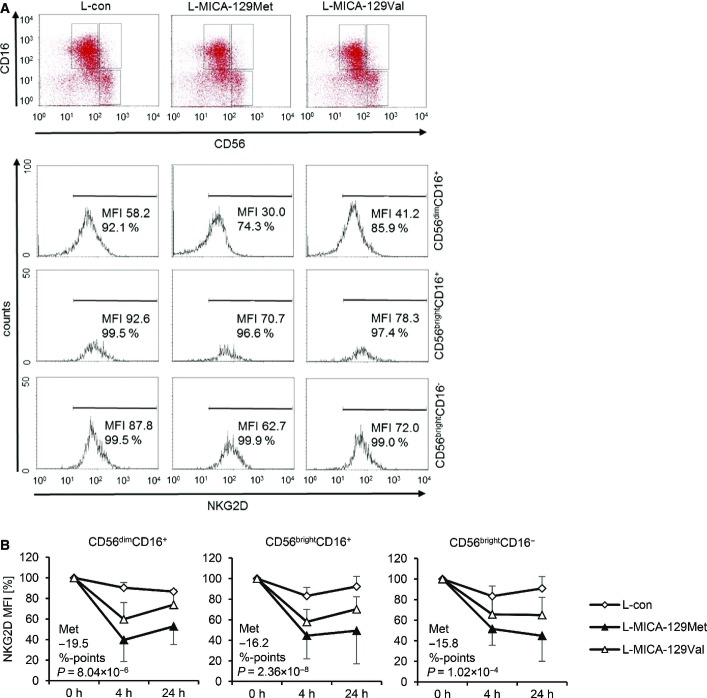
Down-regulation of NGK2D on CD56^dim^CD16^+^, CD56^bright^CD16^+^, and CD56^bright^CD16^−^ NK cells in response to the MICA-129Met and the MICA-129Val isoforms Purified IL-2-stiumlated NK cells (100 U/ml for 4 days) were co-cultured with L-con, L-MICA-129Met, or L-MICA-129Val clones for 4 h. Three NK-cell populations (CD56^dim^CD16^+^, CD56^bright^CD16^+^, CD56^bright^CD16^−^) were gated as illustrated in the upper panel. The NKG2D expression on these NK-cell populations is displayed in the lower panel, and the MFI of NKG2D and the percentages of NKG2D^+^ cells are indicated.

A summary (means + SD) of NKG2D expression on the three NK-cell populations 4 and 24 h after co-culture with L-con (*n *=* *3), L-MICA-129Met (*n *=* *17), and L-MICA-129Val clones (*n *=* *18) is displayed. The NKG2D expression (MFI) at the beginning (0 h) was set to 100%. The average reduction of NKG2D on NK cells in response to L-MICA-129Met compared to L-MICA-129Val clones at 4 and 24 h (%-points) is indicated in the panels. The differences were analyzed by repeated measures ANOVA, and *P*-values are indicated.

### The MICA-129Met variant leads to an earlier antigen-specific activation of CD8^+^ T cells

**Figure 6:**
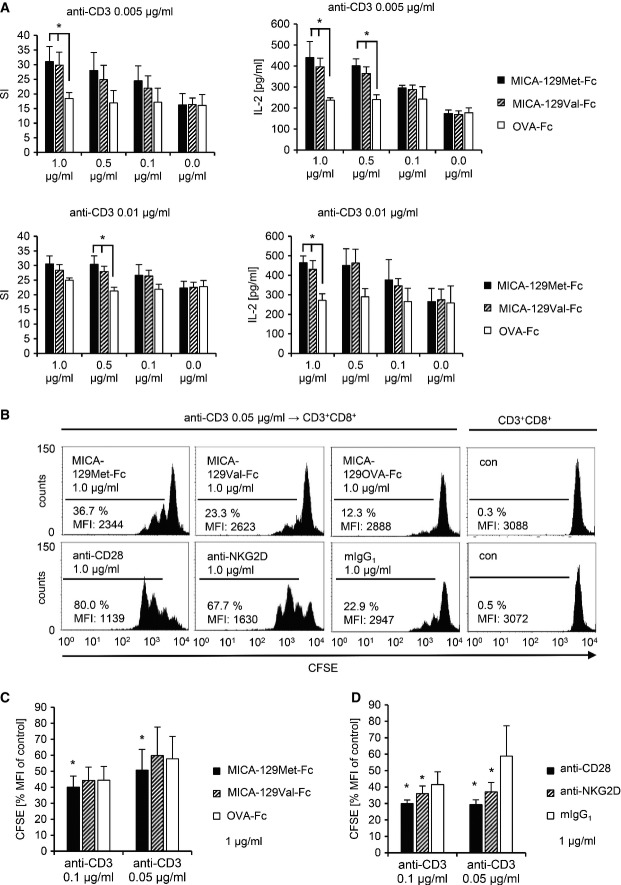
Co-stimulation of CD8^+^ T cells by the MICA-129Met and MICA-129Val isoforms MACS-separated CD8^+^ T cells were cultured in triplicate on an immobilized anti-CD3 mAb (0.005 μg/ml [upper panel] or 0.01 μg/ml [lower panel]) in combination with recombinant MICA-129Met-Fc, MICA-129Val-Fc, and OVA-Fc proteins at various concentrations (1.0, 0.5, 0.1, 0.0 μg/ml). After 72 h, 25% of the supernatant was harvested and IL-2 concentrations were measured by ELISA. The harvested medium was replaced by the same volume containing 1 μCi ^3^H-labeled thymidine. After 12 h, the plates were completely harvested and the DNA-bound radioactivity was determined. The means and SD of the stimulation index (SI) are displayed (*n *=* *4). Significant differences between MICA-129Met/Val-Fc and OVA-Fc proteins were found when the antigen-specific signal (anti-CD3) was limited (*P <* *0.05, *t*-test; upper left panel: 1.0 μg/ml: MICA-129Met-Fc versus OVA-Fc *P *=* *0.0372 and MICA-129Val-Fc versus OVA-Fc *P *=* *0.0366; upper right panel: 1.0 μg/ml: MICA-129Met-Fc versus OVA-Fc *P *=* *0.0499 and MICA-129Val-Fc versus OVA-Fc *P *=* *0.0192; 0.5 μg/ml: MICA-129Met-Fc versus OVA-Fc *P *=* *0.0164 and MICA-129Val-Fc versus OVA-Fc *P *=* *0.0357; lower left panel: 0.5 μg/ml: MICA-129Met-Fc versus OVA-Fc *P *=* *0.0287 and MICA-129Val-Fc versus OVA-Fc *P *=* *0.0232; lower right panel: 1.0 μg/ml: MICA-129Met-Fc versus OVA-Fc *P *=* *0.0171 and MICA-129Val-Fc versus OVA-Fc *P *=* *0.0484).

NKG2D on CD8^+^ T cells functions as a co-stimulatory molecule (Groh *et al*, 2001), and stimulation of NKG2D alone was indeed not sufficient to induce a proliferation of purified CD8^+^ T cells even at high concentrations of anti-NKG2D or MICA-129Met/Val-Fc proteins (Appendix Fig S11). At low concentrations of anti-CD3 (0.005 and 0.01 μg/ml), co-stimulation of CD8^+^ T cells by anti-NKG2D induced proliferation and IL-2 production as expected but less efficiently than co-stimulation by anti-CD28 (Appendix Fig S12). Both, the MICA-129Met-Fc and MICA-129Val-Fc proteins provided co-stimulatory signals but did not differ in their capacity to induce proliferation or IL-2 production when measured after 96 h (Fig6A). However, at 72 h after stimulation, CD8^+^ T cells had proliferated more in response to the MICA-129Met-Fc than MICA-129Val-Fc or OVA-Fc proteins (*P *=* *0.0277, Wilcoxon test), suggesting a slightly earlier activation by NKG2D-mediated co-stimulation via the MICA-129Met-Fc protein (Fig6B and C). The effects of co-stimulation by anti-CD28 and anti-NKG2D are shown for comparison (Fig6D).

Purified CFSE-stained CD8^+^ T cells were stimulated by immobilized anti-CD3 (0.005 μg/ml) in combination with recombinant MICA-129Met-Fc, MICA-129Val-Fc, OVA-Fc proteins, or co-stimulatory mAb (anti-CD28, anti-NKG2D) or an isotype control (mIgG_1_). The proliferation of CD3^+^CD8^+^ T cells was assessed at 60 h by flow cytometry. Results of a representative out of 6 experiments are displayed. Untreated CFSE-stained CD8^+^ T cells are included for comparison. The percentage of proliferating cells and MFI for CFSE are indicated.

The MFI of CFSE in unstimulated CD8^+^ T cells (control) was set to 100% in individual experiments (*n *=* *6), and the relative decrease due to proliferation was calculated. Means + SD are shown. Significant differences (*P =* *0.0277, Wilcoxon test) between MICA-129Met-Fc versus MICA-129Val-Fc and OVA-Fc proteins were found at slightly higher anti-CD3 concentrations (0.1 and 0.05 μg/ml) than at later time points (see A).

Anti-CD28 and anti-NKG2D mAb were used in parallel as a positive control, mean + SD are shown, and significant differences (*P =* *0.0277, Wilcoxon test) to the isotype control (mIgG_1_) are indicated (*n *=* *6).

### The MICA-129Met variant leads to stronger down-regulation of NKG2D on CD8^+^ T cells than the MICA-129Val variant impairing subsequent co-stimulation

**Figure 7:**
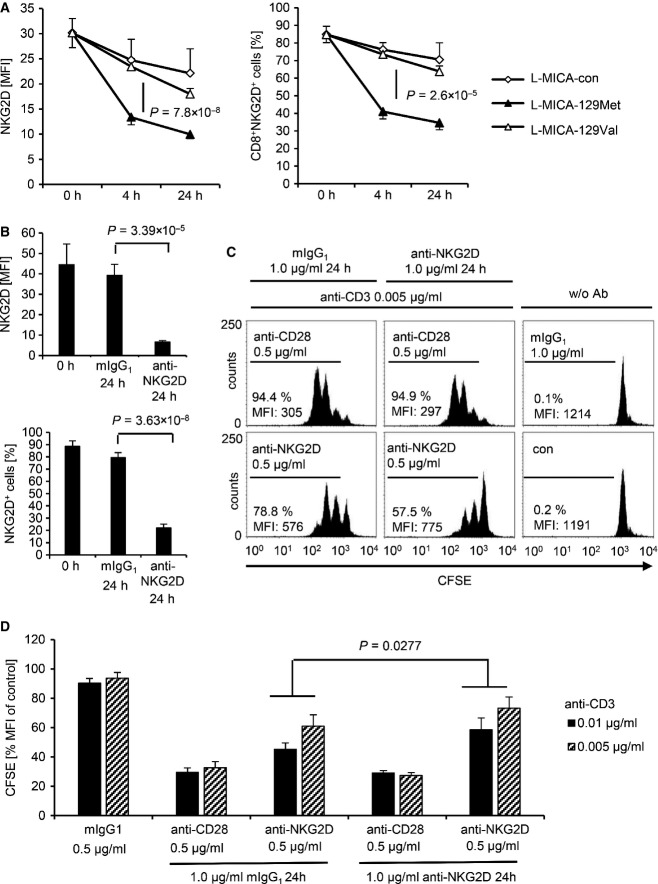
Down-regulation of NKG2D by the MICA-129Met and the MICA-129Val isoforms on CD8^+^ T cells and impairment of subsequent co-stimulation via NKG2D NKG2D expression on purified CD8^+^ T cells exposed to L-MICA-129Met (*n *=* *19) or L-MICA-129Val clones (*n *=* *19) for 0, 4, and 24 h was analyzed by flow cytometry. CD8^+^ T cells (2.5 × 10^5^) were co-cultured with 5 × 10^4^ target cells and analyzed for NKG2D expression after gating on CD3^+^CD8^+^ T cells. The means and SD of the MFI of NKG2D (left panel) and of the percentage of NKG2D^+^CD8^+^ T cells (right panel) are displayed. Differences between the groups were analyzed by repeated measures ANOVA, and the *P*-values are indicated.

NKG2D was strongly down-regulated on CD8^+^ T cells co-cultured with L-MICA-129Met but hardly after co-culture with L-MICA-129Val clones (FigEV5). The proportion of NKG2D^+^CD3^+^CD8^+^ cells decreased by 35.8%-points more when exposed to L-MICA-129Met clones compared to those encountering the L-MICA-129Val clones (*P *=* *7.8 × 10^−8^ two-way ANOVA adjusted for MICA expression intensity on different clones), and the MFI of NKG2D decreased by 12.1 units more (*P *=* *2.6 × 10^−5^; Fig7A). Notably, on CD8^+^ T cells, the MFI of NKG2D decreased clearly more when exposed to clones with higher MICA-129Met expression intensity (*P *=* *0.016, two-way ANCOVA), whereas CD8 expression was not altered indicating the specificity of the effect (Appendix Fig S13). The down-regulation of NGK2D on CD8^+^ T cells was functionally relevant. Exposure of CD8^+^ T cells to anti-NKG2D for 24 h reduced NKG2D expression (Fig7B) and impaired their capability to proliferate subsequently in response to NKG2D-mediated co-stimulation (Fig7C and D and Appendix Fig S14). The strong down-regulation of NKG2D on CD8^+^ T cells by MICA-129Met variants appears to be important for the association of this polymorphism with the outcome of HSCT.

**Figure EV5:**
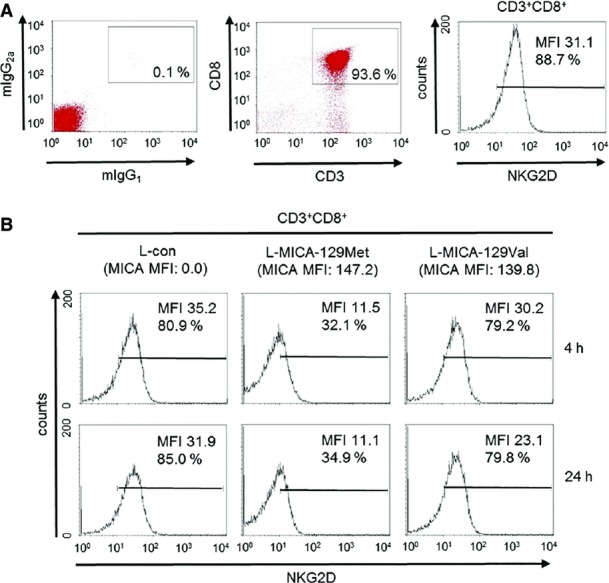
Down-regulation of NGK2D on CD8^+^ T cells in response to the MICA-129Met and MICA-129Val isoforms Purified CD8^+^ T cells were analyzed by flow cytometry for NKG2D expression as illustrated here. The MFI for NKG2D and the percentage of NKG2D^+^CD8^+^ T cells are indicated.

The NK cells were subsequently co-cultured with an L-con, L-MICA-129Met, or L-MICA-129Val clone (the MFI values for MICA on these clones are indicated above the histograms in brackets). NKG2D expression was determined as illustrated in (A) after 4 and 24 h. The MFI for NKG2D and the percentages of NKG2D^+^CD8^+^ T cells are indicated.

Purified CD8^+^ T cells were cultured on plate-bound anti-NKG2D (1 μg/ml) or isotype control (mIgG_1_) for 24 h before the NKG2D expression was measured by flow cytometry. Means and SD of MFI (upper panel) and percentage of NKG2D^+^ cells (lower panel) are shown (*n *=* *6). Differences between the groups were analyzed by *t*-tests, and the *P*-values are indicated.

These CD8^+^ T cells were subsequently CFSE-stained and cultured on plates coated with anti-CD3 (0.005 μg/ml) in combination with anti-CD28 (0.5 μg/ml) as a positive control or anti-NKG2D (0.5 μg/ml). Proliferation was measured after 60 h by flow cytometry. Untreated CFSE-stained CD8^+^ T cells are included for comparison. The percentage of proliferating cells and the MFI for CFSE are indicated.

The MFI of CFSE in unstimulated CD8^+^ T cells (control) was set to 100% in individual experiments (*n *=* *6), and the relative decrease due to proliferation was calculated. Means + SD are shown. Significant differences (Wilcoxon test) between CD8^+^ T cells pre-exposed to anti-NKG2D and isotype control (mIgG_1_) were found in these experiments at anti-CD3 concentrations of 0.01 and 0.005 μg/ml.

## Discussion

Numerous studies have demonstrated the impact of SNPs on the outcome of HSCT (Dickinson, 2008; Harris *et al*, 2013). Those SNPs, which alter gene regulation or protein function, might not only be useful as biomarkers but also identify new targets for therapy. We focused on the MICA-129Val/Met dimorphism, which has been previously associated with the risk of cGVHD and relapse (Boukouaci *et al*, 2009).

We generated MICA-129Met and MICA-129Val variants differing only in amino acid position 129 to enable testing of effects of this single amino acid variation that was reported to distinguish MICA variants with high and low binding avidity to NKG2D (Steinle *et al*, 2001). Natural *MICA* alleles usually combine several polymorphisms, and homozygosity is infrequent, making it impossible to study effects of a single amino acid exchange using patient-derived cells or materials. Using MICA-transfected L cells, we confirmed the previous report by Steinle and colleagues (Steinle *et al*, 2001) that the MICA-129Met variant binds NKG2D better than the MICA-129Val isoform. Replacement of valine by methionine affects NKG2D binding indirectly likely by a conformational change since this position is not directly involved in NKG2D binding (Li *et al*, 1999, 2001). We show here that both MICA isoforms differed also in their efficacy to elicit NKG2D-mediated cellular responses. Notably, the greater efficacy of the MICA-129Met variant to elicit down-stream signals was not explained alone by its higher NKG2D binding avidity. Recombinant MICA-129Met-Fc and MICA-129Val-Fc proteins also elicited different functional responses but did not significantly differ in binding avidity to NKG2D. Thus, both variants varied also in their efficacy to elicit NKG2D signal transduction after binding.

The first alteration downstream of NKG2D binding was a stronger phosphorylation of SRC family kinases in NK cells stimulated by the MICA-129Met compared to the MICA-129Val variant. This included tyrosine residue Y419, which is auto-phosphorylated during activation. SRC kinases are essential for NKG2D-triggered cytotoxicity in human NK cells (Billadeau *et al*, 2003), and the SCR family kinase inhibitor PP2 indeed completely abolished degranulation and IFNγ release induced by the recombinant MICA-129Met/Val proteins. MICA-129Met ligands triggered more degranulation of CD56^dim^CD16^+^ NK cells and IFNγ production of CD56^bright^CD16^−^ NK cells than MICA-129Val ligands. The amount of degranulation and IFNγ secretion correlated positively with the MICA expression intensity on target cells but only for the MICA-129Val variant. In contrast, the expression intensity of MICA-129Met ligands had either none or even a negative effect on the magnitude of degranulation and IFNγ expression or release. It also inversely influenced the killing of target cells, whereas killing increased steadily with expression intensity of the MICA-129Val variant. On CD8^+^ T cells, the MICA-129Met variant led to a slightly earlier co-stimulatory activation than the MICA-129Val variant. Importantly, the MICA-129Met variant induced a stronger down-regulation of NKG2D on NK cells and even more on CD8^+^ T cells than the MICA-129Val isoform. Down-regulation of NKG2D on CD8^+^ T cells impaired their capability to receive co-stimulatory signals via NKG2D. Hence, the stronger capacity of the MICA-129Met variant to signal via NKG2D itself limited the biological effects of an increased MICA-129Met expression intensity by a reduction in NKG2D expression. In other words, MICA-129Met variants, which elicit strong NKG2D responses, stimulate in parallel a robust negative feedback signal.

The counter-regulation of NKG2D could be a fairly uncomplicated mechanism contributing to the tuning of NKG2D responses with respect to MICA expression intensities, which has been reported recently (Shafi *et al*, 2011). In this study investigating several MICA-129Met but no MICA-129Val isoforms, the highest functional responses of NK and γδT cells were also not elicited by targets with highest MICA expression intensities. It has been suggested that NKG2D-mediated responses are tuned to an optimum against the MICA expression intensity that is most commonly induced by cellular dysregulation in an individual (Shafi *et al*, 2011).

**Figure 8:**
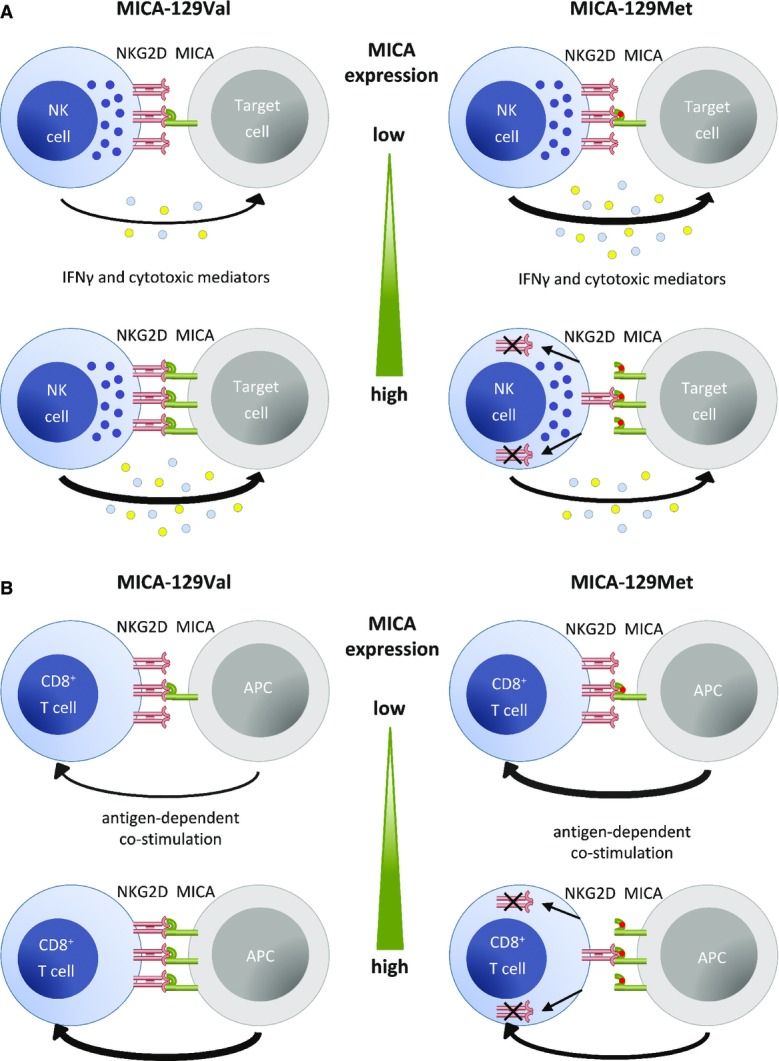
Summary of functional effects of MICA-129 variants depending on expression intensity For target cells expressing the MICA-129Val variant, the degree of NK-cell cytotoxicity and IFNγ production increased steadily with the MICA expression intensity. Augmented expression of the high-avidity MICA-129Met isoform, in contrast, had no or even a negative effect on these NK-cell functions due to a rapid down-regulation of NKG2D on NK cells.

In summary, the degree of NK-cell cytotoxicity and cytokine production increased steadily with the MICA expression intensity, if the MICA-129Val variant was present on target cells. Augmented expression of the MICA-129Met isoform, in contrast, had no or even a negative effect on NK-cell function (Fig8A). On CD8^+^ T cells, co-stimulation with the MICA-129Met variant allowed for an earlier antigen-dependent activation than the MICA-129Val variant, but a rapid down-regulation of NKG2D impaired any subsequent NKG2D-dependent co-stimulation (Fig8B). This interaction between MICA-129 genotype and MICA expression intensity is likely important for understanding of MICA-129 disease associations. Notably, these data show that the MICA-129Met/Val dimorphism affects differently NKG2D signaling and NKG2D expression at the plasma membrane (see Table3 for a summary). On the basis of this model, we could build hypotheses on clinical outcomes expected to be associated with this SNP after HSCT (Table3).

Antigen-dependent co-stimulation of CD8^+^ T cells with the MICA-129Met variant allowed for an earlier antigen-dependent activation than co-stimulation with the MICA-129Val variant. However, the down-regulation of NKG2D in response to MICA-129Met ligands impaired any subsequent NKG2D-dependent co-stimulation and T-cell activation. The down-regulation of NKG2D on CD8^+^ T cells was augmented with MICA-129Met expression intensity.

**Table 3:** Summary of biological effects of *MICA-129* genotypes and their relation to expected and observed clinical outcomes after HSCT

*MICA-129* genotype	NKG2D signaling	NKG2D cell surface expression over time	HSCT outcomes	Clinical outcomes expected in view of biological function	Clinical outcomes observed
*Met/Met*	strong	reduced	aGVHD	↑^a^ or ↓	↑ occurrence^b^ ↓ fatal^c^
			cGVHD	↓	↓^d^
			relapse	↑	↑^d^
			overall survival	variable	↑^c^
*Met/Val*	intermediate	reduced	aGVHD	→ or ↓	↓ occurrence^b^ ↓ fatal^c^
			cGVHD	↓	
			relapse	↑	
			overall survival	variable	↑^c^
*Val/Val*	weak	preserved	aGVHD	↓ or ↑	↓ occurrence^b^ ↑ fatal^c^
			cGVHD	↑	↑^d^
			relapse	↓	↓^d^
			overall survival	variable	↓^c^

a ↑ increased, → unchanged, ↓ decreased.

b Attributable to the group receiving a T-cell-depleting treatment with ATG in our study.

c Attributable to the group not treated with ATG in our study.

d Data from Boukouaci *et al* (2009).

After HSCT, NKG2D-mediated co-stimulation of CD8^+^ T cells presumably contributes to both GVHD and graft-versus-leukemia (GVL) reactions and similarly, NKG2D-mediated NK-cell activation potentially contributes to GVL effects and to protection against pathogens such as cytomegalovirus (Leung, 2011). MICA-129Met variants eliciting immediately a stronger CD8^+^ T- and NK-cell activation might therefore contribute to the occurrence of aGVHD. MICA is constitutively expressed in gastrointestinal epithelium (Groh *et al*, 1996), a target organ of GVHD, and can be up-regulated in other tissues under conditions of inflammation (Raulet *et al*, 2013). Inflammation is known to occur frequently after preconditioning, and MICA/B expression was indeed found in skin and liver during aGVHD (Gannage *et al*, 2008). Non-professional antigen-presenting cells that express MICA upon cellular stress become able to activate CD8^+^ T cells directly. This pathway presumably contributes to the initiation of aGVHD, if MICA is expressed on normal cells, such as endothelial or epithelial cells, and alloreactive CD8^+^ T cells become activated. MICA on leukemic cells could help to initiate GVL reactions by co-stimulation of anti-leukemic CD8^+^ T cells. However, the severity of aGVHD and the risk of cGVHD but also the probability of strong GVL effects could be limited by down-regulation of NKG2D on CD8^+^ T cells. In a murine model of HSCT, it has been recently demonstrated that NKG2D indeed contributes to aGVHD and graft-versus-tumor effects (Karimi *et al*, 2015).

MICA can be expressed on leukemic (Salih *et al*, 2003; Sconocchia *et al*, 2005; Boissel *et al*, 2006; Kato *et al*, 2007; Diermayr *et al*, 2008; Nückel *et al*, 2010; Sanchez-Correa *et al*, 2011; Hilpert *et al*, 2012) and on lymphoma cells (Dulphy *et al*, 2009; Reiners *et al*, 2013). MICA-129Met variants eliciting earlier or stronger CD8^+^ T- and NK-cell activation could in principal also allow for faster eradication of those malignant cells. However, the loss of NKG2D expression in response to engagement of a MICA-129Met variant would likely be more important over time. Therefore, a decreased NK and CD8^+^ T-cell activity against malignant cells expressing NKG2D ligands would be expected to increase the risk of a relapse in carriers of a *MICA-129Met* allele. Notably, not only NK cells but also *in vitro*-activated CD8^+^ T cells were reported to kill malignant cells expressing NKG2D ligands in an T-cell receptor-independent but NKG2D-dependent manner (Dhanji *et al*, 2004; Verneris *et al*, 2004; Karimi *et al*, 2005) and such cells have even been used in adoptive immunotherapy after HSCT (Laport *et al*, 2011; Meehan *et al*, 2013).

In our cohort, the homozygous carriers of MICA-129Met alleles had indeed an increased risk to experience aGVHD, which could be the consequence of immediate effects of MICA-129Met variants on NKG2D signaling (Table3). The *MICA-129Met* alleles appeared to support the initiation of aGVHD in homozygous carriers especially in patients receiving ATG. In this subgroup with an overall substantially reduced risk to develop aGVHD, the faster co-stimulatory activation of residual CD8^+^ T cells by the MICA-129Met variant could be important, whereas this difference might not matter if a full CD8^+^ T cell repertoire is present in patients. Alternatively, pro-inflammatory cytokines, such as IFNγ released by NK cells in response to stimulation of NKG2D, could contribute to aGVHD (Ferrara *et al*, 1989). On the other hand, having at least one *MICA-129Met* allele conferred a lower probability to develop a severe or fatal aGVHD. When the patients were stratified according to treatment with ATG, this beneficial *MICA-129Met* association was attributed to the subgroup not receiving ATG. This finding appears to be explainable by a rapid NKG2D down-regulation on alloreactive CD8^+^ T cells mediated by engagement of at least one high-avidity MICA-129Met variant limiting the NKG2D-mediated co-stimulation of alloreactive donor CD8^+^ T cells. In heterozygous patients, also the risk of occurrence of aGVHD was reduced, suggesting that the effect of MICA-129Met variants on the expression of NKG2D is decisive for this outcome. In consequence, we found overall an increased probability of survival after HSCT for patients carrying a *MICA-129Met* allele in our cohort. Consistently, patients who carried two *MICA-129Val* alleles were at risk to develop a severe or fatal aGVHD and they appeared to particularly profit from ATG treatment. This could be explained by a failure to efficiently down-regulate NKG2D on alloreactive CD8^+^ T cells, and this finding is potentially of therapeutic relevance for patients carrying two *MICA-129Val* alleles. However, ATG can affect in addition to T cells also other immune cell types including NK cells and B cells (Hoegh-Petersen *et al*, 2013), which also might have contributed to the effects observed in our cohort.

It has been previously reported that the risk of cGVHD was increased for recipients with the *MICA-129Val/Val* genotype, whereas the MICA-129Met/Met genotype was associated with the risk of relapse (Boukouaci *et al*, 2009). In our cohort, the *MICA-129Met/Met* genotype was similarly associated with a trend toward a higher mortality due to relapse in patients not treated with ATG (HR = 3.43, *P *=* *0.1450). Notably, the associations reported (Boukouaci *et al*, 2009) are also well explainable by effects on NKG2D (Table3). Sustained NKG2D-mediated activation of alloreactive CD8^+^ T cells would be expected if only a MICA-129Val variant is present, and this could increase the risk of cGVHD. Sustained NKG2D-mediated activation of anti-leukemic CD8^+^ T cells and NK cells would be expected to reduce the risk of relapse. Thus, the different risk associations reported herein and the previous study by Boukouaci *et al* are not arguing against the relevance of the MICA-129 dimorphism for the outcome of HSCT. They rather point toward differences in the relative importance of specific outcomes such as aGVHD, cGVHD, and relapse in the two cohorts due to clinical reasons. The incidence of cGVHD, for example, was 47% (Boukouaci *et al*, 2009) versus 30.5%, and effects on a specific outcome are of course more likely detectable if the respective outcome is more frequent. Similarly, the effect of the SNP on overall survival could vary in different cohorts depending on the relative importance of aGVHD, cGVHD, and relapse for survival in a cohort. The principal relevance of NKG2D signaling for the outcome of allogeneic HSCT is further emphasized by studies demonstrating an effect of the genotype of the NKG2D ligand *RAET1L* (Antoun *et al*, 2012) and *NKG2D* (Espinoza *et al*, 2009) itself on overall survival of patients.

The MICA-129 dimorphism could also be relevant for the success of immunotherapies using NK cells to treat MICA-positive malignancies (Laport *et al*, 2011; Leung, 2011; Meehan *et al*, 2013). Moreover, it might be highly important when NKG2D signaling is targeted by new therapies aiming at up-regulation of MICA by histone deacetylase inhibitors (Kato *et al*, 2007; Diermayr *et al*, 2008) or blocking of signaling by soluble NKG2D (Hilpert *et al*, 2012).

Our experimental data showed that the MICA-129Met variant triggers more NKG2D signals at low expression intensities, whereas MICA-129Val variant elicits more NKG2D effects at high expression, at which the MICA-129Met variant already down-regulates NKG2D leading to impaired function. Thus, expression intensity can change the biological effect of this SNP. Both variants may confer advantages and disadvantages in certain situations, such as virus infections, suggesting balancing evolution of *MICA* alleles. Differences in MICA expression intensities in GVHD-affected tissues (Gannage *et al*, 2008) and on malignant cells (Sconocchia *et al*, 2005; Boissel *et al*, 2006; Nückel *et al*, 2010; Hilpert *et al*, 2012) could therefore affect the association of the SNP with specific outcome parameters after HSCT. Notably, MICA expression intensities can vary for certain *MICA* alleles (Shafi *et al*, 2011). A further modifying factor is sMICA (Boukouaci *et al*, 2009), which was found in variable amounts in patient sera (Salih *et al*, 2002; Boissel *et al*, 2006; Nückel *et al*, 2010; Hilpert *et al*, 2012). Moreover, anti-MICA Ab (Boukouaci *et al*, 2009), neutralizing sMICA but also sensitizing MICA-expressing cells to complement-dependent cellular cytotoxicity (Zou *et al*, 2002; Jinushi *et al*, 2006; Zou & Stastny, 2010), might additionally modulate the effects of the SNP MICA-129.

In conclusion, we have shown that the MICA-129Val/Met dimorphism affects the strength and kinetics of NKG2D signaling resulting in differences of NK-cell cytotoxicity and cytokine secretion as well as CD8^+^ T-cell co-stimulation. It also affects NKG2D expression on NK and even more on CD8^+^ T cells. These functional effects of the dimorphism were critically modulated by the MICA expression intensity. The MICA-129 dimorphism had a significant impact on the outcome of HCST in this and in a previous independent study (Boukouaci *et al*, 2009). Our data suggest that patients carrying a MICA-129Met allele have a reduced risk of fatal aGVHD, whereas carriers of a MICA-129Val/Val genotype are at high risk and appear to profit from ATG treatment. Potentially, NKG2D itself could be a target for prevention or therapy of GVHD.

## Materials and Methods

### Patie nts and treatments

Approval for the analysis has been obtained from the Institutional Review Board of UMG, and it has been conducted according to the Declaration of Helsinki. Informed consents to transplantation and retrospective outcome analysis have been obtained from all patients. The patient materials and clinical data are owned by UMG. Their use requires Institutional Review Board approval. Transplantation indications and conditioning protocols followed European Group for Blood and Marrow Transplantation (EBMT) guidelines or clinical trial protocols, whenever pertinent. The MICA-129 genotyping results were obtained retrospectively and not taken into consideration for any treatment decisions. Prophylaxis of GVHD started on day –1 and consisted of cyclosporine (target trough level 100–300 μg/l) or tacrolimus (target trough level 8–12 μg/l) and mycophenolate mofetil (1 g twice a day until day 28 or day 50, depending on the protocol). ATG was applied to patients mismatched and most unrelated transplantations. Either 10 mg/kg bw (body weight) ATG-Fresenius S (Fresenius Biotech, Grafelfing, Germany) or 2.0 mg/kg bw thymoglobuline (Genzyme Europe, Naarden, the Netherlands) was given from day −3 to day 1. Acute and chronic GVHD were scored according to international standards (Przepiorka *et al*, 1995). Cases of aGVHD were categorized into groups of overall grades 0 and I–IV, and cases of cGVHD were categorized into absent, mild, moderate, and severe, respectively (Filipovich *et al*, 2005).

### Statistical analyses

Statistical analyses of clinical and genotyping data were performed with R software (http://www.R-project.org). The influence of the MICA-129 dimorphism on the following outcome parameters were evaluated: overall mortality (time to event analysis with Cox proportional hazard models), TRM, mortality due to aGVHD, mortality due to relapse, occurrence of aGVHD (0 versus grades I–IV), severity of aGVHD (grades I and II versus grades III and IV), occurrence of cGVHD (absent versus mild, moderate, or severe), and occurrence of relapse (probability of event analyses with logistic regression). The analyses were performed after adjustment for the relevant clinical covariates: T-cell depletion, total body irradiation, HLA-matched unrelated donor, female donor for male recipient, and diagnosis group (Table2). In addition, all analyses were adjusted for a binary indicator distinguishing whether patient and donor had the same MICA-129 genotype or not. Other parameters, including having a less than 8/8 HLA-matched unrelated donor, reduced intensity conditioning, and disease status, were considered but not identified as significant covariates. The co-dominant model was employed to determine the most appropriate inheritance risk model (additive, dominant, or recessive). Reported are the results of the most appropriate genetic model. Power for detected effects ranged between 52% (recessive model) and 86% (additive model) at the 5% significance level.

Sample sizes for *in vitro* experiments were estimated based on previous experiences with similar assays. Typical group sizes for MICA-129 strata such as L-MICA-129Met and L-MICA-129Val clones were ≥ 20 and balanced, yielding a power ≥ 80% for the detected main effects of MICA-129. No samples were excluded from analysis. The experimental data were evaluated with the WinStat software (R. Fitch Software, Bad Krozingen, Germany) employing the Kolmogorov–Smirnov test to determine normal distribution and Pearson correlation, *t*-tests, or two-way ANCOVAs with interactions for subsequent analyses. The nonparametric Wilcoxon test was used to compare non-normally distributed target variables. A *P*-value of < 0.05 in two-sided tests was considered significant. The SAS software (Cary, North Carolina, USA) was used to estimate ANOVA or ANCOVA-like linear mixed models with adjustment of parameters for the interaction with MICA expression intensity. Dependent on the experimental design, random effects were included into the models to account for longitudinal correlation (cell surface expression of NKG2D and CD94; relative cell lysis) or a random trial effect (IFNγ). While *t*-tests allowed for different group variances where appropriate, ANCOVA-like models used a pooled estimate of standard deviation (SD) for robust inference.

### Materials and antibodies

Unless specified otherwise, all chemicals were from Sigma-Aldrich (Taufkirchen, Germany) or Merck (Darmstadt, Germany), all enzymes from New England Biolabs (Ipswich, MA, USA), and all cell culture plastic materials from Nunc (Roskilde, Denmark) or Sarstedt (Nümbrecht, Germany). Antibodies (Ab) used are listed in the Appendix Table S2.

### MICA-129 genotyping

The SNP rs1051792 (G/A) leading to a substitution of Val (G) by Met (A) at position 129 of MICA was genotyped by a TaqMan assay (Applied Biosystems, Foster City, CA, USA) containing the forward primer 5′-GCTCTTCCTCTCCCAAAACCT-3′ and the reverse primer 5′-CGTTCATGGCCAAGGTCTGA-3′ and the two allele-specific dye-labeled probes FAM-5′-AATGGACA**G**TGCCCC-3′ and VIC-5′-AATGGACA**A**TGCCCC-3′.

### MICA-129 expression constructs

A pCMV6-AC expression vector containing the *MICA* gene (Origene, Rockville, ML, USA) was altered by site-directed mutagenesis at two positions (codons for amino acids 24 and 360) to obtain the MICA*00701 allele. This allele has a methionine at amino acid position 129. The MICA-129Val variant (pCMV6-AC-MICA-129Val) was generated by mutagenesis of the pCMV6-AC-MICA-129Met construct. To obtain MICA proteins as mouse IgG_2a_-Fc fusion proteins (MICA-129Met-mIgG_2a_-Fc and MICA-129Val-mIgG_2a_-Fc), the extracellular parts of MICA were amplified by polymerase chain reaction (PCR) from pCMV6-AC-MICA-129Met and pCMV6-AC-MICA-129Val vectors. As control, an ovalbumin (OVA) fusion protein construct (OVA-mIgG_2a_-Fc) was generated. Primers with restriction sites for *Kpn*I (MICA-129-Fc_fwd: 5′-TGGTACCATGGGGCTGGGCCCGGTCTTCCTGC-3′; OVA-Fc_fwd: 5′-CAGGTACCATGGGCTCCATCGGCGCAGCAA-3′) or *BamH*I (MICA-129-Fc_rev: 5′-CGGATCCTGAAGCACCAGCACTTTCCCAGA-3′; OVA-Fc_rev: 5′-TGAGGATCCATAGGGGAAACACATCTGCCAAAG-3′) were used, and the PCR products were inserted into the pcDNA3.1/*myc*-His A(+) expression vector (Invitrogen, Darmstadt, Germany) that already contained the mouse IgG_2a_-Fc cDNA, including the hinge and the CH2 and CH3 regions, derived from the pFUSE-mIgG_2a_-Fc1 vector (InvivoGen, Toulouse, France). All constructs were sequenced before use.

### Cell culture and transfections

Mouse fibroblast L cells, human embryonic kidney (HEK) 293 cells, and K562 cells were maintained in NaHCO_3_-buffered Dulbecco’s modified Eagle’s medium (DMEM) supplemented with 10% fetal calf serum (FCS; Biochrom, Berlin, Germany), 2 mM L-glutamine, 1 mM sodium pyruvate, 50 μM 2-mercaptoethanol, 100 U/ml penicillin, and 100 μg/ml streptomycin. All cell lines were tested routinely by PCR to exclude mycoplasma contamination. L cells were transfected by electroporation with 50 μg of *Pvu*I-linearized pCMV6-AC-MICA-129Met or pCMV6-AC-MICA-129Val constructs or the empty pCMV6-AC vector. After selection (1 mg/ml G418, Biochrom, Berlin, Germany), clones (L-con, L-MICA-129Met, and L-MICA-129Val) were obtained by limiting dilution. HEK293 cells were transfected with 50 μg *Pvu*I-linearized DNA of MICA-129Met-Fc, MICA-129Val-Fc, or OVA-Fc constructs. After selection (0.5 mg/ml G418), fusion protein secretion into the supernatant was analyzed by an ELISA for mouse IgG.

### NK cells and CD8^+^ T cells

Peripheral blood mononuclear cells (PBMC) were obtained from blood of healthy donors by centrifugation on Biocoll separating solution (Biochrom). PBMC were cultured for 4 days with 100 U/ml human IL-2 (Proleukin, Chiron, Amsterdam, the Netherlands) to obtain LAK cells. NK cells were isolated from PBMC by magnetic-activated cell sorting (MACS) using a negative selection kit (NK cell isolation kit II, Miltenyi Biotec, Bergisch-Gladbach, Germany) and either used directly or cultured for 4 days with 100 U/ml IL-2. Purity of NK cells was evaluated by flow cytometry (Appendix Fig S15A). CD8^+^ T cells were isolated from PBMC by MACS using a negative selection kit (CD8^+^ T cell isolation kit, Miltenyi Biotec). Purity of CD8^+^ T cells was evaluated by flow cytometry (Appendix Fig S15B).

### Enzyme-linked immuno-sorbent assay (ELISA)

To measure the secretion of MICA-129Met/Val-mIgG_2a_-Fc and OVA-mIgG_2a_-Fc proteins from transfected clones of HEK293 cells, 96-well Nunc MaxiSorp microtiter plates were coated overnight at 4°C with 10 μg/ml goat anti-mouse IgG Ab in sodium carbonate buffer (pH 8.5; 50 μl/well). After blocking with 1% gelatin in phosphate-buffered saline (PBS), cell culture supernatants (50 μl/well) were added and the plates were incubated for 1 h at 37°C. For detection, a goat anti-mouse horseradish peroxidase (HRP)-conjugated Ab diluted 1:4,000 in PBS with 0.05% Tween-20 was used. After incubation (1 h at 37°C), 50 μl 2,2′-azino-bis(3-ethylbenzothiazoline-6-sulfonic acid; ABTS) substrate solution was added to each well and the optical density was immediately determined using a BioTek PowerWave 340 microplate spectrophotometer (BioTek, Bad Friedrichshall, Germany) set to 405 nm. IFNγ, TNF-α, IL-10, and IL-13 concentrations in the supernatant of NK cells co-cultured with L-con, L-MICA-129Met, or L-MICA-129Val clones (5 × 10^4^ targets plus 2.5 × 10^5^ NK cells in 200 μl medium) for 4–24 h or cultured on plate-bound MICA-129Met-Fc, MICA-129Val-Fc, or OVA-Fc proteins (2.5 × 10^5^ NK cells in 200 μl medium) were determined by human IFNγ, TNF-α, IL-10, and IL-13 ELISA sets (ImmunoTools, Friesoythe, Germany). IL-2 release from CD8^+^ T cells was measured by human IL-2 ELISA set (MAX Standard, Biolegend, Fell, Germany). Concentrations of sMICA in the supernatants of L-MICA-129Met/Val cells (1 × 10^6^ cells, 10 ml medium, 24 h) were determined using the human MICA DuoSet (R&D Systems, Wiesbaden, Germany). These assays were performed according to the manufacturer′s protocols. All samples were analyzed in duplicate or triplicates in comparison to a standard curve of IFNγ, TNF-α, IL-2, IL-10, IL-13, MICA, or mouse IgG protein, respectively.

### Production and purification of recombinant MICA-129Met-Fc and MICA-129Val-Fc fusion proteins

To obtain supernatant containing the fusion proteins, 5 × 10^7^ HEK293-MICA-129Met-Fc, HEK293-MICA-129Val-Fc, or HEK293-OVA-Fc cells were cultured in 75 ml FCS-free DMEM for 3 days. The cell culture supernatants were dialyzed in 20 mM sodium phosphate buffer (pH 7.0) overnight at 4°C in a dialysis tubing with a molecular weight cutoff (MWCO) of 12–14 kDa (SERVA Electrophoresis GmbH, Heidelberg, Germany). Subsequently, the fusion proteins were purified using 1-ml HiTrap-Protein-G-HP columns (GE Healthcare, Freiburg, Germany) according to the manufacturer’s instructions. After buffer exchange in PBS (pH 7.2), the proteins were concentrated using Amicon filter units with 30-kDa MWCO (Merck Millipore, Darmstadt, Germany). Bradford assays (Bio-Rad Laboratories GmbH, Munich, Germany) and reducing sodium dodecyl sulfate-polyacrylamide gel electrophoresis (SDS–PAGE) were performed to determine the concentration and purity of the MICA-129Met-Fc, MICA-129Val-Fc, and OVA-Fc proteins. Glycosylation was tested by digestion with Endo H (New England Biolabs, Ipswich, MA, USA).

### Surface plasmon resonance (SPR) analysis

SPR measurements were done using a Reichert SPR Biosensor SR7500DC with a HC 1000 m sensorchip (Xantec Bioanalytics, Düsseldorf, Germany). All measurements were performed in running buffer containing PBS, pH 7.4, at a flow rate of 40 μl/min. NKG2D-Fc was covalently immobilized on the EDC/NHS-activated left channel (sample channel) of the sensorchip, at a concentration of 200 nM, at a flow rate of 30 μl/min, to a response level of 2,500 response units (RU). The right channel of the chip served as a reference. Increasing concentrations of analyte (MICA-129Met-Fc or MICA-129Val-Fc) were injected for 270 s over both channels, and dissociation was followed for 15 min. Kinetic analysis was performed using Scrubber 2.0 (BioLogic Software, Campbell, Australia). The recorded responses were double referenced (right channel, buffer blank) and normalized using the molecular weight of the analyte (in kDa).

### SDS–PAGE and immunoblotting

NK cells were stimulated with the fusion proteins as described below (CD107a degranulation assay), before Nonidet P-40 (NP-40) lysates were prepared for Western blot analyses. A total of 1 × 10^6^ NK cells were harvested at 4°C for each time point either unstimulated or stimulated at 37°C with MICA-129Met-Fc, MICA-129Val-Fc, or OVA-Fc proteins for 3, 10, or 30 min, respectively. The cells were lysed in 25 μl NP-40 buffer (1%) before 25 μl reducing loading buffer was added. After incubation for 4 min at 95°C, the lysates were loaded on 4–12% SDS gels for electrophoresis at 20 to 40 mA for approximately 3 h. Then, the proteins were blotted onto a nitrocellulose membrane (Roth, Karlsruhe, Germany) for 1 h using a semi-dry blotting technique (1 mA/cm^2^). The membrane was blocked in Tris-buffered saline with 0.1% Tween-20 (TBS-T) with 5% bovine serum albumin (BSA) for 1 h, washed, and then incubated with specific primary Ab in TBS-T together with 1% BSA overnight at 4°C. After being washed three times for 10 min in TBS-T, the membrane was incubated with a HRP-labeled secondary Ab or, for the detection of MICA, with HRP-conjugated streptavidin (BioLegend). Detection was done using an enhanced chemiluminescence (ECL) kit (GE Healthcare), and chemiluminescence was measured using a digital image acquisition system (Intas Chemilux Entry, Intas, Göttingen, Germany). The MICA-129Met-Fc, MICA-129Val-Fc, and OVA-Fc fusion proteins were separated by SDS–PAGE together with BSA or OVA as control proteins. The gels were stained with Coomassie Brilliant Blue R250 for 15 min followed by a 30-min wash with 10% acetic acid and 30% methanol and documented using the Intas gel manager (Intas, Göttingen, Germany).

### Flow cytometry

Flow cytometry was performed with a FACSCalibur flow cytometer and CellQuestPro software (BD Biosciences, Heidelberg, Germany). Cell surface expression of MICA on propidium iodide (PI)-negative cells was examined using the anti-MICA monoclonal antibody (mAb) AMO1 (1 μg/10^6^ cells in 100 μl PBS; for Ab see Appendix Table S2) and fluorescein isothiocyanate (FITC)-conjugated goat anti-mouse IgG Ab as secondary reagent. This secondary Ab was also used to detect the binding of the recombinant MICA-129Met-Fc, MICA-129Val-Fc, or OVA-Fc fusion proteins to NK cells. Binding of a recombinant human NKG2D-Fc fusion protein (139-NK, R&D Systems) to MICA-expressing cells (0.2 μg/10^6^ cells in 100 μl PBS) was assessed using a polyclonal FITC-conjugated goat anti-human IgG Ab as secondary reagent. PBMC and NK cells were characterized using mAb against CD3, CD14, CD16, CD19, CD56, CD94, NKp30 (CD337), NKp44 (CD336), NKp46 (CD335), NKG2D (CD314), and γ/δ T-cell receptor (TCR). CD8^+^ T cells were characterized with mAb against CD3, CD8, CD28, and NKG2D. All stainings were done at 4°C in the dark. Isotype controls were purchased from ImmunoTools or BioLegend. NKG2D expression and CD94 expression were also measured after co-culture for 4 or 24 h of 2.5 × 10^5^ purified IL-2-stimulated NK cells with 5 × 10^4^ target cells per well of a 96-well plate. NKG2D expression and CD8 expression were measured after co-culture for 4 or 24 h of 2.5 × 10^5^ purified CD8^+^ T cells with 5 × 10^4^ target cells per well of a 96-well plate. A potential cytotoxic effect of the SRC kinase inhibitor PP2 (25 μM; Sigma-Aldrich, #P0042) was determined by staining of NK cells with Annexin V-FITC (BD Biosciences) in combination with PI in binding buffer according to the manufacturer’s protocol.

### CD107a degranulation assay

To determine the degranulation of NK cells exposed to L-con, L-MICA-129Met, or L-MICA-129Val target cells, 10^6^ LAK cells and 4 × 10^4^ targets were co-cultured for 1 h at 37°C in 100 μl medium (E:T ratio 25:1), and an anti-CD107a mAb (4 μl) or the respective isotype control (mouse IgG_1_) was added. Alternatively, 2 × 10^5^ purified NK cells were co-cultured in 100 μl medium with 5 × 10^4^ target cells (E:T ratio 4:1). Afterward, the cells were harvested and stained at 4°C with anti-CD16 and anti-CD56 mAb to identify CD107a^+^ NK cells. To elicit degranulation of purified IL-2-stimulated NK cells with immobilized MICA-129Met/Val-mIgG_2a_-Fc or OVA-mIgG_2a_-Fc fusion proteins, MaxiSorp microtiter plates (Nunc) were pre-coated with 1 μg/well of a goat anti-mouse F(ab’)_2_ fragment in 100 μl sodium carbonate coating buffer (pH 8.5) overnight at 4°C. After washing with PBS, the MICA-129Met-Fc, MICA-129Val-Fc, or OVA-Fc proteins were added at different concentrations in 100 μl PBS/well and incubated for 1 h at room temperature. Then, the plates were washed before IL-2-stimulated NK cells (2.5 × 10^5^/well in 100 μl medium) and the CD107a mAb or the isotype control were added. The plates were incubated at 37°C for 1 h before the cells were harvested and stained to identify CD107a^+^ NK cells. In some assays, the SRC kinase inhibitor PP2 (25 μM) or dimethyl sulfoxide (DMSO) as solvent was given to the NK cells 30 min before being added to the plates.

### Intracellular staining of IFNγ

To determine the IFNγ expression in NK cells exposed to L-con, L-MICA-129Met, or L-MICA-129Val target cells, 2 × 10^5^ NK cells were co-cultured in 100 μl medium for 4 h at 37°C with 5 × 10^4^ targets (E:T ratio 4:1). Afterward, the cells were harvested and counterstained at 4°C with anti-CD16 and anti-CD56 mAb before fixation and permeabilization with Cytofix/Cytoperm and Perm/Wash solutions (BD Biosciences) according to the manufacturer’s protocol. The cells were then stained with an anti-IFNγ mAb or an isotype control suitable for intracellular staining experiments (mIgG_1_, MOPC-21; Biolegend) for 30 min at 4°C. To elicit IFNγ expression of purified IL-2-stimulated NK cells with immobilized MICA-129Met/Val-mIgG_2a_-Fc or OVA-mIgG_2a_-Fc fusion proteins, microtiter plates were prepared as described above. IL-2-stimulated NK cells (2.5 × 10^5^/well in 100 μl medium) were added, and the plates were incubated at 37°C for 4 h before the cells were harvested, stained at 4°C with anti-CD56 and anti-CD16 mAbs, and analyzed by flow cytometry.

### ^51^Cr release assay

Target cells were labeled by incubating 1 × 10^6^ cells in 200 μl DMEM containing 100 μl FCS and 50 μCi Na_2_^51^CrO_4_ (CrRA8, Hartmann Analytic, Braunschweig, Germany) for 1 h at 37°C and washed three times with DMEM. Effector cells were added to 5 × 10^3^ ^51^Cr-labeled target cells in triplicate at various ratios in 200 μl DMEM with 10% FCS per well of round-bottomed microtiter plates. Spontaneous release was determined by incubation of target cells in the absence of effector cells. The microtiter plates were centrifuged for 5 min at 40 × *g*, incubated at 37°C for 4 h, and then centrifuged again. Supernatant and sediment were separately taken to determine radioactivity in each well using a MicroBeta^2^ counter (PerkinElmer Life Sciences, Köln, Germany). Percentage of specific lysis was calculated by subtracting percent spontaneous ^51^Cr release.

### Proliferation assays

Proliferation of CD8^+^ T cells was analyzed after stimulation by a plate-bound anti-CD3 mAb delivering a first signal in combination with anti-CD28, anti-NKG2D, or MICA-129Met-Fc or MICA-129Val-Fc proteins delivering second or co-stimulatory signal. OVA-Fc (having a mIgG_2a_ Fc fragment), mIgG_2a_ (A111-3, BD Biosciences), and mIgG_1_ (C76-47, BD Biosciences) served as controls. 96-well Nunc MaxiSorp microtiter plates were coated overnight at 4°C with 10 μg/ml goat anti-mouse IgG F(ab’)_2_ fragments in sodium carbonate buffer (pH 8.5; 100 μl/well). After washing with PBS, stimulatory mAb or recombinant Fc fusion proteins or isotype controls were added at the indicated concentrations and incubated at room temperature for 2 h. Then, the plates were washed again with PBS before 1 × 10^5^ freshly isolated CD8^+^ T cells per well were added in 200 μl medium with 10% FCS. After 72 h, 50 μl medium was harvested for IL-2 measurements and replaced by the same volume containing 1 μCi [methyl-^3^H]thymidine (MT 6031, Hartmann Analytic). Following a further incubation of 12 h, the DNA-bound radioactivity was harvested with a FilerMate Harvester (Perkin Elmer) and measured with MicroBeta^2^ Counter (Perkin Elmer). The SI was calculated by dividing the measured counts per minute (cpm) by the cpm of the negative control (unstimulated CD8^+^ T cells). In other experiments, purified CD8^+^ T cells were labeled by the dye CFSE (C-1157, Invitrogen, Karlsruhe, Germany) before culture on the coated plates. Cells were incubated for 5 min with 5 μM CFSE in PBS/0.1% BSA at 37°C. After being washed 3 times with DMEM containing 10% FCS, the cells were cultured as described above. The cells were harvested at the indicated time points, stained by anti-CD3 and anti-CD8 mAbs, and analyzed by flow cytometry.

## For more information

http://www.ebi.ac.uk/imgt/hla/

## Supporting Information

**Appendix**

**Expanded View Figures PDF**

**Review Process File**
