# Supplementary material for: The MICA-129 dimorphism affects NKG2D signaling and outcome of hematopoietic stem cell transplantation
Source: EMBO Mol Med. 2015 Oct 19;7(11):1480–502. doi: 10.15252/emmm.201505246 (PMC4644379; doi:10.15252/emmm.201505246)
Supplement: Supplementary file 1 [file emmm0007-1480-sd1.pdf]

EMBO Molecular Medicine

Antje Isernhagen, Dörthe Malzahn, Elena Viktorova, Leslie Elsner, Sebastian Monecke, Frederike von Bonin, Markus Kilisch, Janne Marieke Wermuth, Neele Walther, Yesilda Balavarca, Christiane Stahl-Hennig, Michael Engelke, Lutz Walter, Heike Bickeböller, Dieter Kube, Gerald Wulf, & Ralf Dressel

The MICA-129 dimorphism affects NKG2D signaling and outcome of hematopoietic stem cell transplantation

Appendix

Table of content

- Legends for Appendix Figures
- Appendix Figures (S1 to S15)
- Appendix Tables (S1 to S2)

Legends for Appendix Figures

Appendix Figure S1. L-MICA-129Met cells bind NKG2D with higher avidity than L-MICA-129Val cells. (**A**) The expression of MICA was analyzed by flow cytometry on L-con, L-MICA-129Met, and L-MICA-129Val cells using a FITC-conjugated goat anti-mouse IgG as secondary Ab. In parallel, the binding of a recombinant human NKG2D-Fc fusion protein was measured using FITC-conjugated goat anti-human IgG as secondary Ab. Staining with the respective primary reagent plus secondary Ab (blue or red line) and FITC-labeled secondary Ab only (black line) is shown. The MFI of MICA expression and NKG2D binding determined by these measurements are indicated. (**B**) The MICA expression intensity on L-MICA-129Met (n=79) and L-MICA-129Val clones (n=81) was determined as MFI by flow cytometry as illustrated in panel A and is displayed in box-and-whisker plots. The data were compared by a t-test. (**C**) In parallel, the binding of a recombinant NKG2D-Fc fusion protein to the L-MICA-129Met and L-MICA-129Val clones was measured by flow cytometry and the MFIs are shown in box-and-whisker plots. A difference between the cell lines was assessed by a t-test. (**D**) The ratios of the NKG2D binding and MICA expression for the L-MICA-129Met and L-MICA-129Val clones are summarized in box-and-whisker plots revealing a difference between the MICA-129 variants in NKG2D binding avidity (t-test).

Appendix Figure S2. MICA-129Met-Fc and L-MICA-129Val-Fc proteins, in contrast to an OVA-Fc protein, bind to NK cells. (**A**) MICA-129Met-Fc and L-MICA-129Val-Fc proteins were separated together with a bovine serum albumin (BSA) standard and a marker (M) by SDS-PAGE and stained with Coomassie blue (left panel) to demonstrate the integrity of the recombinant fusion proteins. Both fusions proteins (5 µg) were separated by SDS-PAGE and blotted. The blot was probed with a biotinylated anti-MICA Ab (right panel). (**B**) The OVA-Fc protein was separated with a marker (M) by SDS-PAGE and stained with Coomassie blue (left panel). The OVA-Fc fusion protein (1 µg) was separated together with OVA (1 µg) by SDS-PAGE and blotted. The blot was probed with an anti-OVA Ab. (**C**) The binding of MICA-129Met-Fc, MICA-129Val-Fc, and as negative control OVA-Fc fusion proteins to purified IL-2- stimulated (100 U/ml for 4 days) NK cells was determined by flow cytometry.

The histograms illustrate the staining with the fusion proteins (10 µg/ml) plus secondary Ab in bold and with the secondary Ab only in light. The percentage of cells binding to the proteins and the MFI are indicated. (**D**) The binding of MICA-129Met-Fc, MICA-129Val-Fc, and OVA-Fc fusion proteins to NK cells was determined by flow cytometry at various concentrations and is shown as means (n=3) and SD of the MFI (left panel) and the percentage of positive cells (right panel) . Notably, the binding decreased at very high concentrations of MICA proteins (100 µg/ml). Binding of MICA129Met-Fc and MICA-129Val-Fc proteins to NK cells was not significantly different. In contrast, we had observed a higher avidity of the MICA-129Met than MICA-129Val isoform for NKG2D using L cells expressing the MICA-129Met or MICA-129Val variants (see Appendix Fig S1D). This might be due to slight differences in the conformation or glycosylation of MICA molecules expressed in L cells or produced as IgG_{2a}-Fc fusion proteins in HEK293 cells. Notably, the MICA-129Met-Fc and MICA-129Val-Fc proteins varied in their efficacy to elicit downstream biological effects (see Fig 2B, Fig 2C, Fig 3A, Fig EV2, Fig EV3) similarly as MICA-129Met/Val molecules expressed on L cells.

Appendix Figure S3. MICA-129Met-Fc and MICA-129Val-Fc proteins bind with similar avidity to NKG2D in SPR analysis. (**A**) NKG2D-Fc was covalently immobilized on an activated HC 1000m SPR sensorchip and the SPR response was recorded after injecting 2, 4, 8, 16, 32, 64, 125, 250, and 500 nM of MICA-129Val-Fc or MICA-129Met-Fc fusion proteins. A typical sensogram of MICA129Val-Fc binding obtained via SPR is shown with observed binding (black lines) and global fit of SPR data using kinetic parameters k_a and k_d (orange lines). (**B**) Equilibrium binding analysis of NKG2D-Fc binding to MICA-129Met-Fc at 20 °C. (**C**) Equilibrium binding analysis of NKG2D-Fc binding to MICA-129Val-Fc at 20°C. (**D**) The equilibrium binding constants (K_D) of the MICA-129Met-Fc and MICA-129Val-Fc proteins to NKG2D-Fc were obtained using a nonlinear curve fit. The results of four experiments are summarized (means ± SD) and compared by a t-test.

Appendix Figure S4. **Resting NK cells fail to respond to L-MICA-129Met and L-MICA-129Val clones.** (**A**) A summary of 3 independent experiments is shown demonstrating the very low cytotoxic activity of freshly isolated NK cells against an L-MICA-129Met and an L-MICA-129Val clone. L-con cells served as negative and K562 cells as a positive control. The means of specific lysis plus SD at different E:T ratios (16:1 to 0.25:1) were measured in ^{51}Chromium-release assays. (**B**) The MICA expression intensity to the L target cells was determined in parallel by flow cytometry and the MFI as well as the percentage of MICA^{+} cells are displayed. (**C**) Freshly isolated NK cells were co-cultured with K562 target cells before measuring CD107a^{+} (2 h) and IFNγ^{+} cells (4 h) by flow cytometry on CD56^{dim}CD16^{+}, CD56^{bright}CD16^{+}, and CD56^{bright}CD16^{-} NK cells. In the shown experiment, 10.7% of the CD56^{dim}CD16^{+} NK cells were CD107a^{+} whereas very few NK cells responded to K562 cells by IFNγ production. (**D**) A summary (mean plus SD) of 3 independent flow cytometry experiments performed in parallel to the experiments in panels A and B is shown. (**E**) The release of cytokines (IFNγ, TNFα, IL-10, and IL-13) into the supernatant was determined in parallel to these experiments by ELISA after 4 and 24 h of co-culture (2.5x10^{5} NK cells plus 5x10^{4} targets). The means plus SD of 3 experiments are shown.

Appendix Figure S5. Inhibition of SRC family kinases by PP2 abolishes lysis of MICA expressing target cells and release of cytokines but does not induce apoptosis in NK cells. (**A**) One of 2 experiments with in principal identical results is shown demonstrating the inhibition of cytotoxic activity of purified IL-2-stimulated (100 U/ml for 4 days) NK cells against an L-MICA-129Met and an L-MICA-129Val clone by PP2 (25 µM). The means of specific lysis of triplicates plus SD at different E:T ratios (4:1 to 0.06:1) are shown as measured in a ^{51}Chromium-release assay. The MICA expression intensity and the percentage of MICA^{+} target cells was determined in parallel by flow cytometry and is indicated. As controls untreated cells (con) and cells treated with the solvent only (DMSO) are included. (**B**) The release of IFNγ and TNFα into the supernatant was determined by ELISA after 4 h of culture of 2.5x10^{5} purified IL-2-stimulated (100 U/ml for 4 days) NK cells (con) on plate-bound MICA-129Met-Fc, MICA-129Val-Fc, or OVA-Fc proteins (10 µg/ml). PP2 (25 µM) or the solvent only (DMSO) was added to the

cultures. Means plus SD of 3 experiments are displayed. (**C**) Purified IL-2-stimulated (100 U/ml for 4 days) NK cells (con) were cultured for 4 h in presence of PP2 (25 µM) or the solvent only (DMSO). The cells were stained by AnnexinV-FITC and PI and the percentage of early apoptotic cells (AnnexinV$^+$/PI$^-$) is indicated. (**D**) A summary (means plus SD) of 7 independent experiments is shown.

Appendix Figure S6. **NK cells degranulate in response to L-MICA-129Met and L-MICA-129Val clones.**

(**A**) PBMC were stimulated *in vitro* for 4 days with IL-2 (100 U/ml) and then characterized by flow cytometry for expression of the indicated cell surface markers. The percentage of cells positive for the various markers (mean plus SD, n=6) is displayed. (**B**) NK cells among the PBMC were identified by staining of CD16 and CD56. The CD56$^+$ NK cells were gated as illustrated in this dot plot for subsequent analysis of CD107a expression. In these experiments using unseparated IL-2-stimulated PBMC as effector cells, CD56^{bright} NK cells were too rare to analyze them separately. (**C**) The degranulation of CD56$^+$ NK cells exposed to L-con, L-MICA-129Met or L-MICA-129Val clones for 1 h was determined by flow cytometry after staining of CD107a. Black lines indicate CD107a staining while gray-lined histograms show the isotype control. The percentages of CD107a$^+$ cells and the MFI of CD107a of these measurements are indicated in the histograms.

Appendix Figure S7. The expression of IFNγ in CD56^{bright}CD16$^+$ and CD56^{dim}CD16$^+$ NK cells in response to the MICA-129Met and MICA-129Val isoforms.

The IFNγ expression of CD56^{bright}CD16$^+$ (**A**) and CD56^{dim}CD16$^+$ (**B**) NK cells exposed to L-MICA-129Met (n=23) or L-MICA-129Val clones (n=23) for 4 h was determined by flow cytometry as MFI of IFNγ (upper panels) and as percentage of IFNγ$^+$ cells (lower panels). The purified NK cells had been stimulated *in vitro* for 4 days with IL-2 (100 U/ml). In parallel, the MICA expression on target cells was determined. Displayed is the linear regression of IFNγ and MICA expression on targets, both determined as MFI, for the L-MICA-129Met (upper left panel) and L-MICA-129Val clones (upper right panel). The linear regression of the percentage of IFNγ$^+$ NK cells and MICA expression on targets (determined as MFI) is

shown for the L-MICA-129Met (lower left panel) and L-MICA-129Val clones (lower right panel). The coefficients of determination (R^2), the regression coefficients (reg. coeff.) and the *P*-values for Pearson correlation are indicated.

Appendix Figure S8. Cytokine release of NK cells in response to the MICA-129Met and MICA-129Val isoforms.

The release of IFNγ, TNFα, IL-10, and IL-13 into the supernatant was determined by ELISA after 4 h of culture of 2.5×10^5 purified IL-2-stimulated (100 U/ml, 4 days) NK cells on plate-bound MICA-129Met-Fc, MICA-129Val-Fc, and OVA-Fc proteins (0, 5, 10, 15 µg/ml). Means plus SEM of 6 experiments are displayed. The data were analyzed by ANCOVA adjusted for the protein concentration (5, 10, 15 µg/ml) and the *P*-values of significant differences between the MICA-129Met-Fc and MICA-129Val-Fc proteins are indicated in the panels for IFNγ and TNFα. In the panel for IL-13, the *P*-values indicate significant differences between the OVA-Fc and the MICA-129Met-Fc (red) or MICA-129Val-Fc (blue) proteins, respectively.

Appendix Figure S9. Down-regulation of NKG2D but not of CD94 on NK cells co-cultured with L-MICA-129Val and L-MICA-129Met clones. (**A**) NKG2D expression on purified IL-2-stimulated NK cells (100 U/ml for 4 days) co-cultured with L-con, L-MICA-129Met or L-MICA-129Val target cells for 24 h was analyzed by flow cytometry. NKG2D expression was analyzed after gating on $CD3^-CD56^+$ NK cells. Black-lined histograms represent the NKG2D staining while gray-lined histograms show the isotype control. The percentages of $NKG2D^+$ cells and the MFI of NKG2D are indicated in the histograms. (**B**) In parallel, the CD94 expression on the NK cells was determined. Black-lined histograms represent CD94 staining while gray lines show the isotype control. The percentages of $CD94^+$ cells and the MFI of CD94 of these measurements are indicated in the histograms. (**C**) CD94 expression on purified IL-2-stimulated NK cells (100 U/ml for 4 days) exposed to L-MICA-129Met (n=25) or L-MICA-129Val clones (n=25) for 0, 4,

and 24 h was analyzed by flow cytometry. The means and SD of the percentage of $CD94^+$ NK cells (left panel) and of the MFI of CD94 (right panel) are shown.

Appendix Figure S10. Correlation of NKG2D expression on NK cells with L-MICA-129Val and L-MICA-129Met expression on target cells. NKG2D expression on purified IL-2-stimulated NK cells (100 U/ml for 4 days) exposed to L-MICA-129Met (n=25) or L-MICA-129Val clones (n=25) for 0, 4, and 24 h was analyzed by flow cytometry. The linear regression of MICA expression on target cells and NKG2D expression on NK cells both determined as MFI is shown for the L-MICA-129Met (left panels) and L-MICA-129Val cells (right panels) 4 h (upper panels) and 24 h (lower panels) after co-culture. The coefficient of determination (R^2), the regression coefficient (reg. coeff.) and the *P*-value for Pearson correlation are indicated for both MICA variants.

Appendix Figure S11. Stimulation of NKG2D by MICA-129Met-Fc and MICA-129Val-Fc proteins alone is not sufficient to induce proliferation of $CD8^+$ T cells or IL-2 release. (**A**) MACS-separated $CD8^+$ T cells were cultured in triplicates in 96-well-plates with an immobilized anti-CD3 mAb (1.0 to 0.0 µg/ml). After 72h, 25 % of the supernatant were harvested and IL-2 concentrations were measured by ELISA (see panel B). The harvested medium was replaced by the same volume containing 1 µCi ^{3}H-labeled thymidine. After 12h, the plates were completely harvested and the DNA-bound radioactivity was determined. The SI was calculated by dividing the measured cpm by the cpm of the negative control (only $CD8^+$ T cells). The means and SD of SI determined in 4 independent experiments are displayed. (**B**) The means and SD of IL-2 concentrations determined in triplicates in 4 independent experiments are shown. The anti-CD3 mAb alone can induce proliferation and IL-2 production. (**C**) The means and SD of SI after stimulation with co-stimulatory mAb (anti-CD28, anti-NKG2D), MICA-129Met-Fc, MICA-129Val-Fc proteins, and controls (OVA-Fc, mIgG1, mIgG2a) alone as determined in triplicates in 4 independent experiments are displayed. Thus, these reagents alone did not induce proliferation. (**D**) The means and SD of IL-2 release after stimulation with co-stimulatory mAb (anti-CD28, anti-NKG2D),

MICA-129Met-Fc, MICA-129Val-Fc proteins, and controls (OVA-Fc, mIgG1, mIgG2a) alone as determined in triplicates in 4 independent experiments are shown. Thus, these reagents alone did not induce IL-2 release. (**E**) MACS-separated CFSE-stained $CD8^+$ T cells were stimulated in the same way by anti-CD3, anti-CD28, anti-NKG2D, MICA-129Met-Fc, MICA-129Val-Fc proteins, and controls (OVA-Fc, mIgG1, mIgG2a) alone. The proliferation was assessed at 72h by flow cytometry after gating of $CD3^+CD8^+$ T cells. The MFI of CFSE in unstimulated $CD8^+$ T cells (control) was set to 100 % in individual experiments and the relative decrease due to proliferation was calculated (upper panel). In parallel, the proportion of dividing cells was determined (lower panel). Thus, only the anti-CD3 mAb induced proliferation.

Appendix Figure S12. NKG2D signaling provides a co-stimulatory signal for $CD8^+$ T cells when anti-CD3 is limited to 0.01 or 0.005 µg/ml. (**A**) The threshold of CD3 signaling was determined that requires co-stimulatory signals to induce proliferation and IL-2 release by $CD8^+$ T cells. Purified $CD8^+$ T cells were cultured in triplicates in 96-well-plates coated with an immobilized anti-CD3 in combination with an anti-CD28 mAb. After 72h, 25 % of the supernatant were harvested and IL-2 concentrations were measured by ELISA. The harvested medium was replaced by the same volume containing 1 µCi ^{3}H-labeled thymidine. After 12h, the plates were completely harvested and the DNA-bound radioactivity was determined in triplets. The means and SD of SI and IL-2 release as determined in triplicates in 4 independent experiments are shown (* P<0.05, ** P<0.01, t-test; left panel: anti-CD3 0.01 µg/ml: 1.0 µg/ml anti-CD28 versus 0.0 µg/ml anti-CD28 P=0.0145 and 0.5 µg/ml anti-CD28 versus 0.0 µg/ml anti-CD28 P=0.0002, anti-CD3 0.005 µg/ml: 1.0 µg/ml anti-CD28 versus 0.0 µg/ml anti-CD28 P=0.0048 and 0.5 µg/ml anti-CD28 versus 0.0 µg/ml anti-CD28 P=0.0023; right panel: anti-CD3 0.05 µg/ml: 1.0 µg/ml anti-CD28 versus 0.0 µg/ml anti-CD28 P=0.0091 and 0.5 µg/ml anti-CD28 versus 0.0 µg/ml anti-CD28 P=0.0091, anti-CD3 0.01 µg/ml: 1.0 µg/ml anti-CD28 versus 0.0 µg/ml anti-CD28 P=0.0091 and 0.5 µg/ml anti-CD28 versus 0.0 µg/ml anti-CD28 P=0.0091, anti-CD3 0.005 µg/ml: 1.0 µg/ml anti-CD28 versus 0.0 µg/ml anti-CD28 P=0.0073 and 0.5 µg/ml anti-CD28 versus 0.0 µg/ml anti-CD28 P=0.0073)

(**B**) In parallel, an anti-NKG2D mAb was used for co-stimulation (* $P<0.05$, t-test; left panel: anti-CD3 0.01 µg/ml: 1.0 µg/ml anti-NKG2D versus 0.0 µg/ml anti-NKG2D $P=0.0036$ and 0.5 µg/ml anti-NKG2D versus 0.0 µg/ml anti-NKG2D $P=0.0060$ and 0.1 µg/ml anti-NKG2D versus 0.0 µg/ml anti-NKG2D $P=0.0005$, anti-CD3 0.005 µg/ml: 0.5 µg/ml anti-NKG2D versus 0.0 µg/ml anti-NKG2D $P=0.0309$; right panel: anti-CD3 0.005 µg/ml: 0.5 µg/ml anti-NKG2D versus 0.0 µg/ml anti-NKG2D $P=0.0205$) (**C**) A mIgG$_1$ isotype control was also used in parallel. Thus, NKG2D-mediated co-stimulation was effective when the anti-CD3 mAb was limited (0.01 and 0.005 µg/ml). The anti-NKG2D mAb induced more proliferation and IL-2 release when used in lower concentration (0.5 µg/ml compared to 1.0 µg/ml). Notably, anti-NKG2D co-stimulation induced a proliferation even at 0.1 µg/ml were the anti-CD28 mAb was not able anymore to elicit a significant proliferation.

Appendix Figure S13. Correlation of NKG2D expression on CD8$^+$ T cells with L-MICA-129Val and L-MICA-129Met expression on target cells. (**A**) NKG2D expression on purified CD8$^+$ T cells co-cultured with L-con, L-MICA-129Met (n=19) or L-MICA-129Val clones (n=19) for 24 h was analyzed by flow cytometry. NKG2D expression was analyzed after gating of CD3$^+$CD8$^+$ T cells. The linear regression of MICA expression on target cells and NKG2D expression on CD8$^+$ T cells, both determined as MFI, is displayed for the L-MICA-129Met (left panels) and L-MICA-129Val cells (right panels) 4 h (upper panels) and 24 h (lower panels) after co-culture. The coefficient of determination (R^2), the regression coefficient (reg. coeff.), and the *P*-value for Pearson correlation are indicated for both MICA variants. (**B**) In parallel, the CD8 expression on the CD8$^+$ T cells was determined. The means and SD of the percentage of CD8$^+$ T cells (left panel) and of the MFI of CD8 (right panel) are shown demonstrating the specificity of the effect of MICA engagement for NKG2D expression.

Appendix Figure S14. Impairment of NKG2D-mediated co-stimulation of CD8$^+$ T cells by NKG2D-counter-regulation. Purified CD8$^+$ T cells were cultured on plate-bound anti-NKG2D (1 µg/ml) or an isotype control (IgG$_1$) for 24 h before the NKG2D expression was measured by flow cytometry. These

CD8+ T cells were subsequently stained with CFSE and cultured on plates coated with anti-CD3 mAb (0.005 or 0.01 µg/ml) in combination with anti-CD28 (0.5 µg/ml) mAb as positive control or anti-NKG2D mAb (0.5 µg/ml). Proliferation was measured after 72 h by flow cytometry. The means and SD (n=6) of cell divisions is shown as evaluated in parallel to the MFI (see Fig 7D). Differences between CD8+ T cells pre-exposed to anti-NKG2D and isotype control (mIgG$_1$) were obvious in these experiments (red boxes).

Appendix Figure S15. Characterization of purified IL-2-stimulated NK cells and purified CD8+ T cells. (**A**) NK cell markers (CD16, CD56, CD94, NKp44, NKG2D) and the T cell marker CD3 were always determined by flow cytometry on the surface of MACS-isolated human NK cells, stimulated for 4 days with IL-2 (100 U/ml), before the cells were used in experiments. The means and SD of the percentage of cells positive for the respective markers are shown (n=26). (B) MACS-isolated human CD8+ T cells, were always controlled by flow cytometry before the cells were used in experiments. The means and SD of the percentage of cells positive for the respective marker combinations are shown (n=11).

Appendix Figure S1

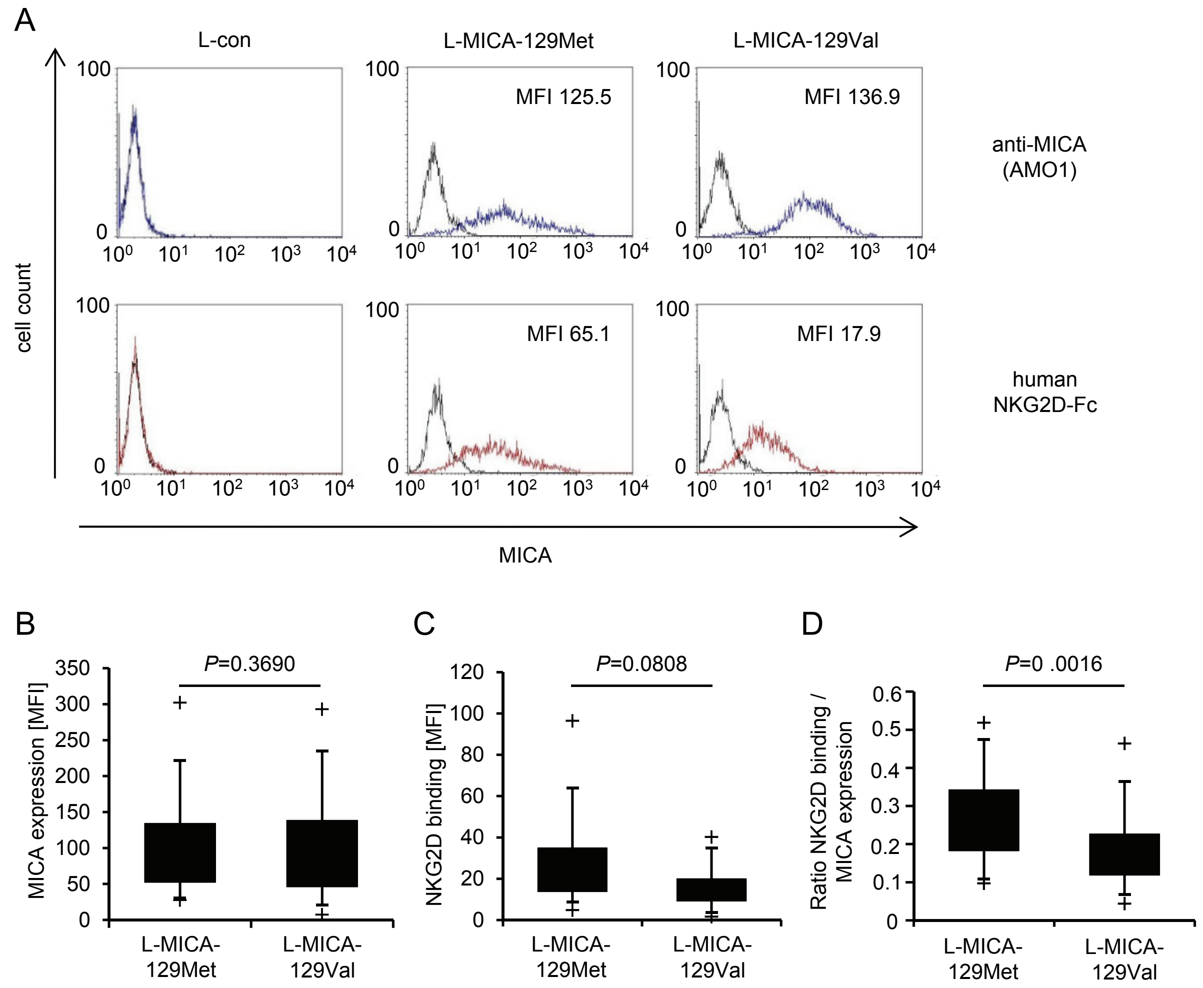

Appendix Figure S2

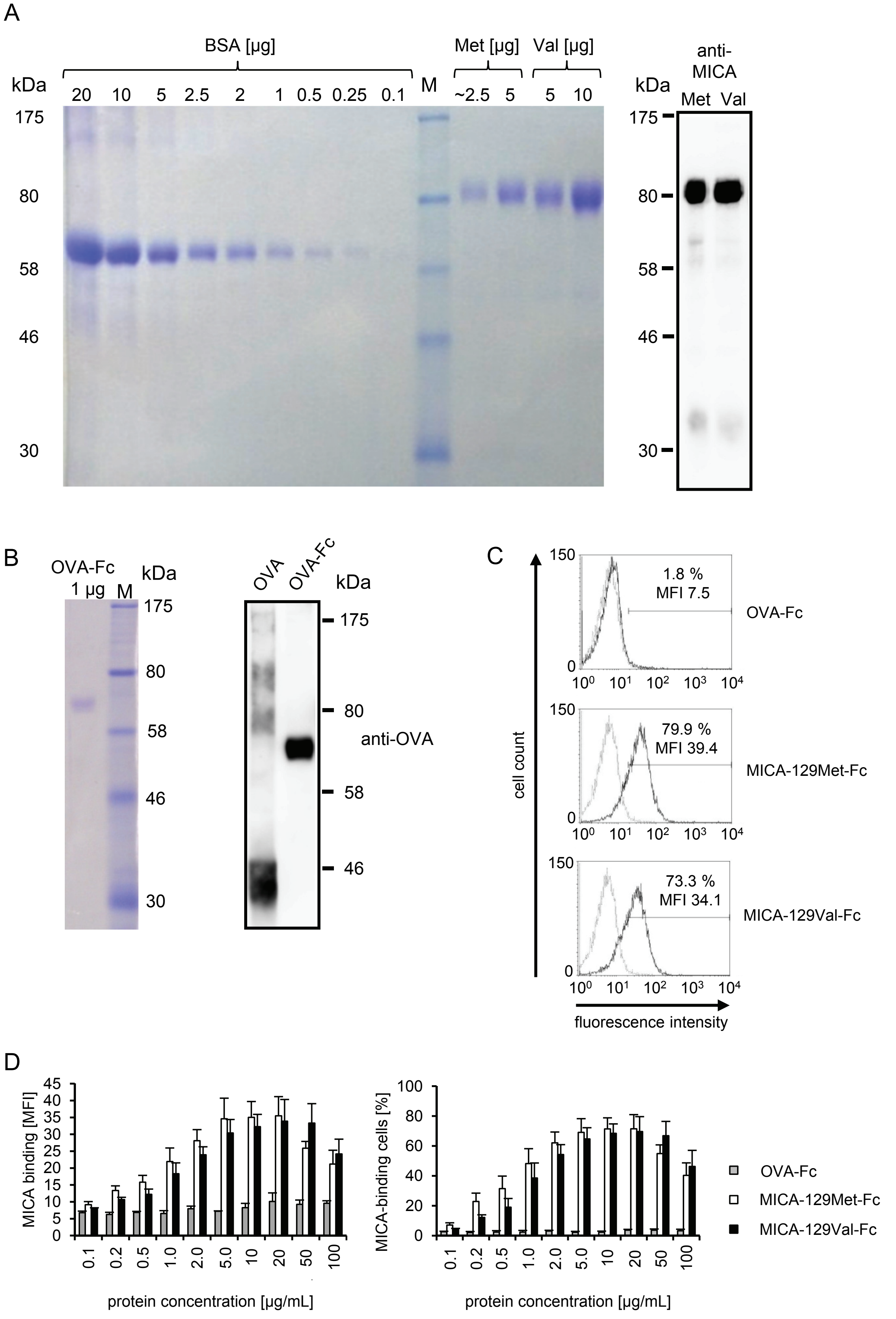

Appendix Figure S3

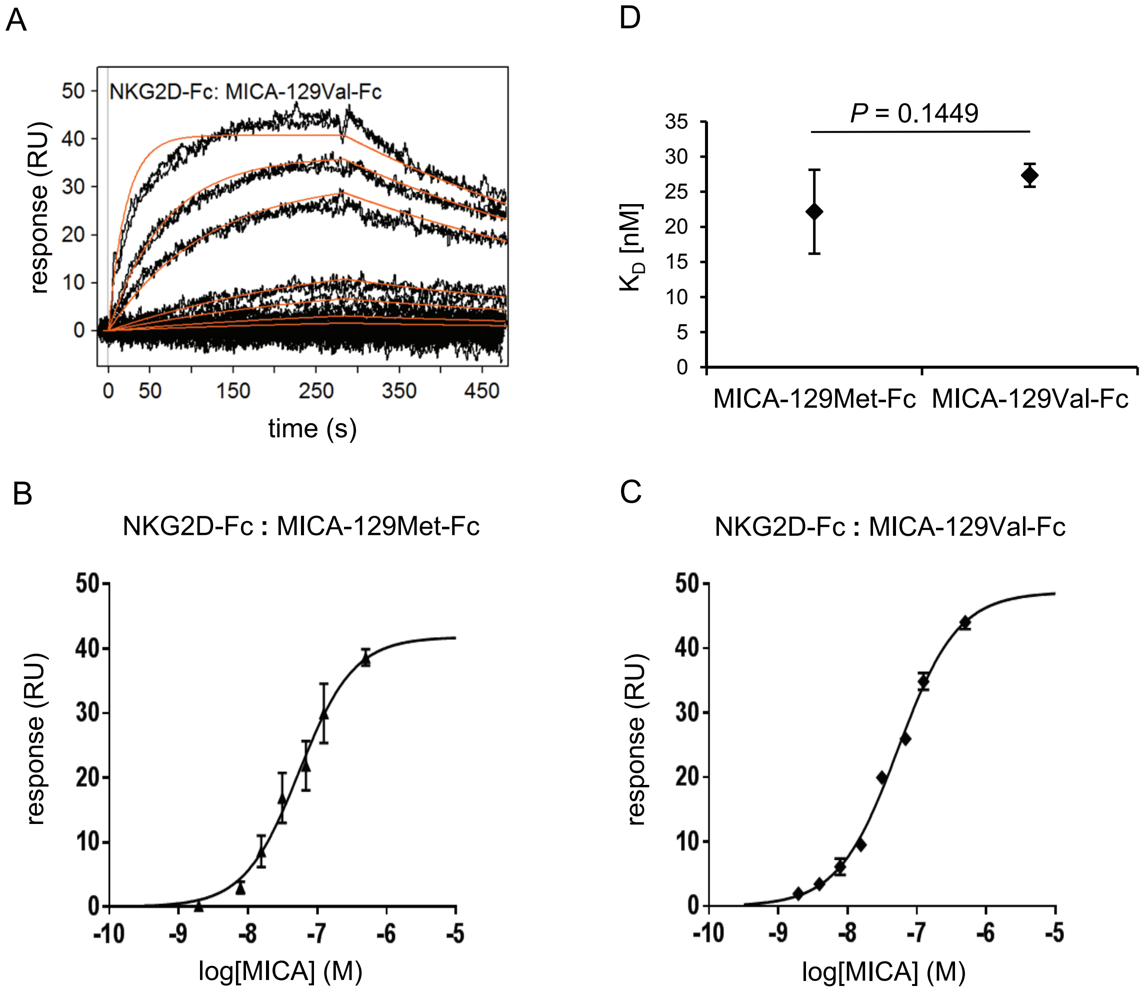

Appendix Figure S4

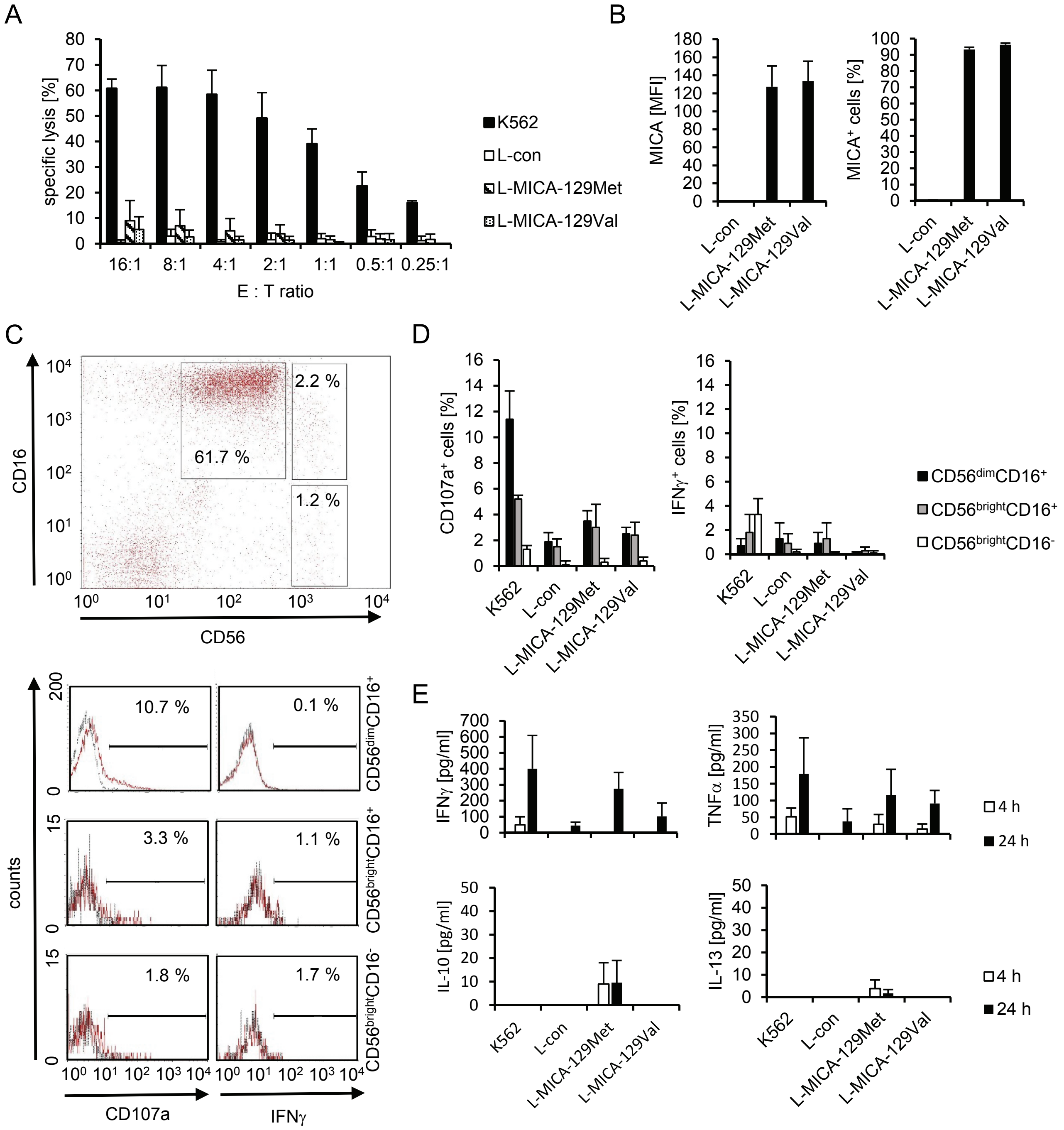

Appendix Figure S5

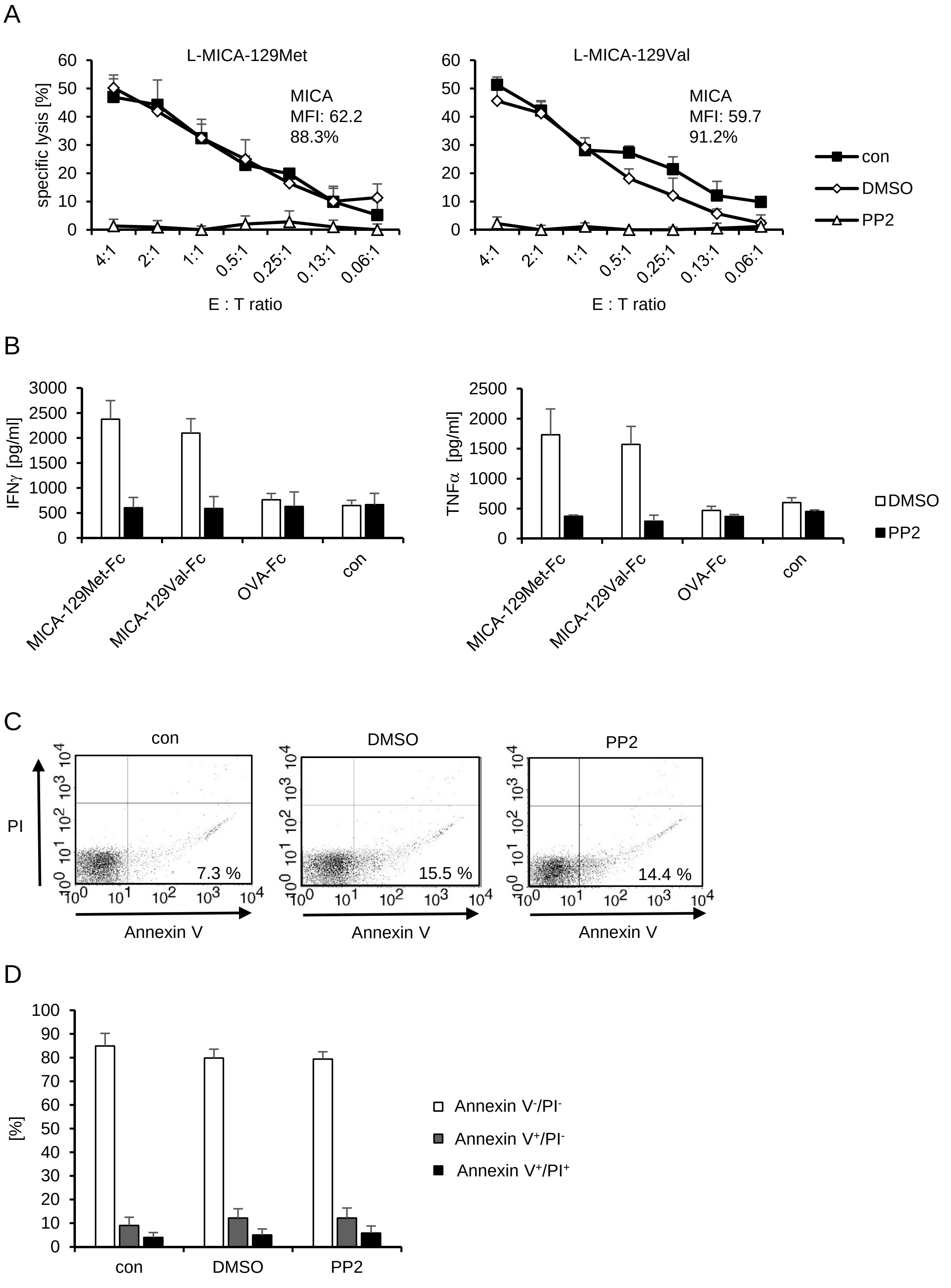

Appendix Figure S6

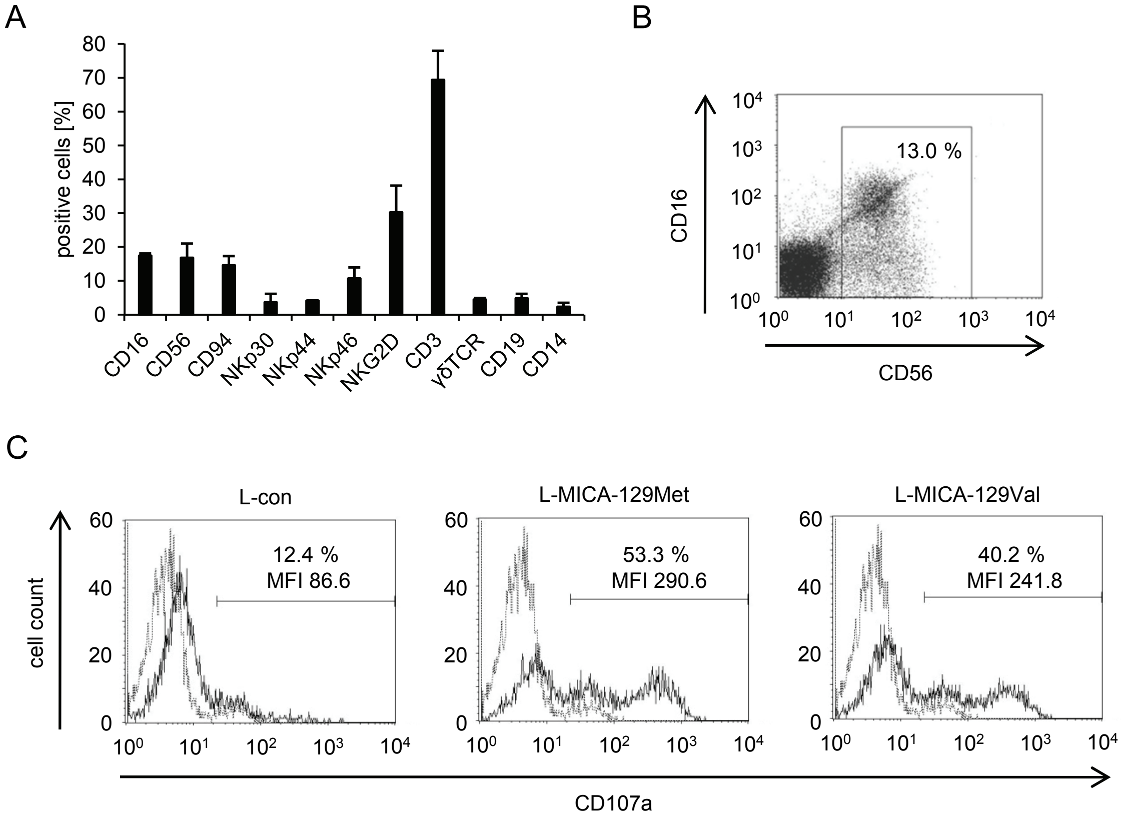

Appendix Figure S7

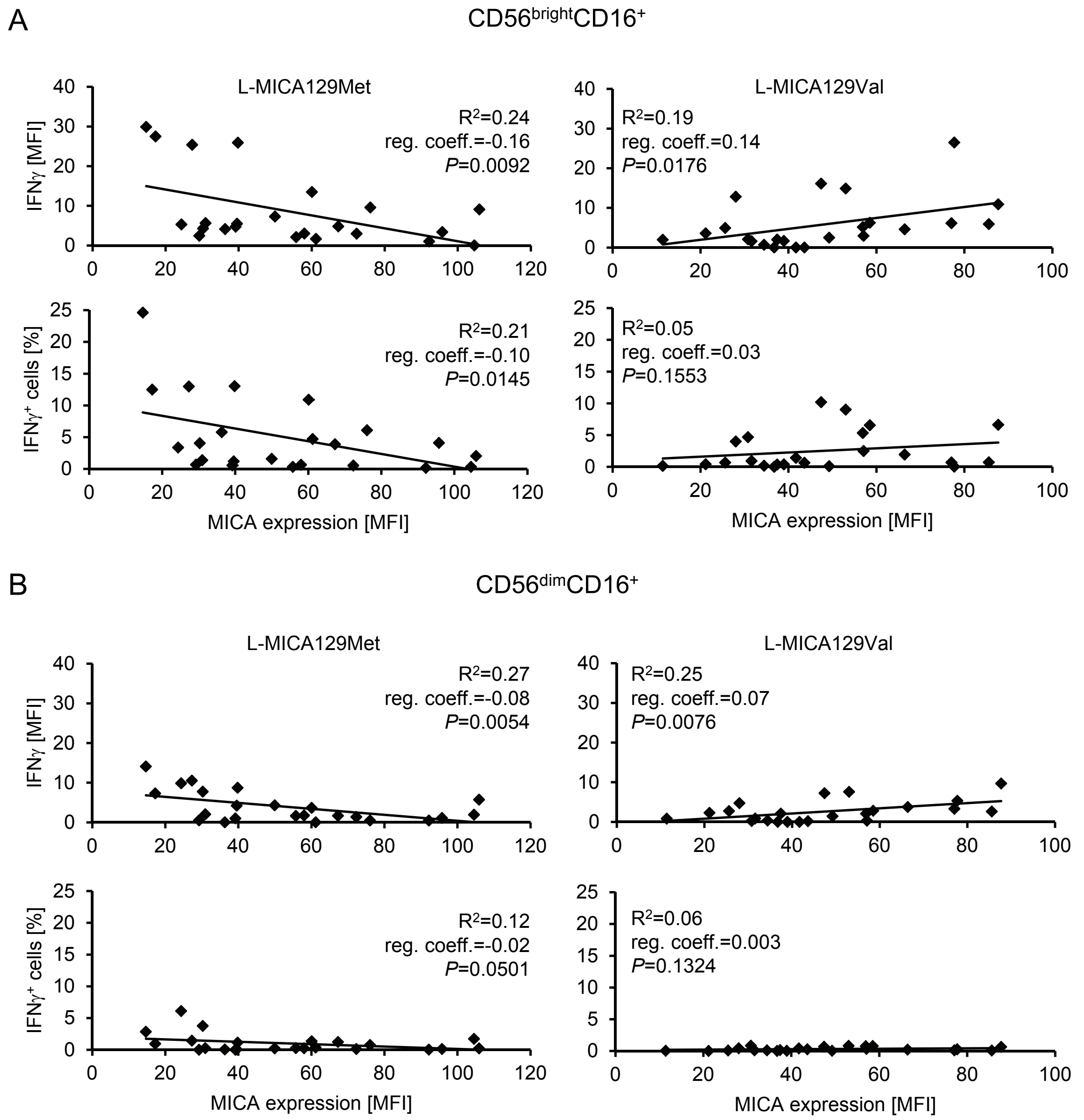

Appendix Figure S8

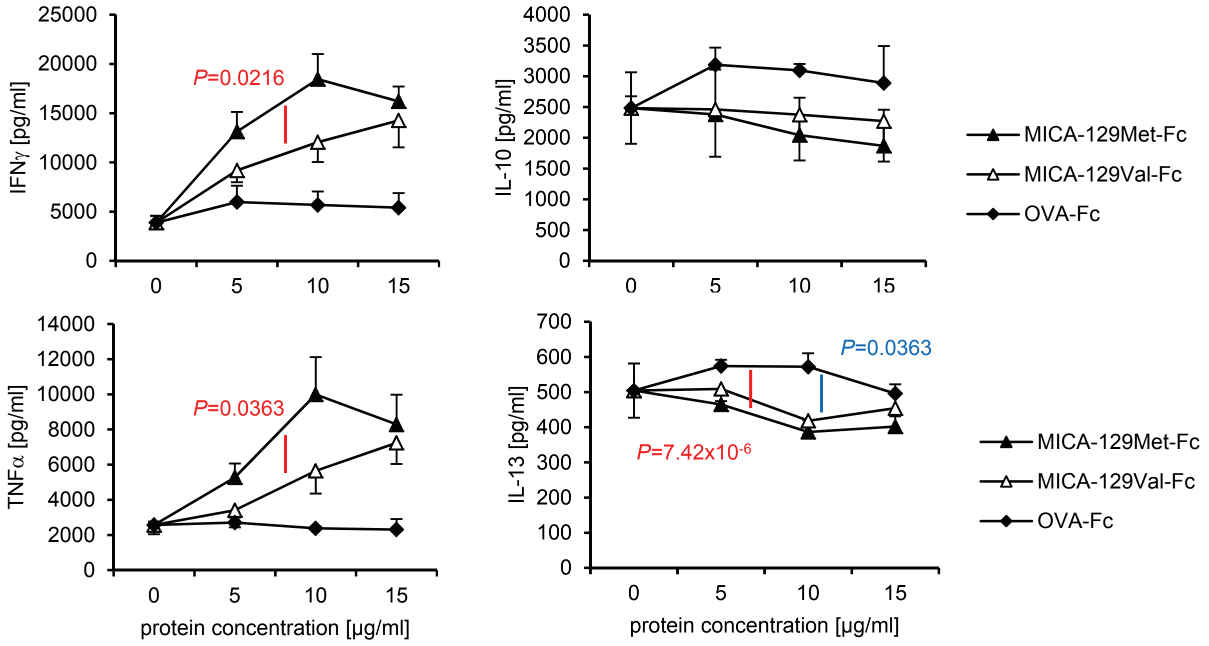

Appendix Figure S9

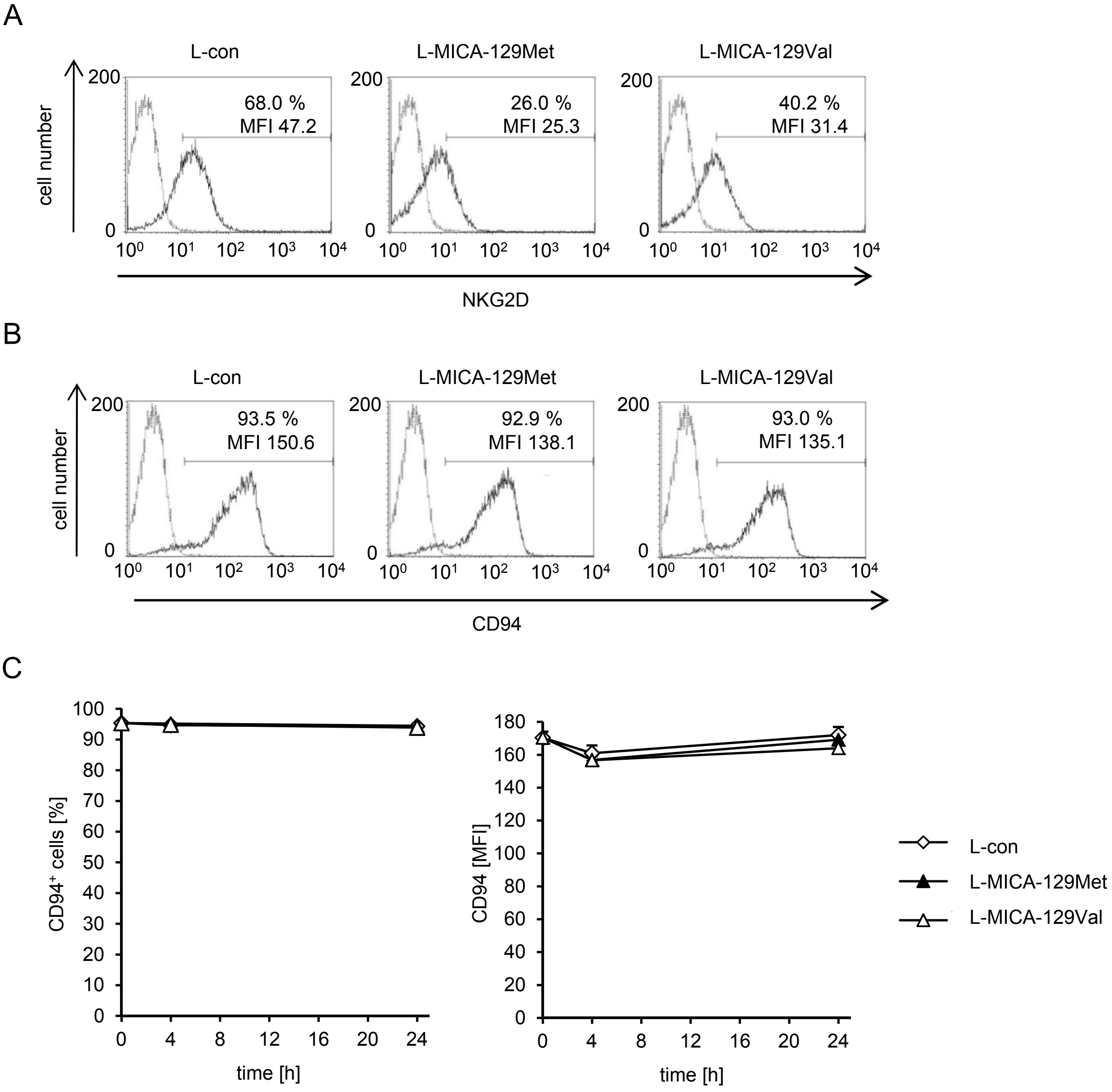

Appendix Figure S10

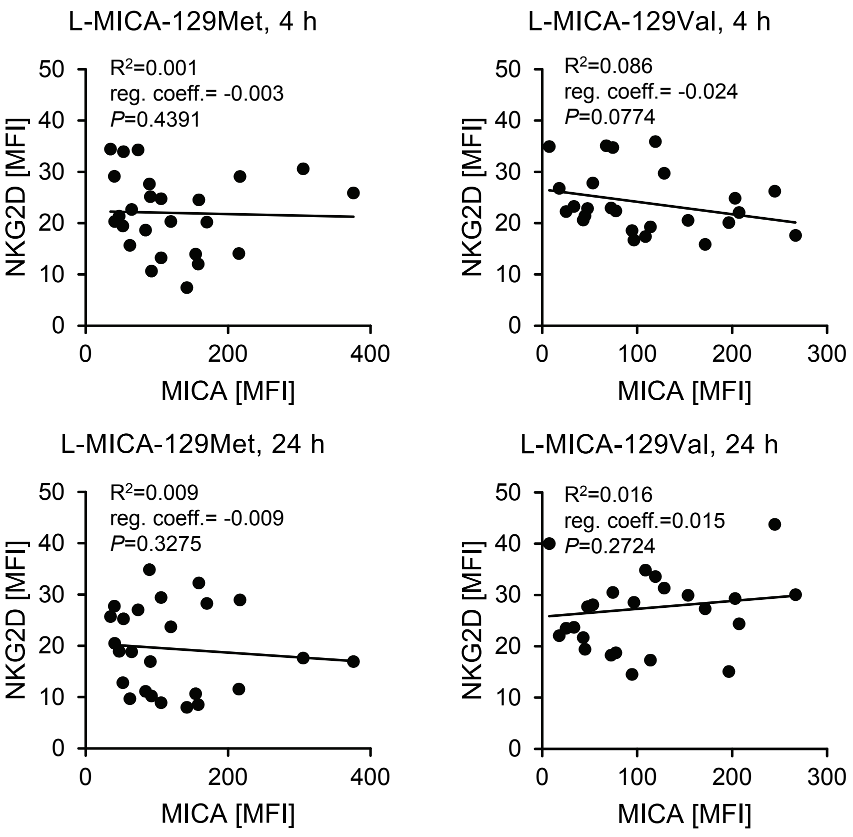

Appendix Figure S11

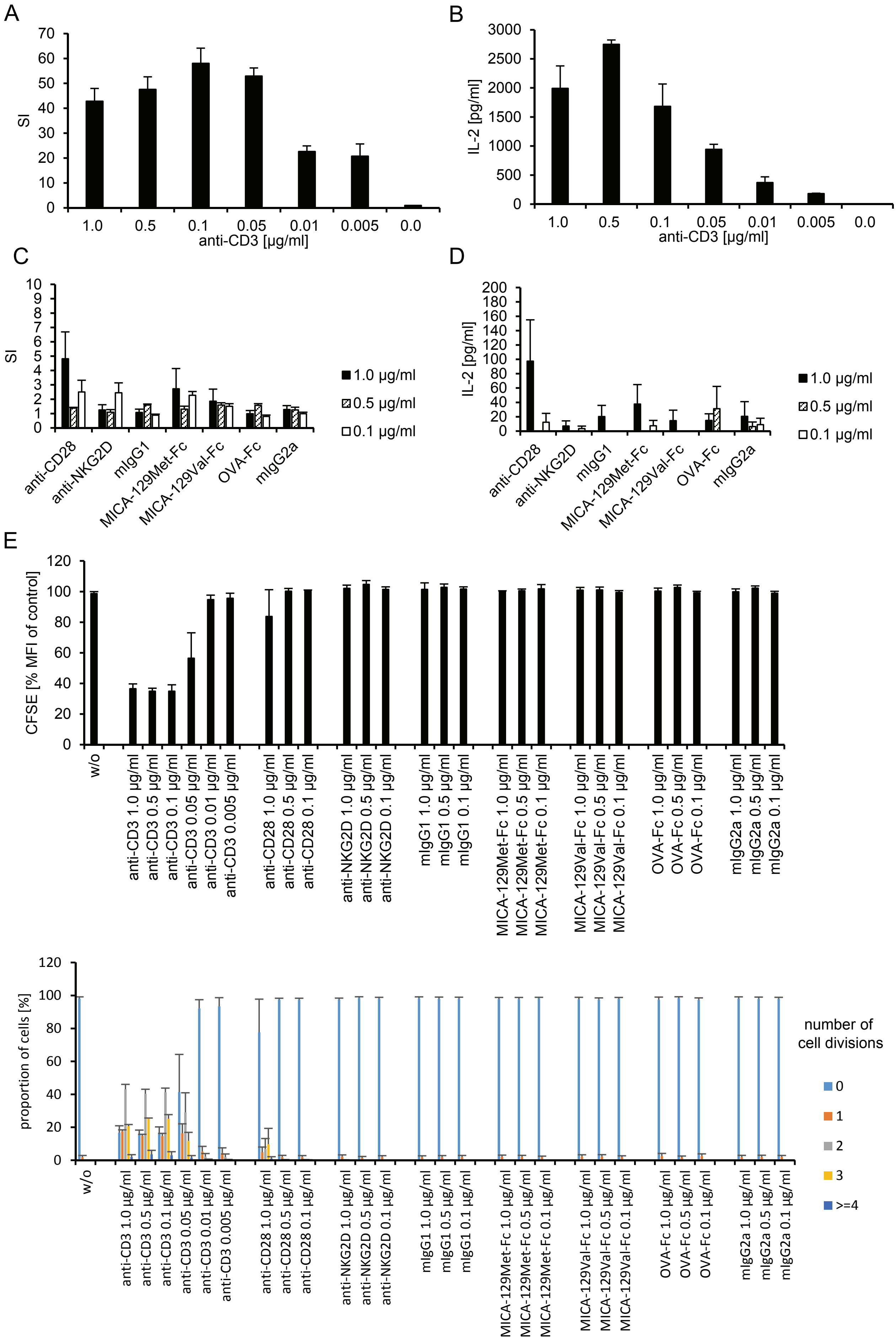

Appendix Figure S12

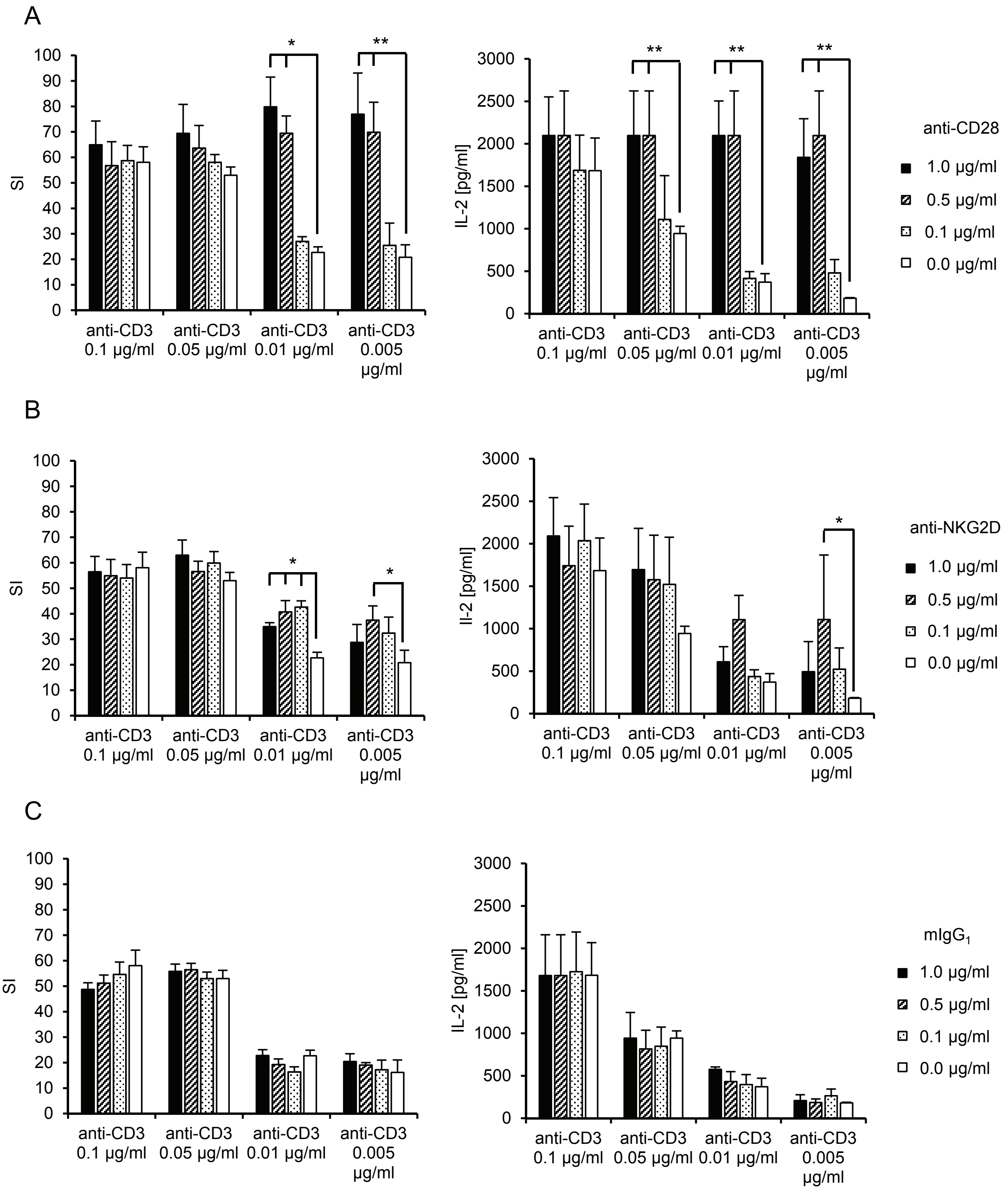

Appendix Figure S13

A

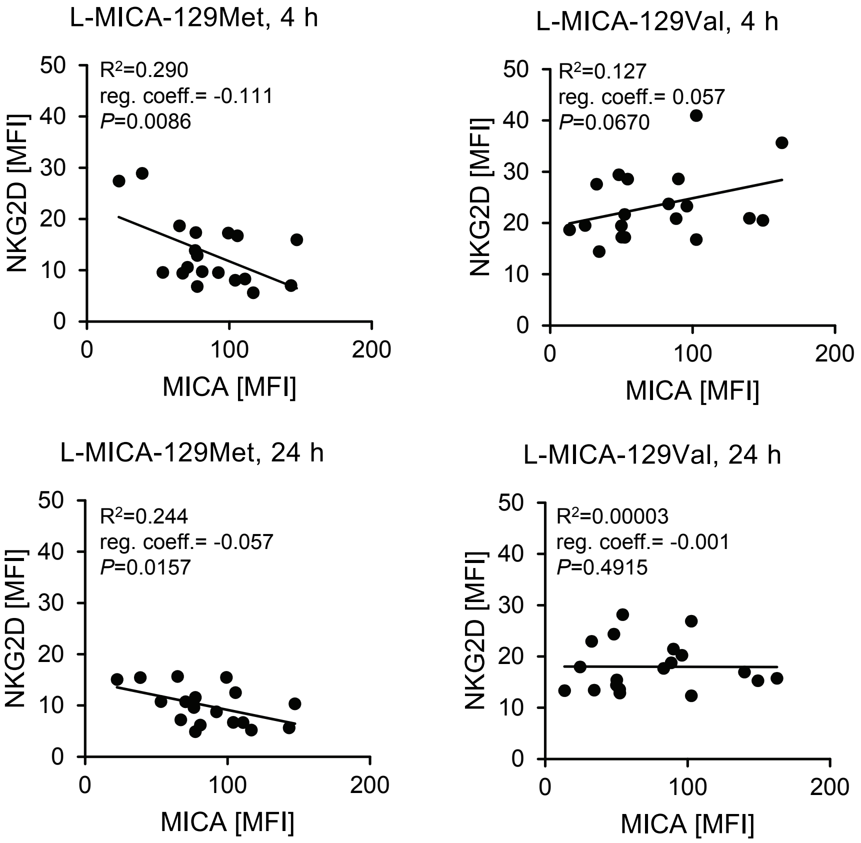

B

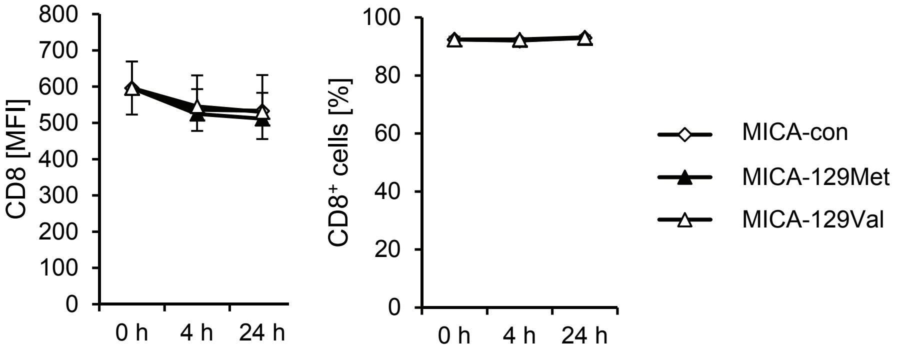

Appendix Figure S14

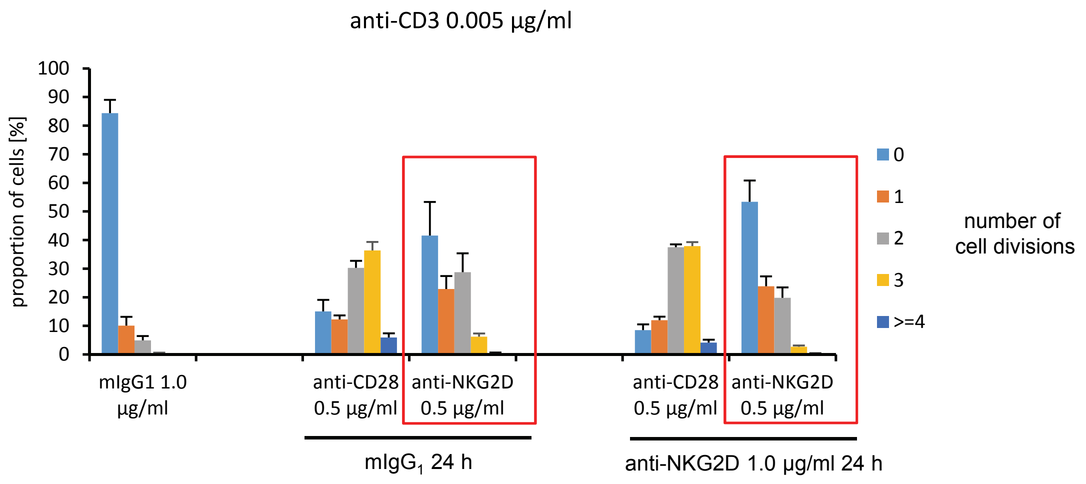

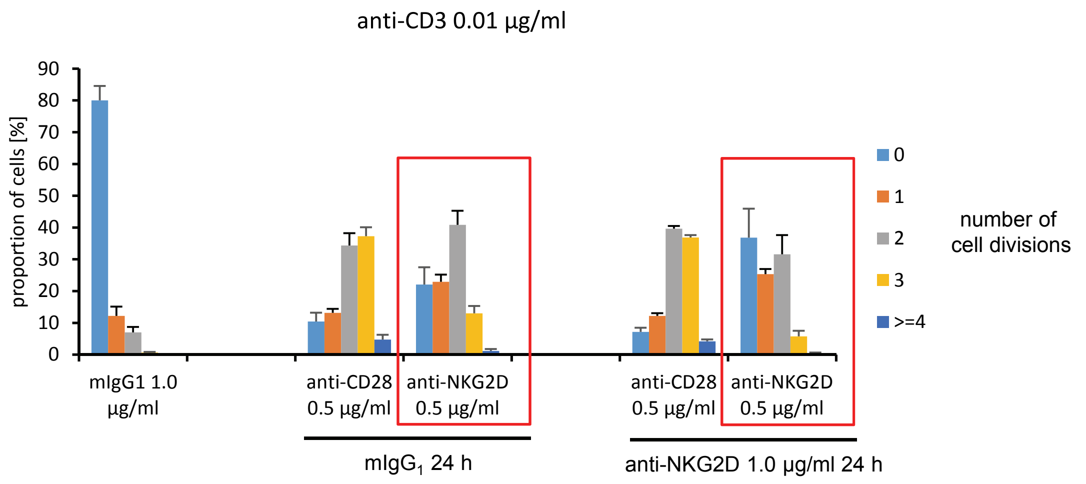

Appendix Figure S15

A

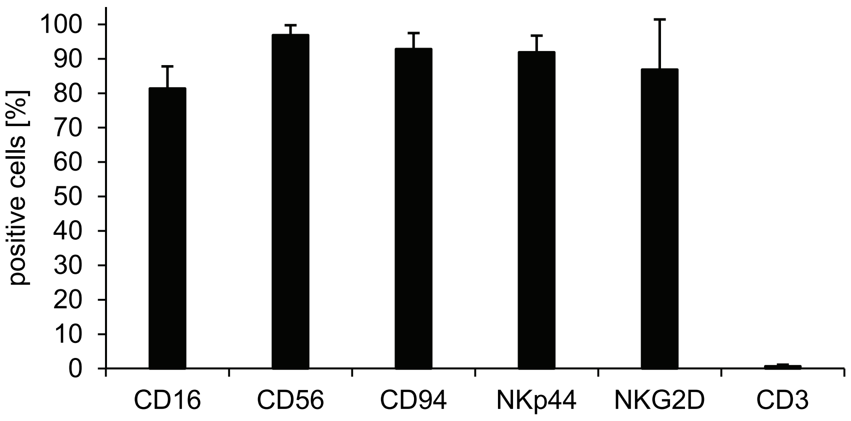

B

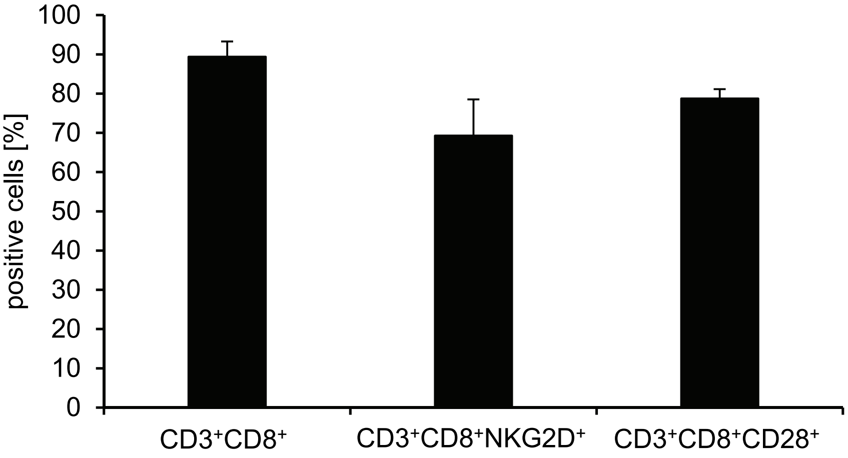

Appendix Table S1. Analysis of MICA shedding from L-MICA-129Met and L-MICA-129Val cells

**cell type**	**n**[1]	**MICA [MFI]** **mean ± SEM**	**sMICA [pg/10^6 cells]** **mean ± SEM**
Ltk$^-$	3	0.8 ± 0.0	0.0 ± 0.0
L-con	3	0.5 ± 0.2	0.0 ± 0.0
L-MICA-129Met	12	83.6 ± 16.9	0.0 ± 0.0
L-MICA-129Val	12	136.1 ± 17.1	0.0 ± 0.0
melanomas	4	13.0 ± 4.6	14.2 ± 8.3
melanomas + SAHA[2]	4	48.4 ± 18.4	79.2 ± 12.7

[1] For the control cells (Ltk$^-$ and L-con) 3 replicates cultured independently at different time point were analyzed, for L-MICA-129Met and L-MICA-129Met 12 independent clones were used, and for melanomas, which served as positive controls, 4 different melanoma cell lines (Sk-Mel-29, Juso, Parl, and Mel Ho) were tested.

[2] The melanoma cell lines were treated with 10 µM suberoylanilide hydroxyamic acid (SAHA, Qbiogene-Alexis, Grünberg, Germany) for 20 h before analysis to increase MICA expression as described previously (Elsner L, Flügge PF, Lozano J, Muppala V, Eiz-Vesper B, Demiroglu SY, Malzahn D, Herrmann T, Brunner E, Bickeböller H, Multhoff G, Walter L, Dressel R (2010) The endogenous danger signals HSP70 and MICA cooperate in the activation of cytotoxic effector functions of NK cells. *J Cell Mol Med* 14: 992-1002).

Appendix Table S2. Antibodies used in the study

Antigen	Isotype	Clone	Label	Supplier
CD3	mouse IgG_{2a}	MEM 57	FITC	ImmunoTools, Friesoythe, Germany
CD3	mouse IgG_{2a}	HIT3a	PE	BioLegend, Fell, Germany
CD3	mouse IgG_1	UCHT1 (LEAF)	-	BioLegend, Fell, Germany
CD8	mouse IgG_1	HIT8a	PE/Cy5	BioLegend, Fell, Germany
CD14	mouse IgG_{2a}	Tük4	PE	Caltag Laboratories, Hamburg, Germany
CD16	mouse IgG_1	3G8	PE/Cy5	BioLegend, Fell, Germany
CD19	mouse IgG_1	HIB19	PE	BioLegend, Fell, Germany
CD28	mouse IgG_1	CD28.2	PE	BioLegend, Fell, Germany
CD28	mouse IgG_1	CD28.2 (LEAF)	-	BioLegend, Fell, Germany
CD56	mouse IgG_1	HCD56	FITC	BioLegend, Fell, Germany
CD56	mouse IgG_1	HCD56	PE	BioLegend, Fell, Germany
CD94	mouse IgG_1	HP-3D9	FITC	Becton Dickinson, Heidelberg, Germany
CD107a (LAMP-1)	mouse IgG_1	H4A3	FITC	BioLegend, Fell, Germany
CD314 (NKG2D)	mouse IgG_1	149810	PE	R&D Systems, Wiesbaden, Germany
CD314 (NKG2D)	mouse IgG_1	149810	-	R&D Systems, Wiesbaden, Germany
CD335 (NKp46)	mouse IgG_1	9E2	PE	BioLegend, Fell, Germany
CD336 (NKp44)	mouse IgG_1	P44-8	APC	BioLegend, Fell, Germany
CD337 (NKp30)	mouse IgG_1	P30-15	PE	BioLegend, Fell, Germany
$\gamma\delta$TCR	mouse IgG_{2a}	B1	PE	BioLegend, Fell, Germany
β-actin	mouse IgG_1	AC-15	-	Sigma-Aldrich, Taufkirchen, Germany
IFNγ	mouse IgG_1	4S.B3	PE	BioLegend, Fell, Germany
MICA	mouse IgG_1	AMO1	-	Bamomab, Gräfelfing , Germany
MICA	goat IgG	polyclonal (BAF1300)	Biotin	R&D Systems, Wiesbaden, Germany

Ovalbumin (OVA)	mouse IgG_1	OVA-14	-	Sigma-Aldrich, Taufkirchen, Germany
phospho-Tyr	mouse IgG_{2b}	4G10	-	Merck Millipore, Darmstadt, Germany
phospho-SRC family (Tyr419)	rabbit IgG	polyclonal (#2101)	-	Cell Signaling Technology, Danvers, MA, USA
mouse IgG	goat IgG	polyclonal (115-005-003)	-	Jackson Laboratories, via Dianova, Hamburg, Germany
mouse IgG	goat IgG F(ab')2 fragment	polyclonal (115-006-062)	-	Jackson Laboratories, via Dianova, Hamburg, Germany
mouse IgG	goat IgG	polyclonal (155-095-062)	FITC	Jackson Laboratories, via Dianova, Hamburg, Germany
mouse IgG	goat IgG	polyclonal (115-035-003)	HRP	Jackson Laboratories, via Dianova, Hamburg, Germany
human IgG	goat IgG	polyclonal (109-095-098)	FITC	Jackson Laboratories, via Dianova, Hamburg, Germany
rabbit IgG	goat IgG	polyclonal (#31430)	HRP	Thermo Fisher Scientific, Bonn, Germany

The following abbreviations are used: APC allophycocyanin; Cy5, Cyanine 5; FITC, fluorescein isothiocyanate; HPR, horseradish peroxidase; LEAF, low endotoxin azide-free; PE, phycoerythrin. The phospho-SRC family (Tyr419) is also known as Tyr416 when following the original nomenclature from chicken.
