# Supplementary material for: The MICA-129 dimorphism affects NKG2D signaling and outcome of hematopoietic stem cell transplantation
Source: EMBO Mol Med. 2015 Oct 19;7(11):1480–502. doi: 10.15252/emmm.201505246 (PMC4644379; doi:10.15252/emmm.201505246)
Supplement: Supplementary file 2 [file emmm0007-1480-sd2.pdf]

Expanded View Figures

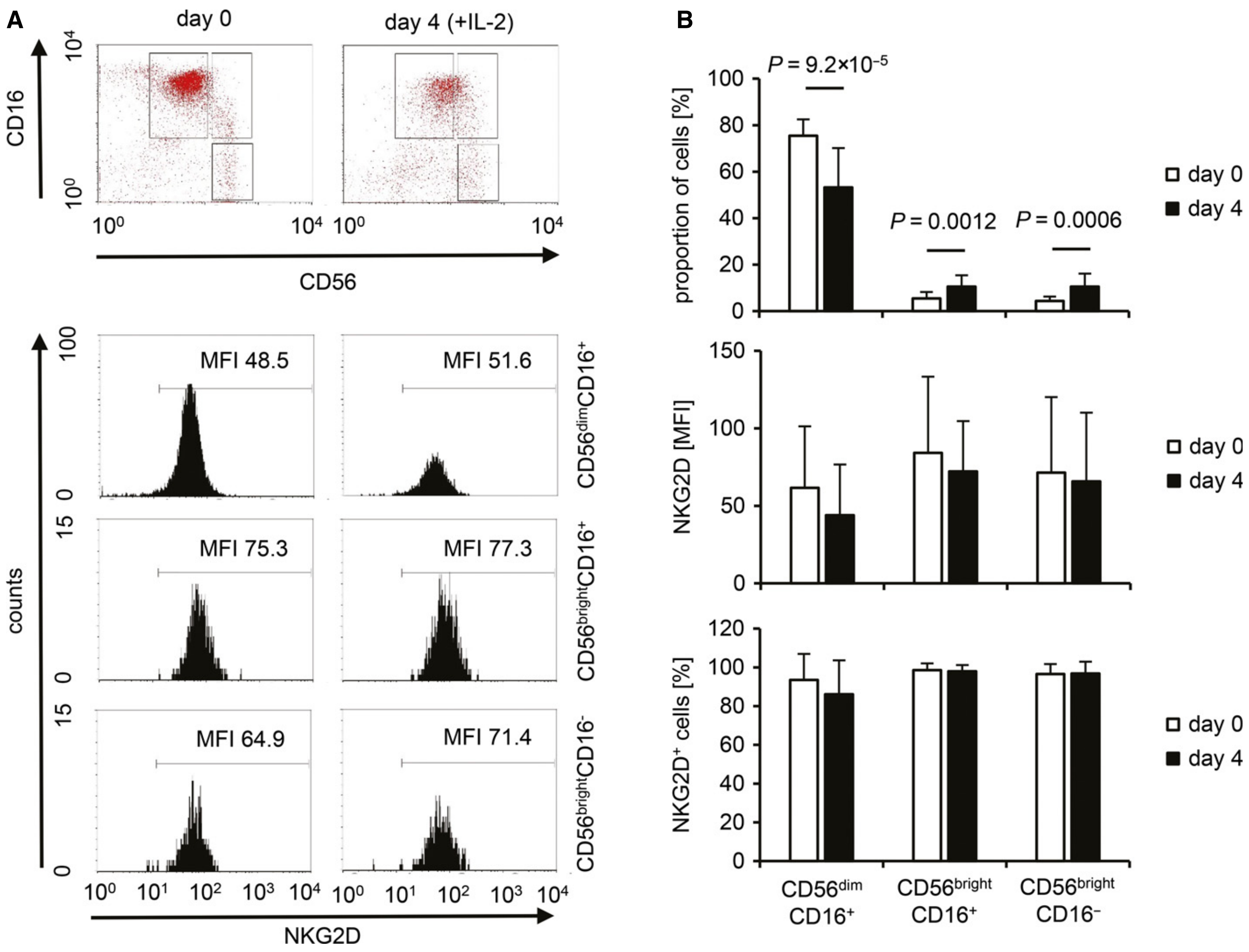

Figure EV1. NKG2D expression on freshly isolated and IL-2-stimulated NK-cell populations.

A Three populations ($CD56^{dim}CD16^{+}$, $CD56^{bright}CD16^{+}$, $CD56^{bright}CD16^{-}$) were gated on freshly isolated NK cells (day 0) and NK cells stimulated for 4 days with IL-2 (100 U/ml) as exemplified in the upper panel. The NKG2D expression on these NK-cell populations is displayed in the lower panel, and the MFI for NKG2D is indicated.

B Summaries (means + SD) of the proportion of the three NK-cell populations and their NKG2D expression intensity (MFI and percentage $NKG2D^{+}$ cells) at day 0 (n = 21) and 4 days after IL-2 stimulation (n = 16) are shown. The data were analyzed by t-test, and the P-values of significant differences are indicated.

 © 2015 The Authors

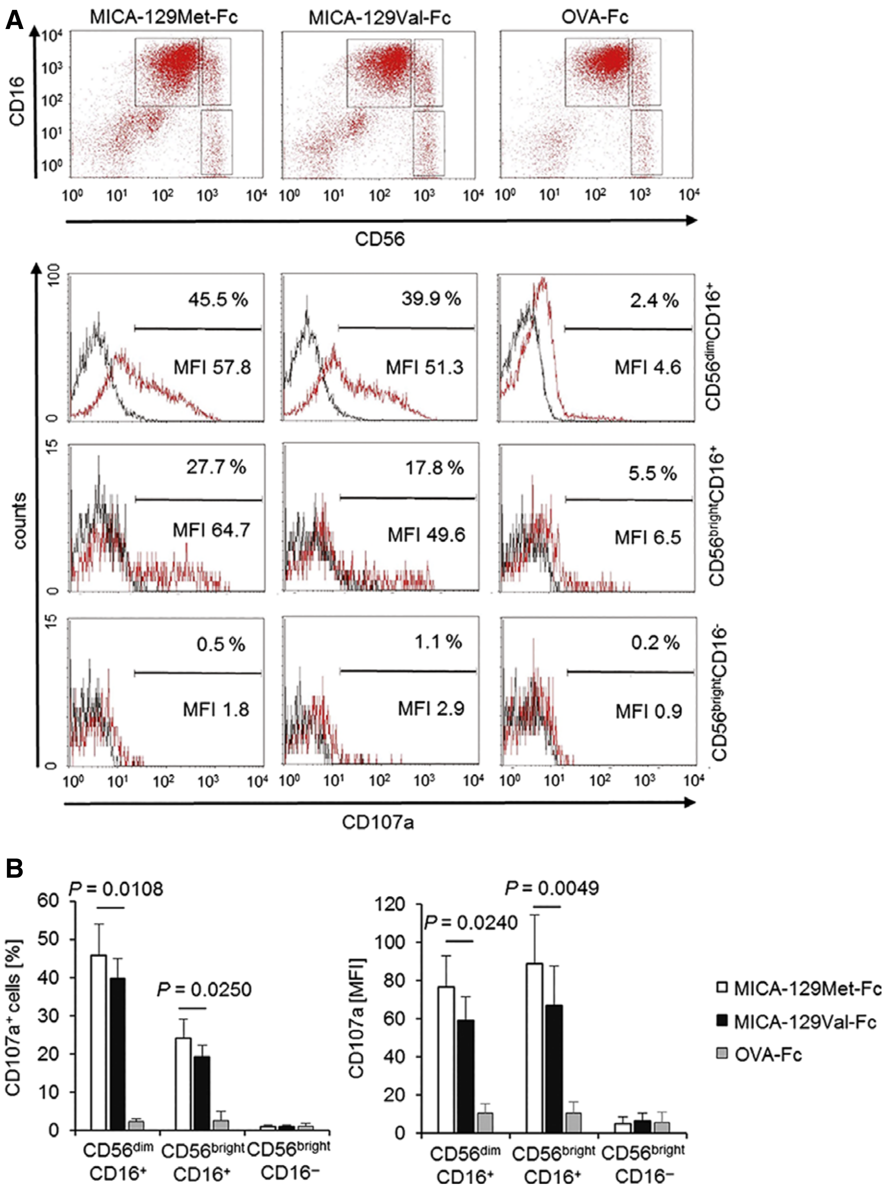

Figure EV2. Degranulation of CD56^{dim}CD16^{+} and CD56^{bright}CD16^{+} NK cells in response to the MICA-129Met and the MICA-129Val isoforms.

A Purified IL-2-stimulated NK cells (100 U/ml for 4 days) were cultured for 2 h on plates coated with MICA-129Met-Fc, MICA-129Val-Fc, or OVA-Fc proteins (10 μg/ml) before flow cytometry. The NK-cell populations were gated as illustrated in the upper panel (CD56^{dim}CD16^{+}, CD56^{bright}CD16^{+}, CD56^{bright}CD16^{-}), and CD107a expression was determined as displayed in the lower panel (red: CD107a, black: isotype control). The specific MFI (MFI CD107a minus MFI isotype control) and the percentage of CD107a^{+} cells are indicated.

B A summary (means + SD) of 5 experiments is shown. The data were analyzed by *t*-test, and the *P*-values of significant differences are indicated.

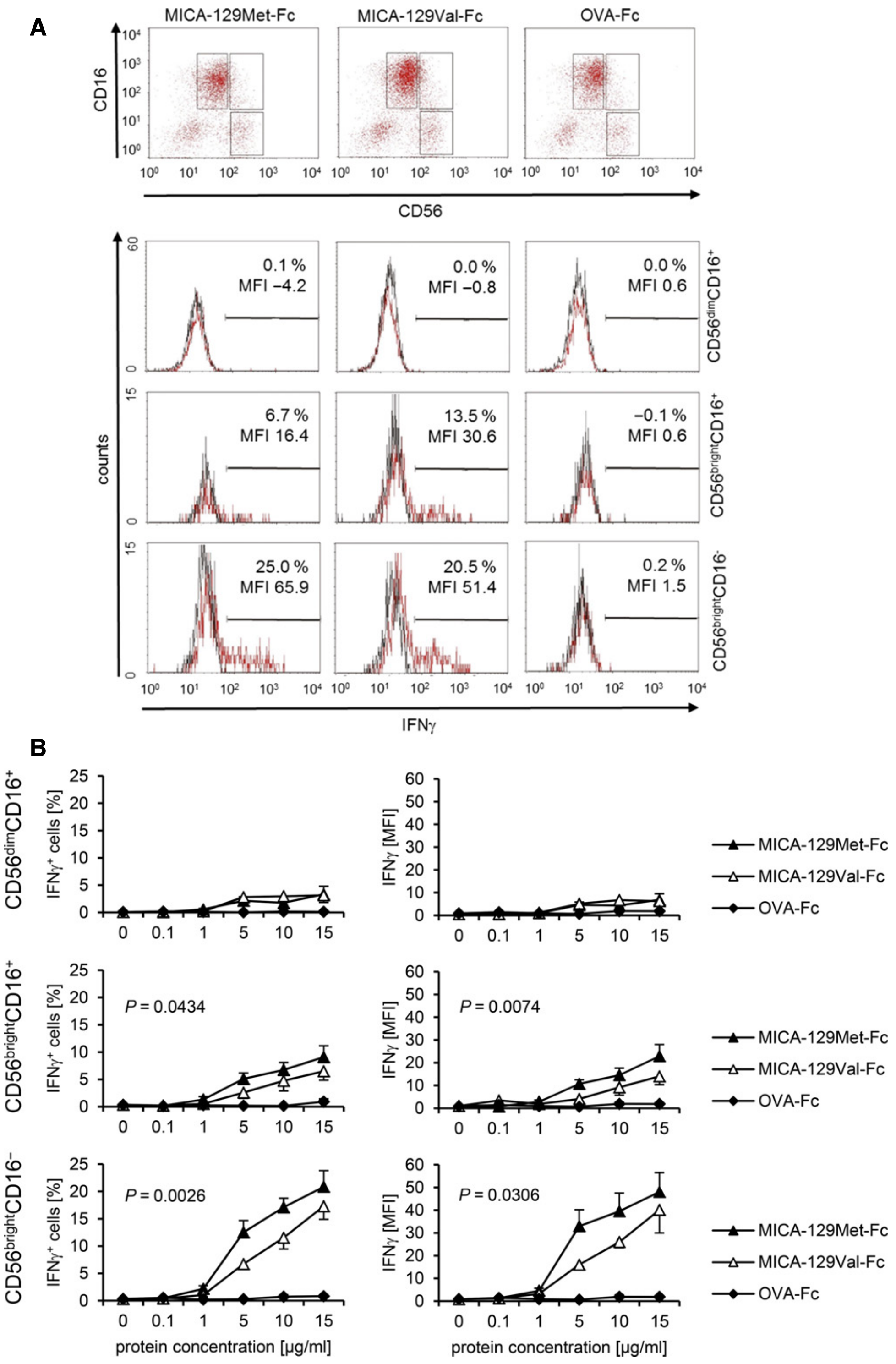

Figure EV3. Expression of IFNγ in CD56^{bright}CD16^{-} and CD56^{bright}CD16^{+} NK cells in response to the MICA-129Met and the MICA-129Val isoforms.

A Purified IL-2-stimulated NK cells (100 U/ml for 4 days) were cultured for 4 h on plates coated with MICA-129Met-Fc, MICA-129Val-Fc, or OVA-Fc proteins (0, 0.1, 1, 5, 10, 15 μg/ml) before flow cytometry. The NK-cell populations were gated as exemplified in the upper panel (CD56^{dim}CD16^{+}, CD56^{bright}CD16^{+}, CD56^{bright}CD16^{-}). The intracellular IFNγ expression in these populations is displayed in the lower panel (red: IFNγ, black: isotype control). The specific MFI (MFI IFNγ minus MFI isotype control) and the percentage of IFNγ^{+} cells are indicated.

B A summary (means + SEM) of 9 experiments is shown. The data were analyzed by two-way ANCOVA adjusted for the protein concentration (5, 10, 15 μg/ml), and the *P*-values of significant differences between the MICA-129Met-Fc and MICA-129Val-Fc proteins are indicated.

 © 2015 The Authors

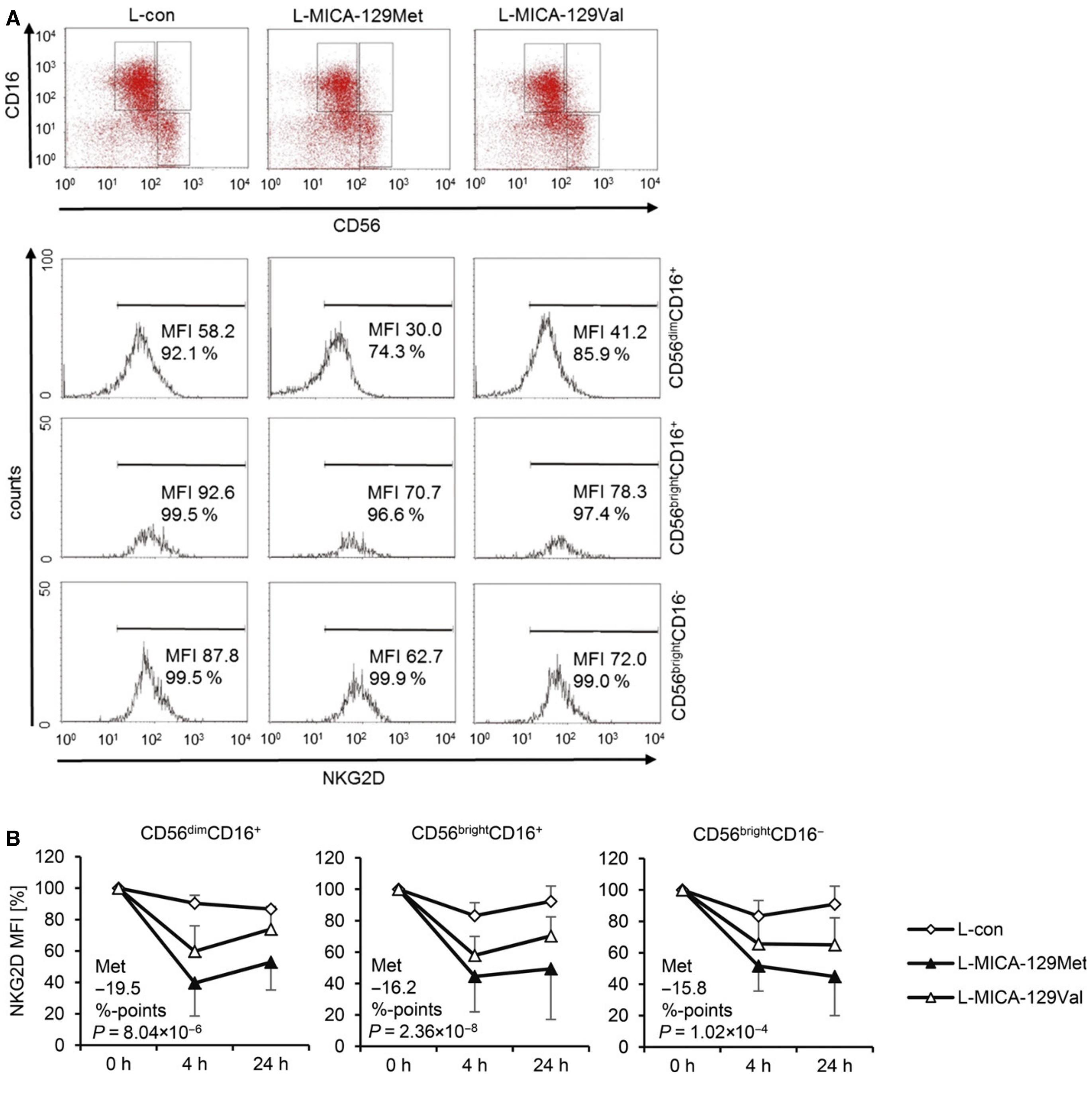

Figure EV4. Down-regulation of NGK2D on CD56^{dim}CD16^{+}, CD56^{bright}CD16^{+}, and CD56^{bright}CD16^{-} NK cells in response to the MICA-129Met and the MICA-129Val isoforms.

A Purified IL-2-stiumlated NK cells (100 U/ml for 4 days) were co-cultured with L-con, L-MICA-129Met, or L-MICA-129Val clones for 4 h. Three NK-cell populations (CD56^{dim}CD16^{+}, CD56^{bright}CD16^{+}, CD56^{bright}CD16^{-}) were gated as illustrated in the upper panel. The NKG2D expression on these NK-cell populations is displayed in the lower panel, and the MFI of NKG2D and the percentages of NKG2D^{+} cells are indicated.

B A summary (means + SD) of NKG2D expression on the three NK-cell populations 4 and 24 h after co-culture with L-con (n = 3), L-MICA-129Met (n = 17), and L-MICA-129Val clones (n = 18) is displayed. The NKG2D expression (MFI) at the beginning (0 h) was set to 100%. The average reduction of NKG2D on NK cells in response to L-MICA-129Met compared to L-MICA-129Val clones at 4 and 24 h (%-points) is indicated in the panels. The differences were analyzed by repeated measures ANOVA, and P-values are indicated.

© 2015 The Authors

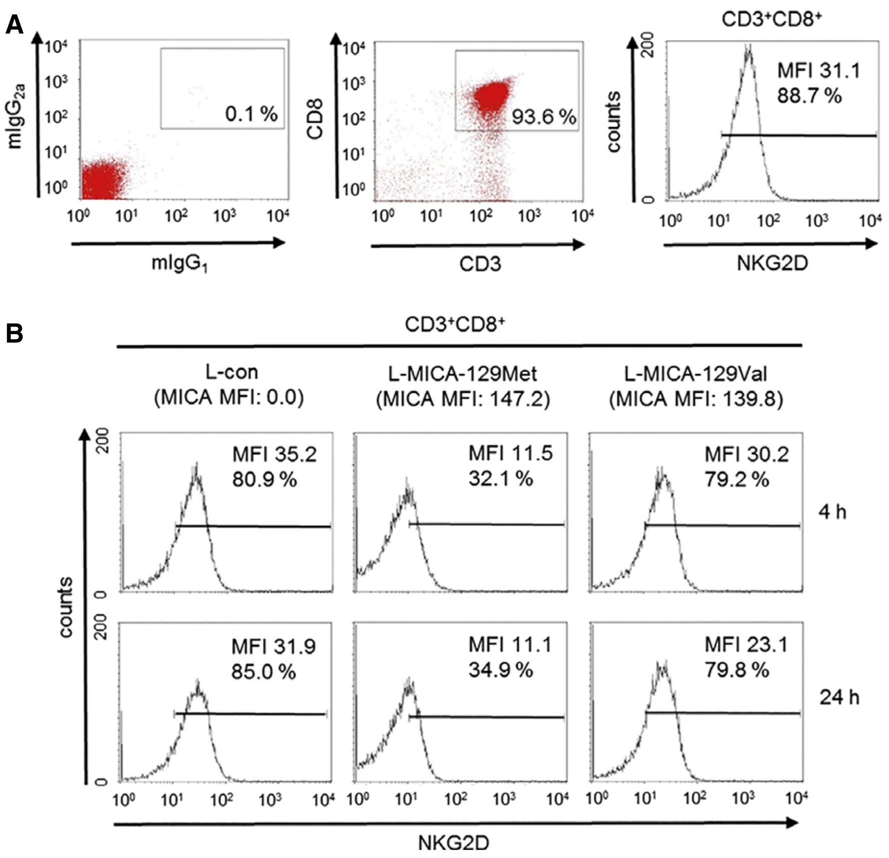

Figure EV5. Down-regulation of NGK2D on CD8$^+$ T cells in response to the MICA-129Met and MICA-129Val isoforms.

A Purified CD8$^+$ T cells were analyzed by flow cytometry for NKG2D expression as illustrated here. The MFI for NKG2D and the percentage of NKG2D$^+$CD8$^+$ T cells are indicated.

B The NK cells were subsequently co-cultured with an L-con, L-MICA-129Met, or L-MICA-129Val clone (the MFI values for MICA on these clones are indicated above the histograms in brackets). NKG2D expression was determined as illustrated in (A) after 4 and 24 h. The MFI for NKG2D and the percentages of NKG2D$^+$CD8$^+$ T cells are indicated.

 © 2015 The Authors
