# Supplementary material for: The MICA-129 dimorphism affects NKG2D signaling and outcome of hematopoietic stem cell transplantation
Source: EMBO Mol Med. 2015 Oct 19;7(11):1480–502. doi: 10.15252/emmm.201505246 (PMC4644379; doi:10.15252/emmm.201505246)
Supplement: Supplementary file 3 [file emmm0007-1480-sd3.pdf]

EMBO
Molecular Medicine

The MICA-129 dimorphism affects NKG2D signaling and outcome of hematopoietic stem cell transplantation

Antje Isernhagen, Dörthe Malzahn, Elena Viktorova, Leslie Elsner, Sebastian Monecke, Frederike von Bonin, Markus Kilisch, Janne Marieke Wermuth, Neele Walther, Yesilda Balavarca, Christiane Stahl-Hennig, Michael Engelke, Lutz Walter, Heike Bickeböller, Dieter Kube, Gerald Wulf, & Ralf Dressel

Corresponding author: Ralf Dressel, University Medical Center Gˆttingen

Review timeline:

Submission date:	14 March 2015
Editorial Decision:	21 April 2015
Revision received:	20 July 2015
Editorial Decision:	11 September 2015
Revision received:	15 September 2015
Accepted:	17 September 2015

Transaction Report:

(Note: With the exception of the correction of typographical or spelling errors that could be a source of ambiguity, letters and reports are not edited. The original formatting of letters and referee reports may not be reflected in this compilation.)

Editor: Céline Carret

1st Editorial Decision 21 April 2015

Thank you for the submission of your manuscript to EMBO Molecular Medicine. We have now heard back from the three referees whom we asked to evaluate your manuscript.

You will see below that all three referees are rather enthusiastic about your article, although referees 1 and 2 do have suggestions to further improve conclusiveness and strengthen the study.

Should you be able to address these criticisms in full, we would be happy to consider a revised manuscript.

Please note that it is EMBO Molecular Medicine policy to allow only a single round of revision and that, as acceptance or rejection of the manuscript will depend on another round of review, your responses should be as complete as possible.

EMBO Molecular Medicine has a "scooping protection" policy, whereby similar findings that are published by others during review or revision are not a criterion for rejection. Should you decide to submit a revised version, I do ask that you get in touch after three months if you have not completed it, to update us on the status.

Please also contact us as soon as possible if similar work is published elsewhere. If other work is published we may not be able to extend the revision period beyond three months.

I look forward to receiving your revised manuscript.

***** Reviewer's comments *****

Referee #1 (Comments on Novelty/Model System):

These findings are extremely interesting, especially when looking at the correlation of the survival curves with MICA-129 dimorphisms. The findings suggest that analysis for MICA-129met may have diagnostic potential for predicting survivability from aGVHD following HSC transplantation.

Referee #1 (Remarks):

In the manuscript entitled "The MICA-129 dimorphism affects NKG2D signaling and outcome of hematopoietic stem cell transplantation," the authors present correlative data that seem to show better survival outcomes in patients that show the presence of the MICA-129met versus MIVA-129val following HSC transplantation. The data are interesting and may have great translational implications in predicting the potential susceptibility of these patients to aGVHD.

Major points.

1) In Figure 3, did the authors assess what the effect of the src inhibitor had on IFN-gamma secretion by NK cells and effect on specific lysis (as in Figure 4C)? Did they also assess potential cellualr toxicity of the src inhibitor on NK cells?

2) The authors should perform an ELISPOT assay to determine the number of NK cells secreting IFN-gamma in the presence of MICA-129met versus MICA-129val. This would be more informative than looking at overall IFN-gamma secretion and analysis by ELISA. Also, description of Figure 4D is missing in the Figure Legend.

3) In Figures 6B and 7C, the authors should include the untreated, CFSE cells to show what the starting labeled levels on non-proliferating cells looked like. In addition, the authors should include % proliferation and not MFI levels.

4) In Figure 7A, the authors should include the flow cytometric data showing CD3, CD8, and NKG2D co-staining.

Referee #2 (Comments on Novelty/Model System):

The technical quality is in principle high but unfortunately, the most relevant NK cell subsets were not addressed separately but, instead, given as results of total NK cell with respect to cytotoxicity, IFN-g production and NKG2D down regulation. Since this important information is available by CD56, CD16 and CD94 staining, it should be possible to re-evaluate the data according to the CD56dm and CD56bright NK cell subsets. The relevance for this MICA polymorphism in Stem cell transplantation is novel. However, due to the complexity of stem cell transplantation with repect to conditioning, underlying disease etc., it is unlikely that a single polymorphism in the MICA gene is alone causing the observed effects in Figure 1. Therefore, MICA genotyping is certainly of scientific interest but the clinical relevance should be evaluated in light of both the complexity in stem cell transplantation and in NK cell regulation.

Referee #2 (Remarks):

The manuscript by Isernhagen et al. addresses the influence of a single polymorphism at amino acid 129 in the NKG2D-ligand, MICA, i.e. MICA-129Met vs. MICA-129Val on outcome after hematopoietic stem cell transplantation and on NK cell function like degranulation, and IFN-g

secretion.
The authors can nicely demonstrate that this MICA-polymorphism has clinical impact on survival, acute, chronic GVHD and relapse with the MICA-129Met allele increasing overall survival despite of an increased risk for aGVHD. In contrast, ATG-treatment of MICA-129Val carriers was associated with their increased survival. Functionally, this MICA polymorphism could be associated with enhanced binding, induction of cytotoxicity and IFN-g secretion of the MICA-129Met allele. With respect to T cell co-stimulation, this allele also induced faster T cell stimulation.
Although the association of this MICA-polymorphism with survival after stem cell transplantation, NK cell activation and T cell co-stimulation is interesting and relevant, there are some aspects that could be addressed more specifically in order to define the NK cell effect more precisely.
Major comments:
1. In general, isolated NK cells were pre-activated 4 days with IL-2 before testing them for CD107a degranulation, induction of cytotoxicity, and IFN-g secretion. However, cytokine stimulation with IL-2 for 4 days generally enhances NK cell activation at all levels. Therefore, the influence of these two alleles in transfected L cells as target cells should be demonstrated with freshly isolated NK cells - et least exemplarily.
2. Throughout the manuscript, activity of all NK cells is shown without looking at specific NK cell subsets. Since the two major subsets of peripheral NK cells, i.e. CD56dimCD16+ and CD56bright CD16- NK cells have been shown to differ also functionally, these two subsets should be nalayzed also separately. Since in the methods section, several NK cell markers such as CD16, CD94, NKp30, NKp44 and NKp46 are mentioned as part of the phenotyping FACS panel, it should be possible at least distinguish between these two subsets. In case of the known disappearance of the CD56brightCD16- NK subsets during the 4 day-culture period with IL-2, the results would apply for the CD56dimCD16+ NK cell subset which could then nicely be demonstrated. Therefore, FACS plots should be added to Figures 2showing CD107a vs. CD56 or CD16 staining or differential gating on the two major NK cell subsets.
3. The CD94 receptor in combination with the inhibitory NKG2A or the activating NKG2C receptor represents an important functional NK cell marker due to its binding to HLA-E. Therefore, it should be demonstrated whether activation with the two MICA alleles has also an impact on CD94 expression following stimulation.

Minor comments:
With respect to cytokines, only IFN-g was analyzed. If these data are available, other effector cytokines like TNF-a, IL-10 or IL-13 should also be included into the manuscript even there were no significant differences between the two MICA alleles.

Referee #3 (Comments on Novelty/Model System):

This is an important translational study which really provides new information because the association of MICA variants (Val/Val or Met/Met) with cGVHD and /or aGVHD after HSCT have been known for some time but the mechanisms had not at all been elucidated.
The data represent a major advance in the knowledge of how NK cell function can be modified by NKG2D interaction with different MICA alleles as both regulation of NKG2D mediated activation signals and regulation of NKG2D expression at the plasma membrane are demonstrated. The relatiionship betwwen expression density and function of NKG2D and MICA is revealed.
The technical quality of the paper is very high.
The experiments have been carefully planned and the data reported in this paper strongly support the conclusions of the paper.

Referee #3 (Remarks):

The paper provides novel information about the regulation of MICA function in relation to the polymorphism of this molecule.
The data represent a major advance in the knowledge of how NK cell function can be modified by NKG2D interaction with different MICA alleles as both regulation of NKG2D mediated activation

signals and regulation of NKG2D expression at the plasma membrane are demonstrated.

Minor points
In the Discussion, the authors mention the expression of MICA by non-professional antigen presenting cells which activate alloreactive CD8+ T cells. Some discussion of the cell types implicated (endothelial ? epithelial ? tumoral ?) would be informative and particularly in the context of GVHD.

The potential for ATG to target cell types, other than T lymphocytes, should be mentionned as non-T lymphocytes may also be implicated in the results seen in patients receiving ATG.

The title of Figure 4 should be reconsidered

1st Revision - authors' response 20 July 2015

Response to the reviewer's comments

We would like to thank the reviewers for their overall encouraging comments for our study and their most valuable criticisms. In addition to addressing the comments of the reviewers, we have included in the revised version of the manuscript in Figure 1 the panels G and H to better illustrate the therapeutic effect of anti-thymocyte globulin in the homozygous carriers of the MICA-129Val genotype in our cohort. Please find below the reviewer's comments and our answers (in italics).

***** Reviewer's comments *****

Referee #1 (Comments on Novelty/Model System):

These findings are extremely interesting, especially when looking at the correlation of the survival curves with MICA-129 dimorphisms. The findings suggest that analysis for MICA-129met may have diagnostic potential for predicting survivability from aGVHD following HSC transplantation.

Referee #1 (Remarks):

In the manuscript entitled "The MICA-129 dimorphism affects NKG2D signaling and outcome of hematopoietic stem cell transplantation," the authors present correlative data that seem to show better survival outcomes in patients that show the presence of the MICA-129met versus MIVA-129val following HSC transplantation. The data are interesting and may have great translational implications in predicting the potential susceptibility of these patients to aGVHD.

Major points.

1) In Figure 3, did the authors assess what the effect of the src inhibitor had on IFN-gamma secretion by NK cells and effect on specific lysis (as in Figure 4C)? Did they also assess potential cellular toxicity of the src inhibitor on NK cells?

Answer:

We also determined the effect of the SRC kinase inhibitor PP2 on target cell lysis by NK cells (Appendix Figure S5A) and the release of IFNγ and TNFα (Appendix Figure S5B). In addition to

NK cell degranulation, also these effector functions, which can be triggered by NKG2D, were completely inhibited. Moreover, we assessed the potential cellular toxicity of PP2 (25 µM for 4 h) by Annexin V/propidium iodide staining (Appendix Figure S5C, D) and found no increase of apoptotic NK cells at least within 4 h, the time period used for these experiments.

2) The authors should perform an ELISPOT assay to determine the number of NK cells secreting IFN-gamma in the presence of MICA-129met versus MICA-129val. This would be more informative than looking at overall IFN-gamma secretion and analysis by ELISA. Also, description of Figure 4D is missing in the Figure Legend.

Answer:

We completely agree that information on the proportion of NK cells producing IFNγ is important. Since the reviewer #2 requested information on the NK cell subpopulations, which produce IFNγ in response to NKG2D engagement, we performed intracellular flow cytometry instead of ELISPOT assays to be able to address both questions. We could show that mainly $CD56^{bright}CD16^{-}$ and to a lesser extent $CD56^{bright}CD16^{+}$ NK cells secrete IFNγ in response to NKG2D engagement (Figure EV3). The proportion of NK cells reacting by IFNγ expression upon stimulation by MICA-129Met-Fc and MICA-129Val-Fc proteins is shown in Figure EV3B. At the used (relatively low) concentrations more $CD56^{bright}CD16^{-}$ NK cells produced IFNγ in response to the MICA-129Met-Fc than the MICA-129Val-Fc protein (P=0.0025; ANOVA). In co-culture experiments with L cells expressing the MICA-129 variants, the percentage of IFNγ-expressing NK cells increased with expression intensity of the MICA-129Val isoform (Figure 4A, Appendix Figure S7A, B). It did not increase for NK cells exposed to the L-MICA-129Met clones (Figure 4A). On $CD56^{bright}CD16^{+}$ NK cells, the proportion of $IFN\gamma^{+}$ cells even decreased with increased MICA expression intensity (Appendix Figure S7A). A description of Figure 4D (now Figure 4B) is provided.

3) In Figures 6B and 7C, the authors should include the untreated, CFSE cells to show what the starting labeled levels on non-proliferating cells looked like. In addition, the authors should include % proliferation and not MFI levels.

Answer:

In these figures (still Figure 6B and 7C) the untreated CFSE stained cells are now included. We also added the percentage of proliferating cells. A detailed analysis of the number of cell divisions is provided in the Appendix Figure S14.

4) In Figure 7A, the authors should include the flow cytometric data showing CD3, CD8, and NKG2D co-staining.

Answer:

The CD3, CD8, and NKG2D co-staining is now shown in Figure EV5.

Referee #2 (Comments on Novelty/Model System):

The technical quality is in principle high but unfortunately, the most relevant NK cell subsets were not addressed separately but instead, given as results of total NK cell with respect to cytotoxicity, IFN-g production and NKG2D down regulation. Since this important information is available by CD56, CD16 and CD94 staining, it should be possible to re-evaluate the data according to the CD56dm and CD56bright NK cell subsets. The relevance for this MICA polymorphism in Stem cell transplantation is novel. However, due to the complexity of stem cell transplantation with respect to conditioning, underlying disease etc., it is unlikely that a single polymorphism in the MICA gene is

alone causing the observed effects in Figure 1. Therefore, MICA genotyping is certainly of scientific interest but the clinical relevance should be evaluated in light of both the complexity in stem cell transplantation and in NK cell regulation.

Referee #2 (Remarks):

The manuscript by Isernhagen et al. addresses the influence of a single polymorphism at amino acid 129 in the NKG2D-ligand, MICA, i.e. MICA-129Met vs. MICA-129Val on outcome after hematopoietic stem cell transplantation and on NK cell function like degranulation, and IFN-g secretion.

The authors can nicely demonstrate that this MICA-polymorphism has clinical impact on survival, acute, chronic GVHD and relapse with the MICA-129Met allele increasing overall survival despite of an increased risk for aGVHD. In contrast, ATG-treatment of MICA-129Val carriers was associated with their increased survival. Functionally, this MICA polymorphism could be associated with enhanced binding, induction of cytotoxicity and IFN-g secretion of the MICA-129Met allele. With respect to T cell co-stimulation, this allele also induced faster T cell stimulation.

Although the association of this MICA-polymorphism with survival after stem cell transplantation, NK cell activation and T cell co-stimulation is interesting and relevant, there are some aspects that could be addressed more specifically in order to define the NK cell effect more precisely.

Major comments:

1. In general, isolated NK cells were pre-activated 4 days with IL-2 before testing them for CD107a degranulation, induction of cytotoxicity, and IFN-g secretion. However, cytokine stimulation with IL-2 for 4 days generally enhances NK cell activation at all levels. Therefore, the influence of these two alleles in transfected L cells as target cells should be demonstrated with freshly isolated NK cells - at least exemplarily.

Answer:

Engagement of NKG2D alone is known not to be sufficient to induce the release of cytotoxic granules from resting NK cells (Bryceson et al, 2009). We have now included data (see page 8) showing that resting NK cells indeed failed to kill L-MICA-129Met and L-MICA-129Val cells in contrast to K562 cells (Appendix Figure S4A, B). Moreover, no degranulation (CD107a expression) or IFNγ release was elicited (Appendix Figure S4C, D). Exposure to MICA-expressing targets also failed to induce the release of TNFα, IL-10, and IL-13 from NK cells at least within the first 4 h of co-culture (Appendix Figure S4E). However, we have shown previously that NK cells can readily kill MICA-expressing L cells after being stimulated for 4 days with a low dose of IL-2 (Elsner et al, 2010). Therefore, we used IL-2-stimulated NK cells in the subsequent experiments. This allowed us to elicit NK cell effector functions by engagement of NKG2D only. A comparison of freshly isolated and IL-2-stimulated (100 U/ml for 4 days) NK cells with respect to NKG2D expression is now displayed in Figure EV1.

2. Throughout the manuscript, activity of all NK cells is shown without looking at specific NK cell subsets. Since the two major subsets of peripheral NK cells, i.e. CD56dimCD16+ and CD56bright CD16- NK cells have been shown to differ also functionally, these two subsets should be analyzed also separately. Since in the methods section, several NK cell markers such as CD16, CD94, NKp30, NKp44 and NKp46 are mentioned as part of the phenotyping FACS panel, it should be possible at least distinguish between these two subsets. In case of the known disappearance of the CD56brightCD16- NK subsets during the 4 day-culture period with IL-2, the results would apply for the CD56dimCD16+ NK cell subset which could then nicely be demonstrated. Therefore, FACS plots should be added to Figures 2showing CD107a vs. CD56 or CD16 staining or differential gating on the two major NK cell subsets.

Answer:

We agree with the reviewer that the differentiation of NK cell subpopulations with respect to their functional capabilities is very important. We have now differentiated $CD56^{dim}CD16^{+}$ and $CD56^{bright}CD16^{-}$ NK cells and the intermediate $CD56^{bright}CD16^{+}$ NK cell population. This was possible for the MACS purified IL-2-stimulated NK cells at day 4 although the borders between the populations were sometimes less clear than on freshly isolated NK cells. On IL-2-stimulated PBMC (i.e. LAK cells, which had been used before for some experiments) we could not clearly identify a $CD56^{bright}$ population that was sufficient in numbers for a reanalysis of data. However, we performed new experiments and analyzed the NKG2D expression, which was similar on the three NK cell populations (Figure EV1), degranulation, which was strongest in $CD56^{dim}CD16^{+}$ NK cells (Figure EV2), IFNγ expression, which was strongest in $CD56^{bright}CD16^{-}$ NK cells (Figure EV3), and NKG2D-downregulation, which occurred similarly on all three NK cell populations in response mainly to the MICA-129Met variant (Figure EV4). The $CD56^{bright}CD16^{+}$ NK cells had an intermediate phenotype with respect to degranulation and IFNγ production.

3. The CD94 receptor in combination with the inhibitory NKG2A or the activating NKG2C receptor represents an important functional NK cell marker due to its binding to HLA-E. Therefore, it should be demonstrated whether activation with the two MICA alleles has also an impact on CD94 expression following stimulation.

Answer:

We had included CD94 as control for NKG2D in co-culture experiments with L-MICA-129Met and L-MICA-129Val clones (now Appendix Figure S9). These experiments demonstrated that CD94 expression was not altered in response to engagement of NKG2D by the two MICA isoforms on L cells.

Minor comments:

With respect to cytokines, only IFN-g was analyzed. If these data are available, other effector cytokines like TNF-a, IL-10 or IL-13 should also be included into the manuscript even there were no significant differences between the two MICA alleles.

Answer:

We have included now a data set showing that also TNFα production was stimulated after engagement of NKG2D by the two MICA-129 isoforms (Appendix Figure S8). We found a similar difference of the MICA-129 variants to induce production of TNFα as for IFNγ. IL-10 production was not elicited (at least within 4 h) and IL-13 production even appeared to decrease upon engagement of NKG2D by both MICA-129 variants.

Referee #3 (Comments on Novelty/Model System):

This is an important translational study, which really provides new information because the association of MICA variants (Val/Val or Met/Met) with cGVHD and /or aGVHD after HSCT have been known for some time but the mechanisms had not at all been elucidated.

The data represent a major advance in the knowledge of how NK cell function can be modified by NKG2D interaction with different MICA alleles as both regulation of NKG2D mediated activation signals and regulation of NKG2D expression at the plasma membrane are demonstrated. The relatiionship betwwen expression density and function of NKG2D and MICA is revealed.

The technical quality of the paper is very high.

The experiments have been carefully planned and the data reported in this paper strongly support the conclusions of the paper.

Referee #3 (Remarks):

The paper provides novel information about the regulation of MICA function in relation to the polymorphism of this molecule.

The data represent a major advance in the knowledge of how NK cell function can be modified by NKG2D interaction with different MICA alleles as both regulation of NKG2D mediated activation signals and regulation of NKG2D expression at the plasma membrane are demonstrated.

Minor points

In the Discussion, the authors mention the expression of MICA by non-professional antigen presenting cells, which activate allo-reactive CD8+ T cells. Some discussion of the cell types implicated (endothelial? epithelial? tumoral?) would be informative and particularly in the context of GVHD.

Answer:

We thank the reviewer for this suggestion and we have now briefly discussed this issue (see page 17).

The potential for ATG to target cell types, other than T lymphocytes, should be mentioned as non-T lymphocytes may also be implicated in the results seen in patients receiving ATG.

Answer:

We agree with the reviewer and have mentioned now that ATG can affect other immune cell populations beside T cells (see page 19).

The title of Figure 4 should be reconsidered

Answer:

The title of Figure 4 has been modified.

2nd Editorial Decision 11 September 2015

Thank you for the submission of your revised manuscript to EMBO Molecular Medicine. We have now received the enclosed reports from the referees that were asked to re-assess it. As you will see the reviewers are now supportive and I am pleased to inform you that we will be able to accept your manuscript pending some final editorial amendments.

***** Reviewer's comments *****

Referee #1 (Comments on Novelty/Model System):

From the initial review, I found the findings extremely interesting and novel - using MICA-129met as a diagnostic tool for predicting survivability from aGVHD following HSC transplantation.

Referee #1 (Remarks):

The authors responded adequately and in detail to my initial comments and questions.

Referee #2 (Comments on Novelty/Model System):

The in vitro studies support the hypothesis of a functional relevance of the MICA polymorphism for NK cell function

Referee #2 (Remarks):

With the response to the reviewer's comments and the data privided by the Appendix, the authors have answered all comments and questions properly.
